# Bioinformatics applications on Apache Spark

**DOI:** 10.1093/gigascience/giy098

**Published:** 2018-08-07

**Authors:** Runxin Guo, Yi Zhao, Quan Zou, Xiaodong Fang, Shaoliang Peng

**Affiliations:** 1 College of Computer, National University of Defense Technology, No.109, Deya Road, Kaifu District, Changsha, 410073, China; 2 Institute of Computing Technology, Chinese Academy of Sciences, No.6, South Road of the Academy of Sciences, Haidian District, Beijing, 100190, China; 3 School of Computer Science and Technology, No.135, Yaguan Road, Jinnan District, Tianjin University, Tianjin, 300050, China; 4 BGI Genomics, BGI-Shenzhen, No.21, Mingzhu Road, Yantian District, Shenzhen, 518083, China; 5 College of Computer Science and Electronic Engineering & National Supercomputer Centre in Changsha, Hunan University, No.252, Shannan Road, Yuelu District, Changsha, 410082, China

**Keywords:** next-generation sequencing, bioinformatics, Apache Spark, resilient distributed dataset, memory computing

## Abstract

With the rapid development of next-generation sequencing technology, ever-increasing quantities of genomic data pose a tremendous challenge to data processing. Therefore, there is an urgent need for highly scalable and powerful computational systems. Among the state-of–the-art parallel computing platforms, Apache Spark is a fast, general-purpose, in-memory, iterative computing framework for large-scale data processing that ensures high fault tolerance and high scalability by introducing the resilient distributed dataset abstraction. In terms of performance, Spark can be up to 100 times faster in terms of memory access and 10 times faster in terms of disk access than Hadoop. Moreover, it provides advanced application programming interfaces in Java, Scala, Python, and R. It also supports some advanced components, including Spark SQL for structured data processing, MLlib for machine learning, GraphX for computing graphs, and Spark Streaming for stream computing. We surveyed Spark-based applications used in next-generation sequencing and other biological domains, such as epigenetics, phylogeny, and drug discovery. The results of this survey are used to provide a comprehensive guideline allowing bioinformatics researchers to apply Spark in their own fields.

## Introduction

Next-generation sequencing (NGS) technology has generated huge amounts of biological sequence data. To use these data efficiently, we need accurate and efficient methods of storing and analyzing such data. However, the existing bioinformatics tools cannot effectively handle such a large amount of data. Therefore, there is an urgent need for scalable and powerful distributed computing tools to solve this problem. In the field of information technology, MapReduce [1] is a distributed parallel programming model and methodology for processing large-scale datasets. It splits large-scale datasets into many key-value pairs through both the map and reduce phases, significantly improving performance and showing good scalability. By combining the Hadoop Distributed File System (HDFS) and MapReduce, Apache Hadoop can enable distributed processing of large amounts data in a reliable, efficient, and scalable way. This is in contrast to HDFS, which is mainly used for distributed storage of massive datasets, and MapReduce, which performs distributed computing on these datasets. As a result, Hadoop has been adopted by the bioinformatics community in several areas [2], including alignment [3-6], mapping [7-9], and sequence analysis [10-13].

Because of Hadoop's disk-based input output system (I/O) access pattern, however, intermediate calculation results are not cached. Therefore, Hadoop is only suitable for batch data processing and shows poor performance for iterative data processing. To resolve this problem, Apache Spark [14] has been proposed; a faster, general-purpose computing framework specifically designed to handle huge amounts of data. Unlike Hadoop's disk-based computing, Spark performs memory computing by introducing resilient distributed dataset (RDD) abstraction. Since it is possible to store intermediate results in memory, it is more efficient for iterative operations. In terms of performance, Spark can be up to 100 times faster in terms of memory access than Hadoop [14]. The gap between Spark and Hadoop is more than 10-fold greater, even if we compare between them based on disk performance [15]. In terms of flexibility, Spark provides high-level application programming interfaces (APIs) in Java, Scala, Python, and R, and interactive shell. In terms of generality, Spark provides structured data processing, machine learning, graph computing, and stream computing capabilities by supporting some advanced components. Table 1 summarizes the bioinformatics tools and algorithms based on Apache Spark.

**Table 1:** Bioinformatics tools and algorithms based on Apache Spark

Name	Function	Features	Pros/Cons	Reference
SparkSW	Alignment and mapping	Consists of three phases: data preprocessing, SW as map tasks, and top K records as reduce tasks	Load-balancing, scalable, but without the mapping location and traceback of optimal alignment	[16]
DSA	Alignment and mapping	Leverages data parallel strategy based on SIMD instruction	Up to 201 times increased speed over SparkSW and almost linearly increased speed with increasing numbers of cluster nodes	[17]
CloudSW	Alignment and mapping	Leverages SIMD instruction, and provides API services in the cloud	Up to 3.29 times increased speed over DSA and 621 times increased speed over SparkSW; high scalability and efficiency	[18]
SparkBWA	Alignment and mapping	Consists of three main stages: RDD creation, map, and reduce phases; employs two independent software layers	For shorter reads, averages 1.9x and 1.4x faster than SEAL and pBWA. For longer reads, averages 1.4x faster than BigBWA and Halvade, but requires the data availability in HDFS	[19]
StreamBWA	Alignment and mapping	Input data are streamed into the cluster directly from a compressed file	∼2x faster than nonstreaming approach, and 5x faster than SparkBWA	[20]
PASTASpark	Alignment and mapping	Employs an in-memory RDD of key-value pairs to parallel the calculating MSA phase	Up to 10x faster than single-threaded PASTA; ensures scalability and fault tolerance	[21]
PPCAS	Alignment and mapping	Based on the MapReduce processing paradigm in Spark	Better with a single node and shows almost linearly increased speeds with increasing numbers of nodes	[22]
SparkBLAST	Alignment and mapping	Utilizes *pipe* operator and Spark RDDs to call BLAST as an external library	Outperforms CloudBLAST in terms of speed, scalability, and efficiency	[23]
MetaSpark	Alignment and mapping	Consists of five steps: constructing *k*-mer RefindexRDD, constructing *k*-mer ReadlistRDD, seeding, filtering, and banded alignment	Recruits significantly more reads than SOAP2, BWA, and LAST and more reads by ∼4 than FR-HIT; shows good scalability and overall high performance	[24]
Spaler	Assembly	Employs Spark's GraphX API; consists of two main parts: de Bruijn graph construction and contig generation	Shows better scalability and achieves comparable or better assembly quality than ABySS, Ray, and SWAP-Assembler	[25]
SA-BR-Spark	Assembly	Under the strategy of finding the source of reads; based on the Spark platform	Shows a superior computational speed than SA-BR-MR	[26]
HiGene	Sequence analysis	Puts forward a dynamic computing resource scheduler and an efficient way of mitigating data skew	Reduces total running time from days to just under nearly an hour; 2x faster than Halvade	[27]
GATK-Spark	Sequence analysis	Takes full account of compute, workload, and characteristics	Achieves more than 37 times increased speed	[28]
SparkSeq	Sequence analysis	Builds and runs genomic analysis pipelines in an interactive way by using Spark	Achieves 8.4–9.15 times faster speeds than SeqPig; accelerates data querying up to 110 times and reduces memory consumption by 13 times	[29]
CloudPhylo	Phylogeny	Evenly distributes entire workloads between worker nodes	Shows good scalability and high efficiency; the Spark version is better than the Hadoop version	[30]
S-CHEMO	Drug discovery	Intermediate data are immediately consumed again on the producing nodes, saving time and bandwidth	Shows almost linearly increased speeds on up to eight nodes compared with the original pipeline	[31]
Falco	Single-cell RNA sequencing	Consist of a splitting step, an optional preprocessing step, and the main analysis step	At least 2.6x faster than a highly optimized single-node analysis; running time decreases with increasing numbers of nodes	[32]
VariantSpark	Variant association and population genetics studies	Parallels population-scale tasks based on Spark and the associated MLlib	80% faster than ADAM, Hadoop/Mahout version, and ADMIXTURE; more than 90% faster than R and Python implementations	[33]
SEQSpark	Variant association and population genetics studies	Splits large-scale datasets into many small blocks to perform rare variant association analyses	Always faster than Variant Association Tools and PLINK/SEQ; in some cases, running time is reduced to 1%	[34]
BioSpark	Data-parallel analysis on large, numerical datasets	Consists of a set of Java, C++, and Python libraries; abstractions for parallel analysis of standard data types; some APIs; and file conversion tools	Convenient, scalable, and useful; has domain-specific features for biological applications	[35]

## The Spark Framework

Designed and developed by the Algorithms, Machines and People Lab at the University of California, Berkeley, Spark is an open-source cluster computing environment designed for large-scale data processing. It provides advanced APIs in Java, Scala, Python, and R and an optimized engine that supports general execution graphs. It also supports some advanced components, including Spark SQL for structured data processing, MLlib for machine learning, GraphX for computing graphs, and Spark Streaming for stream computing.

**Figure 1:**
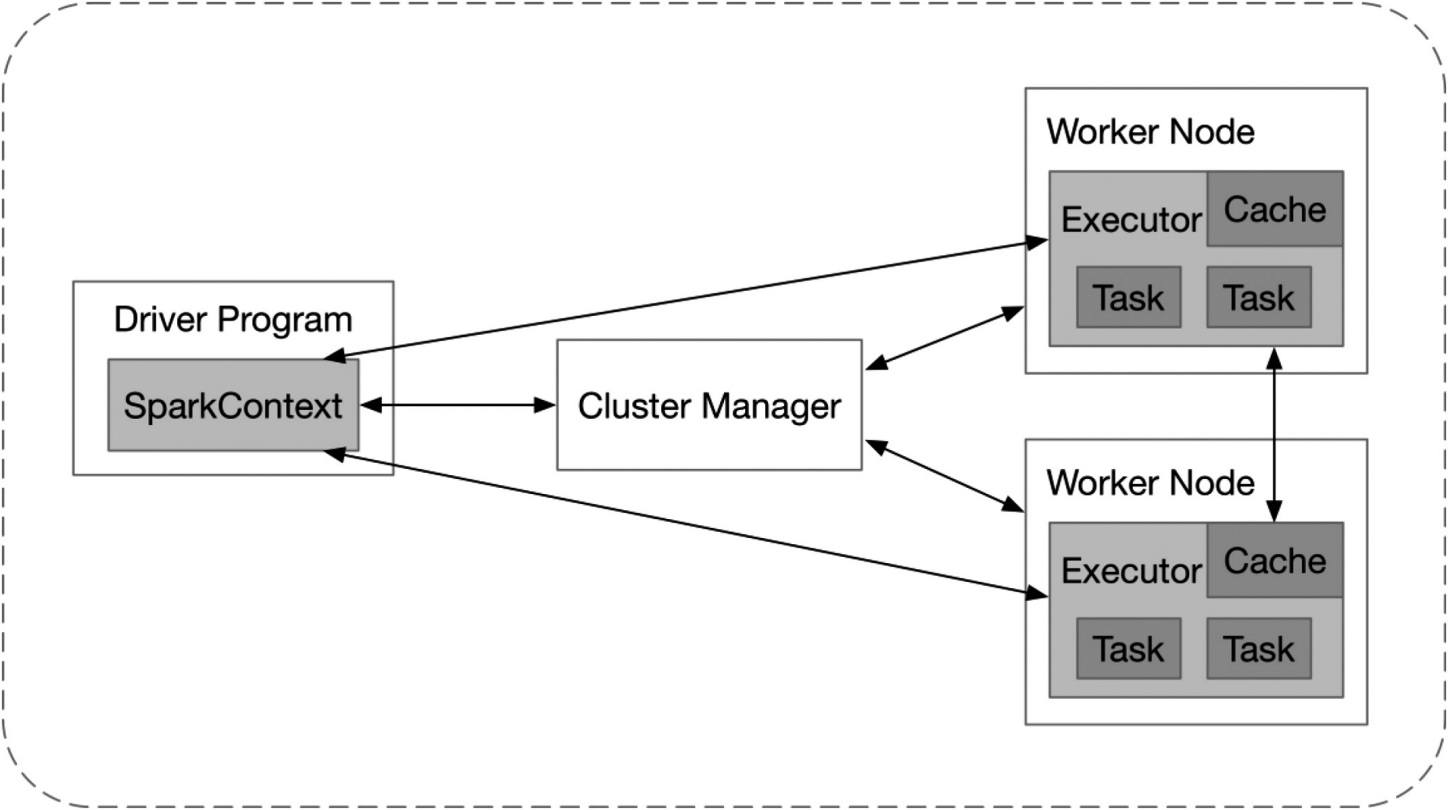
The cluster architecture of Spark.

As shown in Fig. 1, each Spark application runs as an independent process on the cluster, coordinated by SparkContext in the driver program. There are two deploy modes, depending on where the driver program is running: cluster mode and client mode. In the former mode, the driver program runs on a worker node. In the latter, the driver program runs on the client machine. First, SparkContext requests the executors on the worker nodes in the cluster from the cluster manager (either Spark's own stand-alone cluster manager, Apache Mesos, or Hadoop YARN). These executors are processes that can run tasks and store data in memory or on disk for application. Next, SparkContext will send tasks to the executors to perform. Finally, the executors return the results to SparkContext after the tasks are executed. In Spark, an application generates multiple jobs. A job is split into several stages. Each stage is a task set containing several tasks, which performs calculations and produces intermediate results. A task is the smallest unit of work in Spark, completing a specific job on an executor. As for deployment of the Spark cluster, the official proposal for hardware requirements is to have 4–8 disks per node, which configure at least 8 GB of memory and 8–16 central processing unit (CPU) cores per machine, and to use a 10-gigabit or higher network.

**Figure 2:**
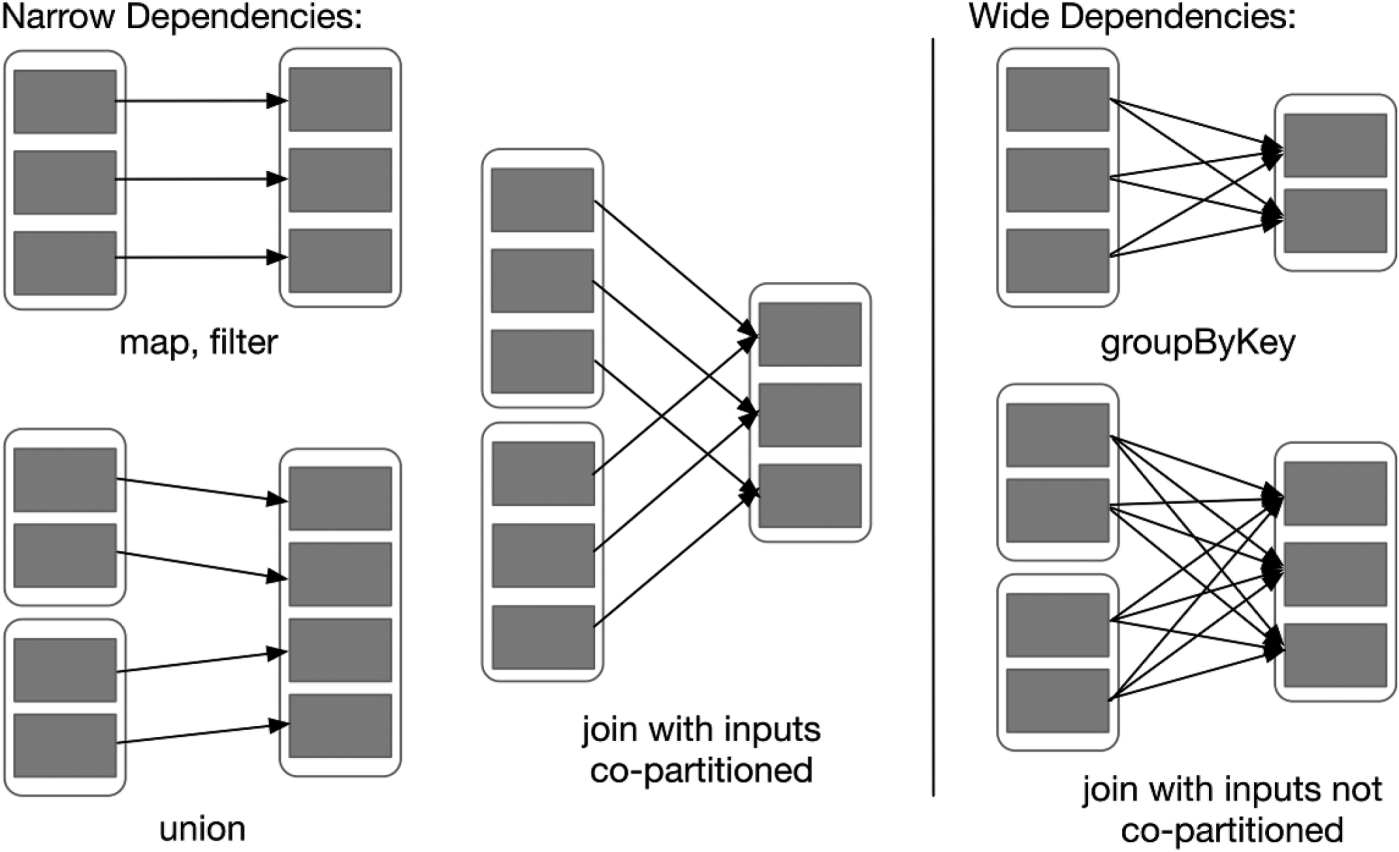
Examples of narrow and wide dependencies. Each box is an RDD, where the partition is shown as a shaded rectangle.

As the main abstraction in Spark, RDD is a read-only collection of objects partitioned on different nodes in the cluster so that the data in RDD can be processed in parallel. The data in RDD are stored in memory by default, but Spark automatically writes RDD data to disk if memory resources are low. RDD achieves fault tolerance through a notion of lineage [14]; i.e., if an RDD partition on a node is lost because of a node failure, the RDD automatically recalculates the partition from its own data source. Moreover, Spark provides two types of operations on RDD: transformation and action. The former defines a new RDD, and the latter returns a result or writes RDD data to the storage system. Transformation employs lazy operation [36], which means that the operation of generating another RDD from one RDD transformation is not executed immediately, and the calculation process is not actually started until an action is performed. Furthermore, each transformation operation generates a new RDD; the newly generated RDD depends on the original RDD. According to the different types of transformation operation, RDD dependencies can be divided into narrow dependency and wide dependency. The former refers to the fact that each partition in the generated RDD depends only on the parent RDD fixed partition, and the latter refers to the fact that each partition of the generated RDD depends on all partitions of the parent RDD. Figure 2 shows examples of narrow and wide dependencies. In addition, Spark also provides two extensions of RDD: DataFrame and Dataset. Spark users can seamlessly switch between these through simple API calls.

**Figure 3:**
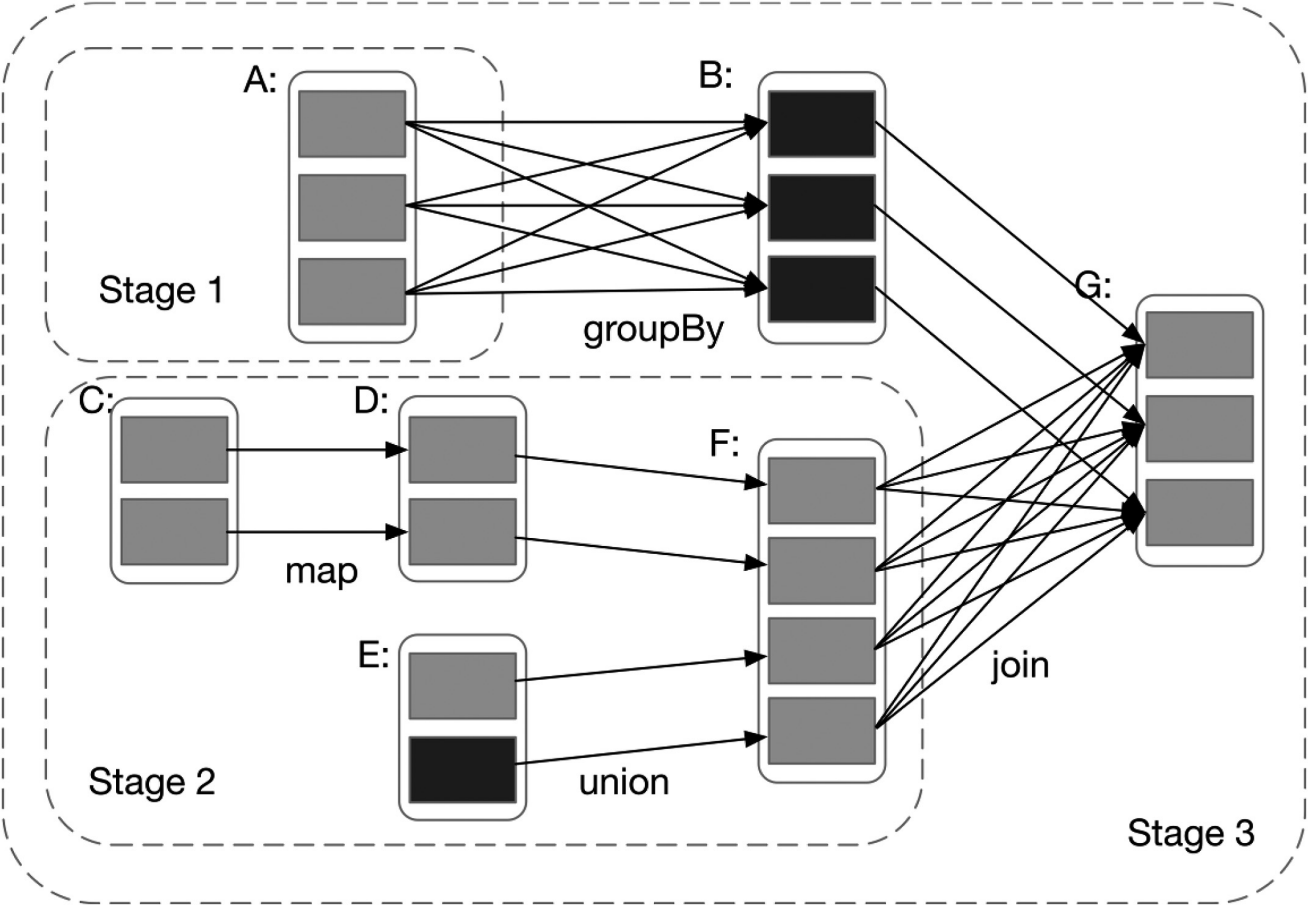
An example of how Spark computes job stages. Boxes with solid outlines are RDDs. Partitions are shaded rectangles and are black if they are already in memory. To run an action on RDD G, we build stages at wide dependencies and pipeline narrow transformation inside each stage. In this case, the output RDD of stage 1 is already in memory, so we run stage 2 and then stage 3.

Spark also adopts a directed acyclic graph (DAG) [37] to optimize execution processes by splitting submitted jobs into several stages according to wide dependency. For narrow dependency, it divides related transformation operations into the same stage; this is because they can perform pipelining operations and thus reduce the processing time of submitted jobs. Figure 3 shows an example of how Spark computes job stages. In addition, if the partitions on a node are lost because of node failure, Spark can utilize the DAG to recalculate the lost partitions.

## Spark in Alignment and Mapping

The rapid development of NGS technology has generated a large amount of sequence data (reads), which has a tremendous impact on sequence alignment and mapping processes. Currently, sequence alignment and mapping remain time consuming.

The Smith–Waterman (SW) algorithm [38], which produces optimal local alignment between two strings of nucleic acid sequences or protein sequences, is widely used in bioinformatics. However, this algorithm has a high computational cost because of high computational complexity. To speed up the algorithm, Zhao et al. (2015) [16] implemented the SW algorithm on Spark for the first time, naming this SparkSW. It consisted of three phases: data preprocessing, SW as map tasks, and top K records as reduce tasks. Experimental results [16] showed that SparkSW was load-balancing and scalable with increased computing resources. However, SparkSW merely supports the SW algorithm without the mapping location and traceback of optimal alignment. As a result, SparkSW executes slowly. Therefore, Xu et al. (2017) proposed DSA [17], which employed single instruction multiple data (SIMD) to parallel the sequence alignment algorithm at each worker node. Experimental results [17] showed that DSA achieved up to 201 times faster speeds over SparkSW and almost linearly increased speed with increased cluster nodes. Subsequently, Xu et al. proposed CloudSW [18], an efficient distributed SW algorithm that leveraged Spark and SIMD instructions to accelerate the algorithm and provided API services in the cloud. Experimental results [18] showed that CloudSW achieved up to 3.29 times increased speed over DSA and 621 times increased speed over SparkSW. CloudSW also showed excellent scalability and achieved speeds of up to 529 giga cell updates per second in a protein database search with 50 nodes using Aliyun cloud.

The Burrows–Wheeler aligner (BWA) is composed of BWA-backtrack [39], BWA-SW [40], and BWA-MEM [41] for performing sequence alignment and mapping in bioinformatics. Before the advent of Spark-based BWA tools, there were several other BWA tools based on big data technology, including BigBWA [42], Halvade [43], and SEAL [7]. However, these were based on Hadoop, which showed limited scalability and complex implementation. As a result, Al-Ars et al. [(2015) 44] implemented three versions of BWA-MEM and compared their performance: a native cluster-based version, a Hadoop version, and a Spark version. Three implementations were evaluated on the same IBM Power7 and Intel Xeon servers, with WordCount as an example. The results [44] showed that simultaneous multithreading improved the performance of three versions of BWA-MEM, and the Spark version with 80 threads increased performance by up to 87% over the native cluster version using 16 threads. Furthermore, the four-thread Hadoop version increased performance by 17%, and the Spark version with even more threads increased performance by 27%. Then, in 2016, Abuín et al. proposed SparkBWA [19], which is composed of three main phases: the RDDs creation phase, the map phase, and the reduce phase. Experimental results [19] showed that for the BWA-backtrack algorithm, SparkBWA achieved average increased speeds of 1.9 times and 1.4 times compared with SEAL and pBWA, respectively. For the BWA-MEM algorithm, SparkBWA was, on average, 1.4 times faster than BigBWA and Halvade tools. However, SparkBWA required significant time to preprocess the input files and finally combine the output files. Therefore, in 2017, Mushtaq et al. proposed StreamBWA [20], in which the input files were streamed into the Spark cluster. This greatly reduced the time required to preprocess data and combine the final results. Experimental results [20] showed that this streaming distributed strategy gave roughly double the speed of the nonstreaming strategy. Furthermore, StreamBWA achieved a five-fold increased speed over SparkBWA.

Multiple sequence alignment (MSA) refers to the sequence alignment of three or more biological sequences, such as protein or nucleic acid sequences. One representative tool for performing MSA is PASTA [45]. PASTA is a derivative of SATé [46], which produces highly accurate alignments in shared memory computers. However, PASTA is limited to processing small and medium datasets because the computing power of shared memory systems cannot meet the memory and time requirements of large-scale datasets. Therefore, in 2017, Abuín et al. proposed PASTASpark [21], which allowed executions on a distributed memory cluster, taking advantage of Spark. It employed an in-memory RDD of key-value pairs to parallel the calculating MSA phase. Experiments were conducted on two d clusters: Centro de Supercomputación de la Galicia and Amazon Web Services (AWS). The results [21] showed that PASTASpark achieved up to 10 times faster speeds than single-threaded PASTA and was able to process 200,000 sequences in 24 hours using only AWS nodes. Therefore, PASTASpark ensured scalability and fault tolerance, which greatly reduced the time to obtain MSA.

The probabilistic pairwise model [47] is widely used in all consistency-based MSA tools, such as MAFFT [48], ProbCons [49], and T-Coffee [50]. However, global distributed memory cannot meet the demands of ever-increasing sequence datasets, which leads to the need for specialized distributed databases, such as HBase or Cassandra. As a result, Lladós et al. (2017) employed Spark to propose a new tool, PPCAS [22], which could parallel the probabilistic pairwise model for large-scale protein sequences and store it in a distributed platform. Experimental results [22] showed that it was better with a single node and provided almost linearly increased speeds with the increased numbers of nodes. In addition, it could compute more sequences using the same amount of memory.

The National Center for Biotechnology Information's (NCBI's) Basic Local Alignment Search Tool (BLAST) tool [51, 52] is widely used to implement algorithms for sequence comparison. Before the Spark-based BLAST was created, several other BLAST tools had been proposed, including mpiBLAST [53], GPU-BLAST [54], and CloudBLAST [55]. However, with the increasing amount of genomic data, these tools showed limited scalability and efficiency. As a result, Castro et al. (2017) proposed SparkBLAST [23], which utilized cloud computing and the Spark framework to parallel BLAST. In SparkBLAST, Spark's *pipe* operator and RDDs were utilized to call BLAST as an external library and perform scalable sequence alignment. It was compared with CloudBLAST on both Google and Microsoft Azure clouds. Experimental results [23] showed that SparkBLAST outperformed CloudBLAST in terms of speed, scalability, and efficiency.

Metagenomics is crucial for directly studying genetic material from environmental samples. Fragment recruitment is the process of aligning reads to reference genomes in metagenomics data analysis. In 2017, Zhou et al. proposed MetaSpark [24], which employed Spark to recruit metagenomics reads to reference genomes. MetaSpark utilized the RDD of Spark to cache datasets in memory and scaled well along dataset size increments. It consisted of five steps, including constructing *k*-mer RefindexRDD, constructing *k*-mer ReadlistRDD, seeding, filtering, and banded alignment. It was evaluated on a 10-node cluster, working under the Spark stand-alone module, in which each node contained an eight-core CPU and 16 GB random access memory. It employed about 1 million 75 bp Illumina read datasets and two references: 194 human gut genomes and bacterial genomes that were 0.616 Gb and 1.3 Gb in size, respectively. Experimental results [24] showed that MetaSpark recruited more reads than FR-HIT [56] with the same parameters and 1 million reads. MetaSpark recruited 501,856 reads to 0.616 Gb human gut genome references, while FR-HIT recruited 489,638 reads. MetaSpark increased recruited reads by 2.5%. When references changed to a 1.3 Gb bacterial genome, MetaSpark recruited 463,862 reads, while FR-HIT recruited 444,671 reads. MetaSpark increased recruited reads by 4%. Moreover, the results also showed that MetaSpark offered good scalability. Under a 0.616 Gb reference, the run time for 100,000 reads was 51 minutes under four nodes and decreased slightly to 23.5 minutes under 10 nodes. For the 1 million read datasets, MetaSpark would crash under four nodes because of limited memory. Under six nodes, it finished running after 312 minutes and would sharply decrease to 201 minutes under 10 nodes.

## Spark in Assembly

Because NGS read lengths are short (<500 bp), they must be assembled before further analysis, which is another important phase in the sequence analysis workflow. In general, there are two types of assembly: the reference assembly and de novo assembly. The assembly algorithm includes two categories: the overlap–layout–consensus (OLC) algorithm and the de Bruijn graph algorithm. The former is generally employed to assemble longer reads, while the latter performs well in assembling short reads.

Before Spark-based distributed memory *de novo* assemblers were created, although there were some assemblers (such as Ray [57], AbySS [58], and SWAP-Assembler [59]) based on message passing interface (MPI), they showed limited scalability, accuracy, and computational efficiency. Therefore, in 2015, Abu-Doleh et al. proposed Spaler [25], taking advantage of Spark and GraphX APIs. It consisted of two main parts: de Bruijn graph construction and contig generation. It was evaluated against other MPI-based tools in terms of quality, execution time, and scalability. Experimental results [25] showed that Spaler had better scalability and could achieve comparable or better assembly quality.

To resolve the large memory requirement problem of most OLC *de novo* assemblers, Paul et al. (2017) [60] employed string graph reduction algorithms, taking advantage of Spark. The proposed Spark algorithms were evaluated against a very large sample dataset. The results showed that this dataset was assembled by the proposed Spark algorithms using 15 virtual machines in 0.5 hours compared with the 7.5 hours achieved by the OLC-based Omega [61] assembler.

In addition, some new assembly algorithms have also been proposed, based on the Spark platform itself. In 2016, Pan et al. [62] put forward a new assembling algorithm based on Spark, which employed the method of matching K-2 bit to simplify the de Bruijn graph. This algorithm was evaluated using six groups of DNA data in the NCBI database. Experimental results [62] showed that this strategy not only solved the problem of low efficiency based on the MapReduce algorithm but also optimized the algorithm itself. The combination of these two aspects was very suitable for the large-scale assembly of DNA sequences. Moreover, the results also showed that the new Spark-based sequence-assembling algorithm ensured the accuracy of assembling results.

To address the problem of poor assembling precision and low efficiency, Dong et al. (2017) [26] proposed SA-BR-Spark, a new sequence assembly algorithm based on Spark. The authors first designed a precise assembling algorithm using the strategy of finding the source of reads based on the MapReduce and Eulerian path algorithm (SA-BR-MR). SA-BR-MR calculated 54 sequences, randomly selected from animal, plant, and microorganism sequences in the NCBI database, with base lengths ranging from hundreds to tens of thousands. The matching rates of all 54 sequences were 100%. For each species, the algorithm also summarized the range of K that made the matching rates 100%. To verify the range of K values of hepatitis C virus and related variants, the K values of eight randomly selected hepatitis C virus variants were calculated. The results confirmed that the range of K of hepatitis C and related variants in NCBI were correct. After that, SA-BR-Spark was put forward. Experimental results [26] showed that SA-BR-Spark provided superior computational speed compared with SA-BR-MR.

## Spark in Sequence Analysis

The GATK (Genome Analysis Toolkit) DNA analysis pipeline is widely used in genomic data analysis. Before Spark-based GATK tools were created, while several other tools had been developed to address the issue of scalability in the pipeline (such as Halvade [43] and Churchill [63]), they showed limited scalability, accuracy, and computational efficiency.

Therefore, in 2015, Mushtaq et al. [64] utilized Spark to propose a cluster-based GATK pipeline. To reduce the execution time, this approach kept data active in the memory between the map and reduce phases. By using active workload runtime statistics, it achieved a dynamic load-balancing algorithm that could better utilize system performance. Experimental results [64] showed that this method achieved 4.5 times increased speed compared with the multithreaded GATK pipeline on a single node. In addition, when executed on a four-node cluster, this approach was 63% faster than Halvade.

Then, in 2016, Deng et al. proposed HiGene [27], which employed Spark to enable multicore and multinode parallelization of the GATK pipeline. HiGene put forward a dynamic computing resource scheduler and an efficient data-skew mitigation method to improve performance. Experiments were conducted with the NA12878 whole human genome dataset. The results [27] showed that HiGene reduced the total running time from days to just under 1 hour. Furthermore, compared with Halvade, HiGene was also two times faster. Meanwhile, Li et al. employed Spark to propose GATK-Spark [28]. This paralleled the GATK pipeline by taking full account of compute, workload, and I/O characteristics. It was built on top of the ADAM format [65]. Experimental results [28] showed that GATK-Spark decreased the total running time from 20 hours to 30 minutes on 256 CPU cores, which achieved more than 37-fold increased speeds.

Spark provides the opportunity for interactive NGS data processing. In 2014, Wiewiórka et al. proposed SparkSeq [29] to build and run genomic analysis pipelines in an interactive way by using Spark. Experimental results showed that SparkSeq achieved 8.4–9.15 times faster speeds than SeqPig. Moreover, it could accelerate data querying by up to 110 times and reduce memory consumption by 13 times.

## Spark in Other Biological Applications

### Spark in epigenetics

CpG islands are important markers that are essential in epigenetics [66]. However, investigation of CpG islands and their structures remains challenging. Before Spark-based applications were developed, while several methods had been proposed to determine the CpG islands (such as bisulfite modification-based methods), they were time consuming and prohibitively expensive. Thus, Yu et al. [67] utilized Spark to propose a novel CpG box model and a Markov model to redefine and investigate the CpG island, which could greatly accelerate the analytic process. Experiments were conducted with human and mouse chromosome sequences; 24 chromosomes and 21 chromosomes, respectively. The results [67] showed that this cloud-assisted method had considerable accuracy and faster processing power (6–7 times faster with 10 cores) compared with sequential processing.

### Spark in phylogeny

Phylogeny reconstruction is important in molecular evolutionary studies but faces significant computational challenges. Before Spark-based tools were created, while several tools had been put forward for phylogeny reconstruction, they did not scale well, and there was a significant increase in the number of datasets. Therefore, in 2016, Xu et al. proposed CloudPhylo [30], a fast and scalable phylogeny reconstruction tool that made use of Spark. It evenly distributed the entire computational workload between working nodes. An experiment was conducted using 5,220 bacteria whole-genome DNA sequences. The results [30] showed that CloudPhylo took 24,508 seconds with one worker node, and it was able to scale well with increasing numbers of worker nodes. Moreover, CloudPhylo performed better than several existing tools when using more worker nodes. In addition, CloudPhylo achieved faster speeds on a larger dataset of about 100 Gb generated by simulation.

### Spark in drug discovery

The identification of candidate molecules that affect disease-related proteins is crucial in drug discovery. Although the Chemogenomics Project tries to identify candidate molecules using machine-learning predictor programs [68-70], these programs are slow and cannot be easily extended to multiple nodes. To migrate existing programs to multinode clusters without changing the original programs, Harnie et al. proposed S-CHEMO [31], using Spark. In S-CHEMO, the intermediate data are immediately consumed again on the nodes that generated the data, reducing time and network bandwidth consumption. Experiments [31] compared S-CHEMO with the original pipeline and showed almost linearly increased speeds on up to eight nodes. Moreover, this implementation also allowed easier monitoring.

### Spark in single-cell RNA sequencing

Single-cell RNA sequencing (scRNA-seq) is crucial for understanding biological processes. Compared with standard bulk RNA-seq experiments, scRNA-seq experiments typically generate a greater number of cell profiles. Although several RNA-seq processing pipelines are available (such as Halvade, SparkSeq, and SparkBWA), they cannot process large numbers of profiles. Therefore, Falco [32] was created to process large-scale transcriptomic data in parallel by using Hadoop and Spark. Experiments were conducted with two public scRNA-seq datasets. The results [32] showed that, compared with a highly optimized single-node analysis, Falco was at least 2.6 times faster. Moreover, as the number of computing nodes increased, running time decreased. Furthermore, it allowed users to employ the low-cost spot instances of AWS, which reduced the cost of analysis by 65%.

### Spark in variant association and population genetics studies

Effectively analyzing thousands of individuals and millions of variants is a computationally intensive problem. Traditional parallel strategies such as MPI/OpenMP show poor scalability. While Hadoop provides an efficient and scalable computing framework, it is heavily dependent on disk operations. Therefore, in 2015, O'Brien et al. proposed VariantSpark [33] to parallel population-scale tasks based on Spark and an associated machine-learning library, MLlib. Experiments that were conducted on 3,000 individuals with 80 million variants showed that VariantSpark was 80% faster than ADAM, the Hadoop/Mahout implementation, and ADMIXTURE [71]. Moreover, compared with R and Python implementations, it was more than 90% faster. In 2017, Di et al. proposed SEQSpark [34] to perform rare variant association analysis using Spark. It was evaluated with whole-genome and simulated exome sequence data. The former was completed in 1.5 hours and the latter in 1.75 hours. Moreover, it was always faster than Variant Association Tools and PLINK/SEQ; in some cases, running time was reduced to 1%.

### Spark in other works

Biological simulations and experiments produce a large number of numerical datasets, and in 2017, Klein et al. proposed Biospark [35] to process these data. Biospark was based on Hadoop and Spark, comprising a set of Java, C++, and Python libraries. In addition, it provided the abstractions for parallel analysis of standard data types, including multidimensional arrays and images. To facilitate parallel analysis of some common datasets, it also provided APIs and file conversion tools, including Monte Carlo, molecular dynamics simulations, and time-lapse microscopy.

## Discussion

Spark is an in-memory iterative computing framework designed for large-scale data processing. It is suitable for applications that require iterative operations on specific datasets; the greater the amount of data, the higher the computational intensity and the greater the benefits. When the data volume is small but the computational intensity is large, the benefit is relatively small. In addition, Spark is also suitable for applications where the amount of data is not particularly large, but real-time statistical analyses are required.

However, the nature of RDD means that Spark is not suitable for applications requiring asynchronous, fine-grained updates in execution, such as web service storage or incremental web crawlers and indexes. In addition, we must consider the potential complexity of creating and maintaining a Spark cluster. Moreover, when Spark runs on a commercial cloud-computing platform such as AWS, there is a certain delay in the transmission of large-scale datasets over the Internet. This issue does not exist when Spark runs on a local computer cluster. Furthermore, we need to learn a new API and perhaps even a new language (especially given the functional programming nature of the API).

Although Spark has been applied in some areas of bioinformatics and has achieved good results, its use in other areas, –such as proteomics, biomedical texts, and metabolomics, has not yet been explored. Moreover, as cloud computing and some web servers become more available, some issues must be considered, such as the time cost of large amounts of input data from local to remote servers in slow networks, cloud computing fees, data security, and privacy.

## Conclusion

With the rapid development of NGS technology, a large number of genomic datasets have been generated, which poses a great challenge to traditional bioinformatics tools. For this reason, we have summarized relevant works about the use of Spark in bioinformatics and have created a guideline on this topic. First, we make a comparison between Spark and Hadoop and then outline the Spark cluster architecture, programming model, and processing mechanism. Then, we survey the use of Spark-based applications in NGS and other biological domains. Our survey means that researchers who wish to become involved in this field can now obtain a general understanding of the use of Spark in bioinformatics.

In summary, Spark is a fast and general-purpose computing framework designed for large-scale data processing. It ensures high fault tolerance and high scalability by introducing RDD abstraction and DAG scheduling. We believe that bioinformatics applications based on Spark will show promise in terms of performance for biological researchers in the future.

## Supplementary Material

GIGA-D-18-00131_Original_Submission.pdf

GIGA-D-18-00131_Revision_1.pdf

GIGA-D-18-00131_Revision_2.pdf

GIGA-D-18-00131_Revision_3.pdf

Response_to_Reviewer_Comments_Original_Submission.pdf

Response_to_Reviewer_Comments_Revision_1.pdf

Response_to_Reviewer_Comments_Revision_2.pdf

Reviewer_1_Report_(Original_Submission) -- Brendan Lawlor 4/29/2018 Reviewed

Reviewer_1_Report_Revision_1 -- Brendan Lawlor 06/17/2018 Reviewed

Reviewer_2_Report_(Original_Submission) -- Joshua W. K. Ho, PhD 05/04/2018 Reviewed

Reviewer_2_Report_Revision_1 -- Joshua W. K. Ho, PhD 06/16/2018 Reviewed

Reviewer_2_Report_Revision_2 -- Joshua W. K. Ho, PhD 07/08/2018 Reviewed

Reviewer_3_Report_(Original_Submission) -- Tae-Hyuk Ahn 05/05/2018 Reviewed

Reviewer_3_Report_Revision_1 -- Tae-Hyuk Ahn 6/23/2018 Reviewed

Supplemental Files

## Abbreviations

API: application programming interface; AWS: Amazon Web Services; BLAST: Basic Local Alignment Search Tool; BWA: Burrows–Wheeler aligner; CPU: central processing unit; DAG: directed acyclic graph; GATK: Genome Analysis Toolkit; HDFS: Hadoop Distributed File System; I/O: input output system; MPI: message passing interface; MSA: multiple sequence alignment; NCBI: National Center for Biotechnology Information; NGS: next-generation sequencing; OLC: overlap–layout–consensus; RDD: resilient distributed dataset; scRNA-seq: single-cell RNA sequencing; SIMD: single instruction multiple data; SW: Smith-Waterman algorithm.
