# Supplementary material for: Bioinformatics applications on Apache Spark
Source: Gigascience. 2018 Aug 7;7(8):giy098. doi: 10.1093/gigascience/giy098 (PMC6113509; doi:10.1093/gigascience/giy098)
Supplement: GIGA-D-18-00131_Original_Submission.pdf [file giy098_giga-d-18-00131_original_submission.pdf]

GigaScience

Bioinformatics Application on Apache Spark

--Manuscript Draft--

**Manuscript Number:**	GIGA-D-18-00131
**Full Title:**	Bioinformatics Application on Apache Spark
**Article Type:**	Review
**Funding Information:**	National Key R&D Program of China (2017YFB0202600, 2016YFC1302500, 2016YFB0200400 and 2017YFB0202104) — Professor shaoliang peng<br>National Natural Science Foundation of China (61772543, U1435222, 61625202, 61272056 and 61771331) — Professor shaoliang peng<br>Guangdong Provincial Department of Science and Technology (2016B090918122) — Professor shaoliang peng
**Abstract:**	With the rapid development of the next-generation sequencing (NGS), the ever-increasing genomic data poses a tremendous challenge to data processing. Therefore, there is an urgent need for highly scalable and powerful computational systems. Among the state-of–the-art parallel computing platforms, Apache Spark is a fast, general-purpose computing framework designed for large-scale data processing, which ensures high fault-tolerance and high scalability by introducing resilient distributed dataset (RDD) abstraction. Moreover, Spark can be up to 100x faster in memory access and 10x faster in disk access than Hadoop. In this paper, we surveyed Spark-based applications in the NGS and other biological domains, such as phylogeny, drug discovery and more. In the end, we discussed the challenges faced in this field and the future work on parallel computing of bioinformatics. We believe that this survey provides a comprehensive guideline for bioinformatics researchers to apply Spark in their own fields.<br>Keywords: next-generation sequencing; bioinformatics; Apache Spark; resilient distributed dataset; memory computing
**Corresponding Author:**	runxin guo<br><br>CHINA
**Corresponding Author Secondary Information:**	
**Corresponding Author's Institution:**	
**Corresponding Author's Secondary Institution:**	
**First Author:**	runxin guo
**First Author Secondary Information:**	
**Order of Authors:**	runxin guo
	yi zhao
	xiangke liao
	kenli li
	quan zou
	xiaodong fang
	shaoliang peng
**Order of Authors Secondary Information:**	
**Additional Information:**	

Powered by Editorial Manager® and ProduXion Manager® from Aries Systems Corporation

Question	Response
Are you submitting this manuscript to a special series or article collection?	No
**Experimental design and statistics**<br><br>Full details of the experimental design and statistical methods used should be given in the Methods section, as detailed in our Minimum Standards Reporting Checklist. Information essential to interpreting the data presented should be made available in the figure legends.<br><br>Have you included all the information requested in your manuscript?	Yes
**Resources**<br><br>A description of all resources used, including antibodies, cell lines, animals and software tools, with enough information to allow them to be uniquely identified, should be included in the Methods section. Authors are strongly encouraged to cite Research Resource Identifiers (RRIDs) for antibodies, model organisms and tools, where possible.<br><br>Have you included the information requested as detailed in our Minimum Standards Reporting Checklist?	Yes
**Availability of data and materials**<br><br>All datasets and code on which the conclusions of the paper rely must be either included in your submission or deposited in publicly available repositories (where available and ethically appropriate), referencing such data using a unique identifier in the references and in the "Availability of Data and Materials" section of your manuscript.<br><br>Have you have met the above requirement as detailed in our Minimum Standards Reporting Checklist?	Yes

Manuscript
Click here to download Manuscript Bioinformatics Application on Apache Spark.docx

Bioinformatics Application on Apache Spark

Runxin GUO[1†], Yi ZHAO[4†], Xiangke LIAO[3], Kenli LI[2], Quan ZOU[5*], Xiaodong FANG[6*], Shaoliang PENG[2,3†*]

[1]College of Meteorology and Oceanology, National University of Defense Technology, Changsha 410073, China

[2]College of Computer Science and Electronic Engineering & National Supercomputer Centre in Changsha, Hunan University, Changsha 410082, China

[3]College of Computer, National University of Defense Technology, Changsha 410073, China

[4]Institute of Computing Technology, Chinese Academy of Sciences, Beijing 100190, China

[5]School of Computer Science and Technology, Tianjin University, Tianjin 300350, China

[6]BGI Genomics, BGI-Shenzhen, Shenzhen 518083, China

pengshaoliang@nudt.edu.cn ; zouquan@nclab.net ; fangxd@bgitechsolutions.com

*: corresponding author; †: equal contributors

ABSTRACT

With the rapid development of the next-generation sequencing (NGS), the ever-increasing genomic data poses a tremendous challenge to data processing. Therefore, there is an urgent need for highly scalable and powerful computational systems. Among the state-of–the-art parallel computing platforms, Apache Spark is a fast, general-purpose computing framework designed for large-scale data processing, which ensures high fault-tolerance and high scalability by introducing resilient distributed dataset (RDD) abstraction. Moreover, Spark can be up to 100x faster in memory access and 10x faster in disk access than Hadoop. In this paper, we surveyed Spark-based applications in the NGS and other biological domains, such as phylogeny, drug discovery and more. In the end, we discussed the challenges faced in this field and the future work on parallel computing of bioinformatics. We believe that this survey provides a comprehensive guideline for bioinformatics researchers to apply Spark in their own fields.

Keywords: next-generation sequencing; bioinformatics; Apache Spark; resilient distributed dataset; memory computing

INTRODUCTION

NGS has generated huge amounts of biological sequence data. To use these data efficiently, we need to store and analyze the data accurately and efficiently. However, the existing bioinformatics tools cannot effectively handle such a large amount of data. In order to solve the issues, MapReduce, a programming model for parallel computation of large datasets, has been proposed [1]. MapReduce splits large-scale datasets into many key-value pairs through both the map and reduce phases,

significantly improving performance and showing good scalability. Hadoop consists of two parts: the Hadoop Distributed File System (HDFS) and MapReduce, where HDFS is mainly used for distributed storage of massive datasets and MapReduce preforms distributed computing on these datasets. Hadoop is a software framework that enables distributed processing of large amounts data in a reliable, efficient, and scalable way. As a result, Hadoop has been adopted by the bioinformatics community in several areas [2], such as alignment [3], mapping [4] and sequence analysis [5].

However, as in Figure 1, due to its disk-based I/O access pattern, intermediate calculation results are also stored in HDFS. Therefore, Hadoop is only suitable for batch data processing, not enough for interactive and real-time data processing, and shows poor performance for iterative data processing. To resolve this problem, Apache Spark [6] has been proposed, which is a fast general-purpose computational framework designed specifically to handle huge amounts of data. Unlike Hadoop's disk-based computing, Spark performs memory computing by introducing resilient distributed dataset (RDD). RDD is a read-only and fault-tolerant data structure. These useful differences make Spark even better for some workloads. In other words, in addition to providing interactive queries, Spark also supports in-memory distributed datasets and optimizes iterative workloads. Moreover, Spark can be up to 100x faster in memory access than Hadoop [6]. Even if we compare between them based on the performance of the disk, the gap is more than 10 times [7].

THE SPARK FRAMEWORK

Spark is an open source cluster computing environment similar to Hadoop, developed by UC Berkeley AMP lab. As in Figure 2, Spark architecture consists of three main components: (a) the driver program, used to deploy the Spark operating environment and launch computation; (b) the cluster manager, responsible for obtaining and allocating the computing resources; (c) the worker nodes, in charge of performing real computations. It is implemented in the Scala language and uses Scala as its application framework. Unlike Hadoop, Spark and Scala are tightly integrated, with Scala operating distributed datasets just as easily as local collection objects. Moreover, Spark has the benefits of Hadoop MapReduce, but unlike Hadoop MapReduce, intermediate calculation results can be stored in memory, eliminating the need to read and write HDFS. So, Spark is better suited for iterative algorithms such as data mining and machine learning. Besides, Spark adopts directed acyclic graph (DAG) to optimize the execution process.

Spark implements in-memory operations based on the RDD abstraction. RDD is a read-only collection of objects partitioned on different nodes in a cluster so that the data in the RDD can be processed in parallel. The most important feature of RDD is that it provides fault tolerance and can automatically recover from a node failure. That is, if an RDD partition on a node is lost because of

a node failure, the RDD automatically recalculates the partition from its own data source. All this is transparent to the user. In addition, RDD data is stored in memory by default, but Spark automatically writes RDD data to disk if memory resources are low. Spark provides two types of operations on RDDs: transformations and actions. The former defines a new RDD, the latter returns a result or writes RDD data to the storage system. Table 1 lists the commonly-used transformations and actions supported by Spark. Transformations employ lazy operations [8], which means that the operation of generating another RDD from one RDD transformation is not executed immediately, and the calculation process is not actually started until an action is performed. Figure 3 shows Spark's task processing flow chart.

Besides, as in Figure 4, the Spark ecosystem, the BDAS (Berkeley Data Analysis Stack), includes components such as Spark SQL, Spark Streaming, MLlib, and GraphX. These components provide the real-time processing applications for Spark Streaming, the ad hoc query for Spark SQL, the machine learning for MLlib, and the GraphX graph processing.

SPARK IN ALIGNMENT AND MAPPING

The rapid development of NGS technology has generated a large amount of sequence data (reads), which has a tremendous impact on sequence alignment and mapping process. Currently, the sequence alignment and mapping process still consume a lot of time.

The Smith-Waterman (SW) algorithm [9], which produces the optimal local alignment between two strings of nucleic acid sequences or protein sequences, is widely used in bioinformatics. However, SW algorithm requires a high computational cost due to high computational complexity. To speed up the algorithm, in 2015, Zhao G *et al* implemented the SW algorithm on Spark for the first time, called as SparkSW [10]. It consisted of three phases: data preprocessing, SW as map tasks and top K records as reduce tasks. Experimental results [10] showed that SparkSW was load-balancing and scalable with computing resources increased.

However, SparkSW merely supports SW algorithm without the mapping location and traceback of optimal alignment, as a result, SparkSW executes slowly. Therefore, in 2017, Xu Bo *et al* proposed DSA [11], which employed Single Instruction Multiple Data (SIMD) instruction to parallel the sequence alignment algorithm at each worker node. Experimental results [11] showed that DSA achieved up to 201x speedup over SparkSW and almost linear speedup with the increase of cluster nodes.

Subsequently, Xu Bo *et al* proposed CloudSW [12], an efficient distributed SW algorithm which leveraged Spark and SIMD instructions to accelerate the algorithm and provided APIs service in the cloud. Experimental results [12] showed that CloudSW achieved up to 3.29x speedup over DSA

and 621x speedup over SparkSW. And CloudSW also showed excellent scalability and achieved up to 529 giga cell updates per second (GCUPS) in protein database search with 50 nodes in Aliyun.

Burrows-Wheeler aligner (BWA) is composed of BWA-backtrack [13], BWA-SW [14] and BWA-MEM [15] for performing sequence alignment and mapping in bioinformatics. Before the advent of Spark-based BWA tool, there were several other BWA tools based on big data technology, including BigBWA [16], Halvade [17] and SEAL [18]. However, they were based on Hadoop showing limited scalability and complex implementation.

As a result, in 2015, Al-Ars Zaid *et al* [19] implemented three different versions of BWA-MEM and compared their performance: a native cluster-based version, a Hadoop version and a Spark version. Three implementations were evaluated on the same IBM Power7 and Intel Xeon servers with the WordCount example. Results [19] showed that simultaneous multithreading improved the performance of three versions of BWA-MEM, and the Spark version with 80 threads increased performance by up to 87% than the native cluster version using 16 threads. Furthermore, the Hadoop version with 4 threads increased performance by 17% and the Spark version with more threads increased performance by 27%.

After then, in 2016, Abuín JM *et al* proposed SparkBWA [20] which is composed of three main phases: the RDDs creation phase, the map phase, and the reduce phase. Experimental results [20] showed that for the BWA-backtrack algorithm, SparkBWA achieved the average speedup of 1.9x and 1.4x compared with SEAL and pBWA respectively. For the BWA-MEM algorithm, SparkBWA was 1.4x faster than BigBWA and Halvade tools on average.

However, SparkBWA required the data availability in the HDFS. In general, the input files were given in gzip format, which required first uncompressing the file before uploading it to the HDFS. Subsequently, this also slowed down the execution of BWA itself, since data on the HDFS had to be reformatted as appropriate input to the BWA program tasks running on the cluster. Finally, the output files produced by those BWA tasks required significant time to combine separately at the end.

Therefore, in 2017, Alars HMA *et al* employed Spark to propose StreamBWA [21], where the input data was being streamed directly from a compressed file. This file could either be located on the master node or on a URL, which eliminated the cost of execution time of downloading the file and then uncompressing it. Moreover, since the master node could stream data to the data nodes, the overhead of uploading data to the HDFS could also be hidden. The master node could also start combining the output files of BWA tasks running on the data nodes, in parallel, once they were available, further reducing the overall time. Experimental results [21] showed that this streaming

distributed approach was approximately 2x faster than the non-streaming approach. Furthermore, StreamBWA was 5x faster than SparkBWA.

Multiple sequence alignment (MSA) refers to the sequence alignment of three or more biological sequences, such as protein or nucleic acid sequences. One of representative tools for performing MSA is PASTA [22]. PASTA is a derivative of SATé [23], which produces highly accurate alignments in shared memory computers. However, PASTA is limited to processing small and medium datasets, because the computing power of shared memory systems cannot meet the memory and time requirements of large-scale datasets.

Therefore, in 2017, Abuín J M *et al* proposed PASTASpark [24], which allowed executions on a distributed memory cluster taking advantage of Spark. It employed an in-memory RDD of key-value pairs to parallel the calculating MSAs phase. Experiments were conducted on two different clusters (CESGA and AWS). The results [24] showed that PASTASpark achieved up to 10x speedups compared with single-threaded PASTA and was able to process 200,000 sequences in 24 hours using only AWS nodes. Therefore, PASTASpark ensured scalability and fault tolerance which greatly reduced the time to obtain MSA.

The probabilistic pairwise model [25] is widely used in all consistency-based MSA tools, such as MAFFT [26], ProbCons [27] and T-Coffee(TC) [28]. However, the global distributed memory cannot meet the ever-increasing sequence datasets, which causes the need of specialized distributed databases, such as HBase or Cassandra. As a result, in 2017, Lladós Jordi *et al* employed Spark to propose a new tool, PPCAS [29], which could parallel the probabilistic pairwise model for large-scale protein sequences and store it in a distributed platform. Experimental results [29] showed that it was better with single node and provided almost linear speedup with the increase in the number of nodes. In addition, it could compute more sequences using the same memory.

NCBI BLAST [30, 31] is widely used to implement algorithms for sequence comparison. Before the Spark-based BLAST was created, several other BLAST tools had been proposed including mpiBLAST [32], GPU-BLAST [33] and CloudBLAST [34]. However, with the increasing number of genomic data, these tools showed limited scalability and efficiency.

As a result, in 2017, Castro MRD *et al* proposed SparkBLAST [35], which utilized cloud computing and Spark framework to parallel BLAST. In SparkBLAST, Spark's *pipe* operator and RDDs were utilized to call BLAST as an external library and perform scalable sequence alignment. It was compared with CloudBLAST on both Google and Microsoft Azure Clouds. Experimental results [35] showed that SparkBLAST outperformed CloudBLAST in terms of speedup, scalability and efficiency.

Metagenomics is crucial for studying genetic material directly from environmental samples. Fragment recruitment is the process of aligning reads to reference genomes in metagenomics data analysis. And in 2017, Zhou W *et al* proposed MetaSpark [36], which employed Spark to recruit metagenomics reads to reference genomes.

MetaSpark utilized the RDD of Spark to cache datasets in memory and scaled well along dataset size increments. It consisted of five steps including constructing k-mer RefindexRDD, constructing k-mer ReadlistRDD, seeding, filtering, and banded alignment. It was evaluated on a ten-node cluster working under the Spark standalone module where each node contained an 8-core CPU and 16 GB RAM. It employed about one million 75bp Illumina reads dataset and two references (the 194 human gut genomes and the bacterial genomes) that were respectively 0.616GB and 1.3GB in size. Experimental results [36] showed that MetaSpark recruited more reads than FR-HIT [37] with the same parameters and 1 million reads. MetaSpark recruited 501,856 reads when there were 0.616 GB human gut genome references, while FR-HIT recruited 489,638 reads. MetaSpark increased recruited reads by 2.5%. When references changed to a 1.3 GB bacterial genome, MetaSpark recruited 463,862 reads, while FR-HIT recruited 444,671 reads. MetaSpark increased recruited reads by 4%. Moreover, the results also showed that MetaSpark offered good scalability. Under a 0.616 GB reference, run time for 0.1 million reads was 51 min under 4 nodes, and decreased slightly to 23.5 min under 10 nodes. For the 1 million read datasets, MetaSpark would crash under 4 nodes due to limited memory. Under 6 nodes, it finished running after 312 min and would sharply decrease to 201 min under 10 nodes.

SPARK IN ASSEMBLY

Due to short lengths of the NGS reads (<500 bp), they need to be assembled prior to further analysis, which is another important phase in sequence analysis workflow. In general, there are two types of assembly: the reference assembly and *de novo* assembly. The assembly algorithm includes two categories: overlap-layout-consensus (OLC) algorithm and the de Bruijn graph algorithm. The former is generally employed to assemble longer reads, while the latter shows a good performance in assembling short reads.

Before Spark-based distributed memory de novo assemblers were created, although there were some MPI-based assemblers (such as Ray [38], AbySS [39] and SWAP-Assembler [40]), they showed limited scalability, accuracy, and computational efficiency. Therefore, in 2015, Abu-Doleh Anas *et al* proposed Spaler [41] taking advantage of Spark and GraphX API. It consisted of two main parts: (a) de Bruijn graph construction, and (b) Contigs generating. And it was evaluated with other MPI-

based tools in terms of quality, execution time, and scalability. Experiments results [41] showed that Spaler had better scalability and it could achieve comparable or better assemble quality.

And in 2016, X Pan *et al* [42] put forward a new assembling algorithm based on Spark which employed the method of matching K-2 bit to simplify the de Bruijn graph. This algorithm was evaluated using 6 groups of DNA in the NCBI. Experimental results [42] showed that this strategy not only solved the problem of low efficiency based on the MapReduce algorithm, but also optimized the algorithm itself. The combination of these two aspects were very suitable for the large-scale DNA sequence assembling. Besides, results also showed that the new sequence assembling algorithm based on Spark could ensure accuracy of assembling results.

To address the problem of poor assembling precision and low efficiency, in 2017, Dong G *et al* [43] proposed SA-BR-Spark, a new sequence assembly algorithm based on Spark. The authors first designed a precise assembling algorithm under the strategy of finding the source of reads based on the MapReduce and Eulerian path algorithm (SA-BR-MR). SA-BR-MR calculated 54 sequences which were randomly selected from animals, plants and microorganisms with base lengths from hundreds to tens of thousands from NCBI. All matching rates of 54 sequences were 100%. For each species, the algorithm also summarized the range of K which made the matching rates to be 100%. In order to verify the range of K value of hepatitis C virus (HCV) and related variants, the randomly selected eight HCV variants were calculated. The results confirmed the correctness of K range of hepatitis C and related variants from NCBI. After that, SA-BR-Spark was put forward. Experimental results [43] showed that SA-BR-Spark provided a superior computational speed compared with SA-BR-MR.

To resolve the large memory requirement problem of most OLC *de novo* assemblers, in 2017, Paul AJ *et al* [44] employed string graph reduction algorithms taking advantage of Spark. The proposed Spark algorithms were evaluated with a very large sample dataset. Results showed that this dataset was assembled by the proposed Spark algorithms using 15 virtual machines in 0.5 hours compared to the 7.5 hours of OLC based Omega [45] assembler.

SPARK IN SEQUENCE ANALYSIS

Spark in variant analysis

The GATK (Genome Analysis Toolkit) DNA analysis pipeline is widely used in genomic data analysis. Before Spark-based GATK tools were created, while several other tools had been developed to address the issue of scalability in the pipeline (such as Halvade [17] and Churchill [46]), they showed limited scalability, accuracy and computational efficiency.

Therefore, in 2015, Mushtaq H *et al* [47] utilized Spark to propose a cluster-based GATK pipeline. To reduce the execution time, this approach kept data active in the memory between the map and reduce phases. By using runtime statistics of the active workload, it achieved a dynamic load balancing algorithm that could better utilize system performance. Experimental results [47] showed that this method achieved a 4.5x speedup compared to the multi-threaded GATK pipeline on a single node. Besides, when executed on a 4-node cluster, this approach was 63% faster than Halvade.

After that, in 2016, Deng L *et al* proposed HiGene [48], which employed Spark to enable multi-core and multi-node parallelization of the GATK pipeline. HiGene put forward a dynamic computing resource scheduler and an efficient data skew mitigation method to improve performance. Experiments were conducted with the NA12878 whole human genome dataset. Results [48] showed that HiGene reduced the total running time from days to nearly an hour. Besides, compared with Halvade, HiGene was also 2x faster. Meanwhile, Li X *et al* employed Spark to propose GATK-Spark [49] to parallel the GATK pipeline by taking full account of compute, workload and I/O characteristics. And it was built on top of ADAM format [50]. Experimental results [49] showed that GATK-Spark shortened the total running time from 20 hours to 30 minutes on 256 CPU cores which achieved more than 37 times speedup.

The advent of Spark provides the possibility of interactive processing for NGS data. And in 2014, Wiewiórka MS *et al* proposed SparkSeq [51] to build and run genomic analysis pipelines in an interactive way by using Spark. Experimental results showed that SparkSeq achieved 8.4–9.15 times speedup than SeqPig. Besides, it could accelerate data querying up to 110x and reduce memory consumption by 13x.

Spark in motif analysis

Due to the nature of NGS technology, the generated data are usually accompanied by some noises or other types of errors which are known as uncertain data [52]. And among these uncertain data, there are some frequently recurring patterns called motifs [53]. Mining motifs from these uncertain data is an important problem but a computationally intensive task. Before Spark-based mining algorithm was created, while several mining algorithms had been developed (such as HPSPM [54], DGSP [55] and SPAMC [56]), they showed limited scalability. Therefore, Jiang F *et al* [57] utilized Spark to propose a scalable algorithm for mining sequence motifs. This algorithm took advantage of Spark's RDDs and DAG, and allowed users to specify the minimum and maximum length of motif. Experiments were conducted with human genome datasets and bacteria DNA sequence datasets and results [57] showed this approach could take a short period of time to extract accurate motifs.

SPARK IN OTHER BIOLOGICAL APPLICATIONS

Spark in genomic inference

Efficient score statistic methods [58] are widely applied in high-throughput genomic data inference. A typical method of estimating the sampling distribution of the statistics is to employ asymptotic approximation, but it is inappropriate for small or uncommon variants. Although resampling methods [59] are appropriate for genomic inference, they greatly increase the computational burden of analysis. In order to tackle the computational challenge for resampling based inference, Bahmani Amir *et al* proposed SparkScore [60], a distributed genomic inference approach taking advantage of Spark. SparkScore leveraged the nature of asymptotic and resampling inference based on efficient score statistics for distributed genomic inference. Experiments on synthetic datasets using Amazon Elastic MapReduce (EMR) [60] demonstrated SparkScore's efficiency and scalability.

Spark in epigenetics

CpG islands (CGI) are important epigenetic markers, which play an essential role in epigenetics [61]. However, it is very challenging to investigate the CpG islands and their structures. Before Spark-based applications were developed, while several methods had been proposed to determine the CPG island (such as bisulfite modification-based methods), they were time-consuming and too costly. Thus, Yu N *et al* [62] utilized Spark to propose a novel CpG box model and a Markov model to redefine and investigate the CpG island which could greatly accelerate the analytic process. Experiments were conducted with Human and mouse chromosome sequences, 24 chromosomes and 21 chromosomes. Results [62] showed this cloud-assisted method displayed considerable accuracy and faster processing power (6-7 times faster with 10 cores) compared with sequential processing.

Spark in phylogeny

Phylogeny reconstruction plays an important role in molecular evolutionary studies but faces significant computational challenges. Before Spark-based tools were created, while several tools had been put forward for phylogeny reconstruction, they could not scale well with a significant increase in data sets. Therefore, in 2016, Xu X *et al* proposed CloudPhylo [63], a fast and scalable Phylogeny reconstruction tool making use of Spark. It evenly distributed the entire computational workload among the working nodes. Experiment was conducted with the 5220 bacteria whole genome DNA sequences. Results [63] showed that CloudPhylo took 24508 seconds with one worker node and it could scale well as worker nodes increased. Moreover, CloudPhylo performed better than several existing tools when using more worker nodes. Besides, CloudPhylo achieved higher speedup on a larger dataset of about 100GB generated by simulation.

Spark in drug discovery

It is crucial to identify candidate molecules that affect disease-related proteins in drug discovery. Although the Chemogenomics project tries to identify candidate molecules using machine learning predictor programs [64-66], these programs spend a significant time and cannot be easily extended to multiple nodes. To migrate existing programs to multi-node clusters without changing the original programs, Harnie D *et al* proposed S-CHEMO [67] using Saprk. In S-CHEMO, the intermediate data would be consumed again immediately on nodes that generated the data, reducing time and network bandwidth consumption. Experiments [67] compared S-CHEMO with the original pipeline, which showed almost linear speedup up to 8 nodes. Besides, this implementation also allowed easier monitoring and checkpointing.

Spark in Single-cell RNA sequencing

Single-cell RNA sequencing (scRNA-seq) is crucial for understanding biological processes. Compared with standard bulk RNA-seq experiments, scRNA-seq experiments typically generate a greater number of cell profiles. Although there are already several RNA-seq processing pipelines (such as Halvade, SparkSeq and SparkBWA), they cannot process such a large number of profiles. Therefore, Falco [68] was created to process large-scale transcriptomic data in parallel by using Hadoop and Spark. Experiments were conducted with two public scRNA-seq datasets. Results [68] showed compared with a highly optimized single-node analysis, Falco was at least 2.6 times faster. Besides, as the number of computing nodes increased, running time decreased. Besides, it allowed users to employ the low-cost spot instances of AWS which reduced the cost of analysis by 65%.

Spark in variant association and population genetics studies

Effectively analyzing thousands of individuals and millions of variants is a computationally intensive problem. Traditional parallel strategies such as MPI/OpenMP show poor scalability. While Hadoop provides an efficient and scalable computing framework, it is heavily dependent on disk operations. Therefore, in 2015, O'Brien AR *et al* proposed VariantSpark [69] to parallel population-scale tasks based on Spark and associated machine learning library, MLlib. Experiments were conducted on 3000 individuals with 80 million variants, which showed that VariantSpark was 80% faster than ADAM, Hadoop/Mahout implementation and ADMIXTURE [70]. Besides, compared with R and Python implementations it was more than 90 % faster. And in 2017, Di Z *et al* proposed SEQSpark [71] to perform rare variant association analysis by using Spark. It was evaluated with whole-genome and simulated exome sequence data. The former was completed in 1.5 hours and the latter in 1.75 hours. Moreover, it was always faster than Variant Association Tools and PLINK/SEQ, and in some cases running time was reduced to one percent.

Spark in other works

Biological simulations and experiments produce a large number of numerical datasets, and in 2017 Klein M *et al* proposed Biospark [72] to process these data. Biospark was based on Hadoop and Spark, consisting of a set of Java, C++ and Python libraries. Besides, it provided the abstractions for parallel analysis of standard data types, including multidimensional arrays and images. To help parallel analysis of some common datasets, it also provided APIs and file conversion tools, including Monte Carlo, molecular dynamics simulations and time-lapse microscopy.

Table 2 summarizes bioinformatics tools and algorithms based on Apache Spark.

CONCLUSION

In conclusion, the Apache Spark is very suitable for processing large-scale datasets, due to its high performance, scalability and fault tolerance. With the rapid development of NGS technology, a large number of bioinformatics data have been generated, which poses a great challenge to traditional bioinformatics tools. For this reason, we have summarized the relevant works about Spark in bioinformatics and made a guideline on this topic. First, we make a comparison between Spark and Hadoop, and then introduce the Spark architecture, programming model, and processing mechanism in detail. After that, we survey Spark-based applications in the NGS and other biological domains. A researcher who wants to get involved in this field can have a general understanding of Spark in bioinformatics through our survey. Currently, Spark has been widely used in the field of bioinformatics and shows good results. We believe that bioinformatics applications based on Spark will provide promising performance for biological researchers in the future.

Key Points

- The Apache Spark not only gives researchers a possibility of achieving efficient, scalable and fault tolerant computing performance, but also supports various system workloads such as batch processing, iterative, interactive and flow calculations.
- We introduce the Apache Spark framework in detail, helping researchers to understand its architecture, programming model and processing mechanism.
- We present Spark-based applications that can be employed in bioinformatics and discuss the future of parallel computing in bioinformatics.

COMPETING INTERESTS

The authors declare that they have no competing interests.

FUNDING

This work was supported by National Key R&D Program of China [grant numbers 2017YFB0202600, 2016YFC1302500, 2016YFB0200400 and 2017YFB0202104]; National

Natural Science Foundation of China [grant numbers 61772543, U1435222, 61625202, 61272056 and 61771331]; and Guangdong Provincial Department of Science and Technology [grant number 2016B090918122].

REFERENCES

1. Dean J, Ghemawat S. **MapReduce: simplified data processing on large clusters**. *Communications of the ACM* 2008, **51**(1):107-113.
2. Zou Q, Li X-B, Jiang W-R *et al*. **Survey of MapReduce frame operation in bioinformatics**. *Briefings in bioinformatics* 2013, **15**(4):637-647.
3. Zou Q, Hu Q, Guo M *et al*. **HAlign: Fast multiple similar DNA/RNA sequence alignment based on the centre star strategy**. *Bioinformatics* 2015, **31**(15):2475-2481.
4. Nguyen T, Shi W, Ruden D. **CloudAligner: A fast and full-featured MapReduce based tool for sequence mapping**. *BMC research notes* 2011, **4**(1):171.
5. Nordberg H, Bhatia K, Wang K *et al*. **BioPig: a Hadoop-based analytic toolkit for large-scale sequence data**. *Bioinformatics* 2013, **29**(23):3014-3019.
6. Zaharia M, Chowdhury M, Franklin MJ *et al.* **Spark: Cluster computing with working sets**. *HotCloud* 2010, **10**(10-10):95.
7. Han Z, Zhang Y. **Spark: A Big Data Processing Platform Based on Memory Computing**. In: *Seventh International Symposium on Parallel Architectures, Algorithms and Programming: 2016*. 172-176.
8. Zaharia M, Chowdhury M, Das T *et al*. **Resilient distributed datasets: A fault-tolerant abstraction for in-memory cluster computing**. In: *Proceedings of the 9th USENIX conference on Networked Systems Design and Implementation: 2012*. USENIX Association: 2-2.
9. Smith TF, Waterman MS. **Identification of common molecular subsequences**. *Journal of Molecular Biology* 1981, **147**(1):195-197.
10. Zhao G, Ling C, Sun D. **SparkSW: Scalable Distributed Computing System for Large-Scale Biological Sequence Alignment**. In: *Ieee/acm International Symposium on Cluster, Cloud and Grid Computing: 2015*. 845-852.
11. Xu B, Li C, Zhuang H *et al*. **DSA: Scalable Distributed Sequence Alignment System Using SIMD Instructions**. In: *Ieee/acm International Symposium on Cluster, Cloud and Grid Computing: 2017*. 758-761.
12. Xu B, Li C, Zhuang H *et al*. **Efficient Distributed Smith-Waterman Algorithm Based on Apache Spark**. In: *IEEE International Conference on Cloud Computing: 2017*. 608-615.

13. Li H, Durbin R. **Fast and accurate short read alignment with Burrows–Wheeler transform**: Oxford University Press; 2009.

14. Li H, Durbin R. **Fast and accurate long-read alignment with Burrows–Wheeler transform**. *Bioinformatics* 2010, **26**(5):589-595.

15. Li H. **Aligning sequence reads, clone sequences and assembly contigs with BWA-MEM**. 2013, **1303**.

16. Abuín JM, Pichel JC, Pena TF *et al*. **BigBWA: approaching the Burrows–Wheeler aligner to Big Data technologies**. *Bioinformatics* 2015, **31**(24):4003.

17. Decap D, Reumers J, Herzeel C *et al*. **Halvade: scalable sequence analysis with MapReduce**. *Bioinformatics* 2015, **31**(15):2482-2488.

18. Pireddu L, Leo S, Zanetti G. **SEAL: a distributed short read mapping and duplicate removal tool**. *Bioinformatics* 2011, **27**(15):2159.

19. Al-Ars Z, Mushtaq H. **Scalability Potential of BWA DNA Mapping Algorithm on Apache Spark**. In: *SIMBig: 2015*. 85-88.

20. Abuín JM, Pichel JC, Pena TF *et al*. **SparkBWA: Speeding Up the Alignment of High-Throughput DNA Sequencing Data**. *Plos One* 2016, **11**(5):e0155461.

21. Alars HMA. **Streaming Distributed DNA Sequence Alignment Using Apache Spark**. 2017.

22. Mirarab S, Nguyen N, Warnow T. **PASTA: ultra-large multiple sequence alignment**. In: *International Conference on Research in Computational Molecular Biology: 2014*. Springer: 177-191.

23. Liu K, Warnow TJ, Holder MT *et al*. **SATe-II: very fast and accurate simultaneous estimation of multiple sequence alignments and phylogenetic trees**. *Systematic biology* 2011, **61**(1):90-106.

24. Abuín JM, Pena TF, Pichel JC. **PASTASpark: multiple sequence alignment meets Big Data**. *Bioinformatics* 2017, **33**(18):2948-2950.

25. Miyazawa S. **A reliable sequence alignment method based on probabilities of residue correspondences**. *Protein Engineering* 1995, **8**(10):999.

26. Katoh K, Standley DM. **MAFFT Multiple Sequence Alignment Software Version 7: Improvements in Performance and Usability**. *Molecular Biology & Evolution* 2013, **30**(4):772-780.

27. Do CB, Mahabhashyam MS, Brudno M *et al*. **ProbCons: Probabilistic consistency-based multiple sequence alignment**. *Genome Research* 2005, **15**(2):330.

28. Tommaso PD, Moretti S, Xenarios I *et al*. **T-Coffee: a web server for the multiple sequence alignment of protein and RNA sequences using structural information and homology extension**. *Nucleic Acids Research* 2011, **39**(Web Server issue):13-17.

29. Lladós J, Guirado F, Cores F *et al*. **PPCAS: Implementation of a Probabilistic Pairwise Model for Consistency-Based Multiple Alignment in Apache Spark**; 2017.

30. Altschul S, Gish W, Miller W *et al*. **Basic local alignment search tool. J. Mol. Biol**. 1990.

31. C C, G C, V A *et al*. **BLAST+: architecture and applications**. *Bmc Bioinformatics* 2009, **10**(1):421.

32. Darling AE, Carey L, Feng WC. **The design, implementation, and evaluation of mpiBLAST**. In.: Los Alamos National Laboratory; 2003.

33. Vouzis PD, Sahinidis NV. **GPU-BLAST: using graphics processors to accelerate protein sequence alignment**. *Bioinformatics* 2010, **27**(2):182-188.

34. Matsunaga A, Tsugawa M, Fortes J. **Cloudblast: Combining mapreduce and virtualization on distributed resources for bioinformatics applications**. In: *eScience, 2008 eScience'08 IEEE Fourth International Conference on: 2008*. IEEE: 222-229.

35. Castro MRD, Tostes CDS, Dávila AMR *et al*. **SparkBLAST: scalable BLAST processing using in-memory operations**. *Bmc Bioinformatics* 2017, **18**(1):318.

36. Zhou W, Li R, Yuan S *et al*. **MetaSpark: a spark-based distributed processing tool to recruit metagenomic reads to reference genomes**. *Bioinformatics* 2017, **33**(7):1090-1092.

37. Niu B, Zhu Z, Fu L *et al*. **FR-HIT, a very fast program to recruit metagenomic reads to homologous reference genomes**. *Bioinformatics* 2011, **27**(12):1704-1705.

38. Boisvert S, Laviolette F, Corbeil J. **Ray: simultaneous assembly of reads from a mix of high-throughput sequencing technologies**. *Journal of Computational Biology A Journal of Computational Molecular Cell Biology* 2010, **17**(11):1519.

39. Simpson JT, Wong K, Jackman SD *et al*. **ABySS: a parallel assembler for short read sequence data**. *Genome Research* 2009, **19**(6):1117.

40. Meng J, Wang B, Wei Y *et al*. **SWAP-Assembler: scalable and efficient genome assembly towards thousands of cores**. *Bmc Bioinformatics* 2014, **15**(S9):S2.

41. Abu-Doleh A, Çatalyürek ÜV. **Spaler: Spark and GraphX based de novo genome assembler**. In: *IEEE International Conference on Big Data: 2015*. 1013-1018.

42. Pan X, Fu X-L, Dong G-F *et al*. **DNA sequence splicing algorithm based on Spark**. In: *Industrial Informatics-Computing Technology, Intelligent Technology, Industrial Information Integration (ICIICII), 2016 International Conference on: 2016*. IEEE: 52-56.

43. Dong G, Fu X, Li H *et al*. **An Accurate Sequence Assembly Algorithm for Livestock, Plants and Microorganism Based on Spark**. *International Journal of Pattern Recognition & Artificial Intelligence* 2017, **31**(8).

44. Paul AJ, Lawrence D, Ahn TH. **Overlap Graph Reduction for Genome Assembly using Apache Spark**. In: *The ACM International Conference: 2017*. 613-613.

45. Haider B, Ahn TH, Bushnell B *et al*. **Omega: an Overlap-graph de novo Assembler for Metagenomics**. *Bioinformatics* 2014, **30**(19):2717-2722.

46. Kelly BJ, Fitch JR, Hu Y *et al*. **Churchill: an ultra-fast, deterministic, highly scalable and balanced parallelization strategy for the discovery of human genetic variation in clinical and population-scale genomics**. *Genome biology* 2015, **16**(1):6.

47. Mushtaq H, Al-Ars Z. **Cluster-based Apache Spark implementation of the GATK DNA analysis pipeline**. In: *Bioinformatics and Biomedicine (BIBM), 2015 IEEE International Conference on: 2015*. IEEE: 1471-1477.

48. Deng L, Huang G, Zhuang Y *et al*. **HiGene: A high-performance platform for genomic data analysis**. In: *IEEE International Conference on Bioinformatics and Biomedicine: 2016*. 576-583.

49. Li X, Tan G, Zhang C *et al*. **Accelerating large-scale genomic analysis with Spark**. In: *Bioinformatics and Biomedicine (BIBM), 2016 IEEE International Conference on: 2016*. IEEE: 747-751.

50. Massie M, Nothaft F, Hartl C *et al*. **Adam: Genomics formats and processing patterns for cloud scale computing**. *EECS Department, University of California, Berkeley, Tech Rep UCB/EECS-2013-207* 2013.

51. Wiewiórka MS, Messina A, Pacholewska A *et al*. **SparkSeq: fast, scalable and cloud-ready tool for the interactive genomic data analysis with nucleotide precision**. *Bioinformatics* 2014, **30**(18):2652-2653.

52. Leung CK-S. **Uncertain frequent pattern mining**. In: *Frequent pattern mining*. Springer; 2014: 339-367.

53. Das MK, Dai H-K. **A survey of DNA motif finding algorithms**. *BMC bioinformatics* 2007, **8**(7):S21.

54. Shintani T, Kitsuregawa M. **Mining algorithms for sequential patterns in parallel: Hash based approach**. In: *Pacific-Asia Conference on Knowledge Discovery and Data Mining: 1998*. Springer: 283-294.

55. Qiao S, Tang C, Dai S *et al*. **Partspan: Parallel sequence mining of trajectory patterns**. In: *Fuzzy Systems and Knowledge Discovery, 2008 FSKD'08 Fifth International Conference on: 2008*. IEEE: 363-367.

56. Chen C-C, Tseng C-Y, Chen M-S. **Highly scalable sequential pattern mining based on mapreduce model on the cloud**. In: *Big Data (BigData Congress), 2013 IEEE International Congress on: 2013*. IEEE: 310-317.

57. Jiang F, Leung CK, Sarumi OA *et al*. **Mining sequential patterns from uncertain big DNA in the spark framework**. In: *Bioinformatics and Biomedicine (BIBM), 2016 IEEE International Conference on: 2016*. IEEE: 874-881.

58. Rao CR. **Large sample tests of statistical hypotheses concerning several parameters with applications to problems of estimation**. In: *Mathematical Proceedings of the Cambridge Philosophical Society: 1948*. Cambridge University Press: 50-57.

59. Westfall PH, Young SS. **Resampling-based multiple testing: Examples and methods for p-value adjustment**, vol. 279: John Wiley & Sons; 1993.

60. Bahmani A, Sibley AB, Parsian M *et al*. **SparkScore: leveraging apache spark for distributed genomic inference**. In: *Parallel and Distributed Processing Symposium Workshops, 2016 IEEE International: 2016*. IEEE: 435-442.

61. Erkek S, Hisano M, Liang C-Y *et al*. **Molecular determinants of nucleosome retention at CpG-rich sequences in mouse spermatozoa**. *Nature structural & molecular biology* 2013, **20**(7):868-875.

62. Yu N, Li B, Pan Y. **A cloud-assisted application over apache spark for investigating epigenetic markers on DNA genome sequences**. In: *Big Data and Cloud Computing (BDCloud), Social Computing and Networking (SocialCom), Sustainable Computing and Communications (SustainCom)(BDCloud-SocialCom-SustainCom), 2016 IEEE International Conferences on: 2016*. IEEE: 67-74.

63. Xu X, Ji Z, Zhang Z. **CloudPhylo: a fast and scalable tool for phylogeny reconstruction**. *Bioinformatics* 2016, **33**(3):438-440.

64. Wale N. **Machine learning in drug discovery and development**. *Drug Development Research* 2011, **72**(1):112-119.

65. Costello JC, Heiser LM, Georgii E *et al*. **A community effort to assess and improve drug sensitivity prediction algorithms**. *Nature biotechnology* 2014, **32**(12):1202-1212.

66. Sastry GM, Inakollu VS, Sherman W. **Boosting virtual screening enrichments with data fusion: coalescing hits from two-dimensional fingerprints, shape, and docking**. *Journal of chemical information and modeling* 2013, **53**(7):1531-1542.

67. Harnie D, Saey M, Vapirev AE *et al*. **Scaling machine learning for target prediction in drug discovery using apache spark**. *Future Generation Computer Systems* 2017, **67**:409-417.

68. Yang A, Troup M, Lin P *et al*. **Falco: a quick and flexible single-cell RNA-seq processing framework on the cloud**. *Bioinformatics* 2016, **33**(5):767-769.

69. O'Brien AR, Saunders NFW, Guo Y *et al*. **VariantSpark: population scale clustering of genotype information**. *Bmc Genomics* 2015, **16**(1):1-9.

70. Alexander DH, Novembre J, Lange K. **Fast model-based estimation of ancestry in unrelated individuals**. *Genome Research* 2009, **19**(9):1655.

71. Di Z, Zhao L, Li B *et al*. **SEQSpark: A Complete Analysis Tool for Large-Scale Rare Variant Association Studies Using Whole-Genome and Exome Sequence Data**. *American Journal of Human Genetics* 2017, **101**(1):115.

72. Klein M, Sharma R, Bohrer CH *et al*. **Biospark: scalable analysis of large numerical datasets from biological simulations and experiments using Hadoop and Spark**. *Bioinformatics* 2017, **33**(2):303-305.

Table List

Table 1 Commonly-used transformations and actions on RDDs in Apache Spark

Table 2 Apache Spark-based bioinformatics tools and algorithms

Table 1 Commonly-used transformations and actions on RDDs in Apache Spark

**Transformations**	map (*f*): Apply the function *f* to each element of the RDD, and the return value is the new RDD.
	filter (*f*): Use the function *f* to filter out elements that do not meet the criteria. The return value is the new RDD.
	flatMap (*f*): Apply the function *f* to each element of the RDD, split the element data into an iterator, and the return value is the new RDD.
	mapPartitions (*f*): Similar to map, except that the input function of map () is applied to each element in the RDD, and the input function of mapPartitions () is applied to each partition.
	sample (*withReplacement, fraction, seed*): Return a new RDD that was generated by randomly sampling the original RDD.
	distinct (): Deduplication of elements in RDD.
	cartesian (*other RDD*): Find the Cartesian product of two RDDs.
	union (*other RDD*): Return a new RDD that contains all the elements of two RDDs.
	intersection (*other RDD*): Return a new RDD that contains the common elements of two RDDs.
	subtract (other RDD): Remove the same elements from the original RDD and the RDD parameter.
**Actions**	collect (): Return all elements of the RDD.
	count (): Return the number of elements in the RDD.
	countByValue(*n*): Return the number of occurrences of each element in the RDD.
	take (*n*): Return an array of the first n elements of the RDD.
	first (): Return the first element of the RDD.
	reduce (*f*): Use function *f* to integrate all elements of the RDD, such as sum operations.
	foreach (*f*): Run function *f* for each element of RDD.
	takeOrdered (*n, [ordering]*): Return first n elements from the RDD by default (ascending) or by specifying a collation.
	takeSample (*withReplacement, num, [seed]*): Return an array of randomly sampled num elements in the datasets.
	saveAsTextFile (*path*): Save all elements of the RDD as a text file to the local file system.

Table 2 Bioinformatics tools and algorithms based on Apache Spark

Function	Name	URL	Reference
Sequence	SparkSW	https://github.com/s0897918/SparkSW/	[10]
alignment/mapping	DSA	https://github.com/xubo245/DSA	[11]
	CloudSW	https://github.com/xubo245/CloudSW	[12]
	SparkBWA	https://github.com/citiususc/SparkBWA	[20]
	StreamBWA	https://github.com/HamidMushtaq/StreamBWA	[21]
	PASTASpark	https://github.com/citiususc/pastaspark	[24]
	PPCAS	https://github.com/jllados/PPCAS	[29]
	SparkBLAST	https://github.com/sparkblastproject/v2	[35]
	MetaSpark	https://github.com/zhouweiyg/metaspark	[36]
Sequence assembly	Spaler	Not Available	[41]
	SA-BR-Spark	Not Available	[43]
Sequence analysis	HiGene	Not Available	[48]
	GATK-Spark	Not Available	[49]
	SparkSeq	https://bitbucket.org/mwiewiorka/sparkseq/	[51]
Genome inference	SparkScore	Not Available	[72]
Phylogeny reconstruction	CloudPhylo	https://github.com/XingjianXu/cloudphylo	[63]
Drug discovery	S-CHEMO	Not Available	[67]
Single-cell RNA-seq	Falco	https://github.com/VCCRI/Falco/	[68]
Variant association and	VariantSpark	https://github.com/BauerLab/VariantSpark	[69]
population genetics studies	SEQSpark	https://github.com/statgenetics/seqspark	[71]
		https://www.assembla.com/spaces/roberts-lab-	[72]
Other	BioSpark	public/ wiki/Biospark	

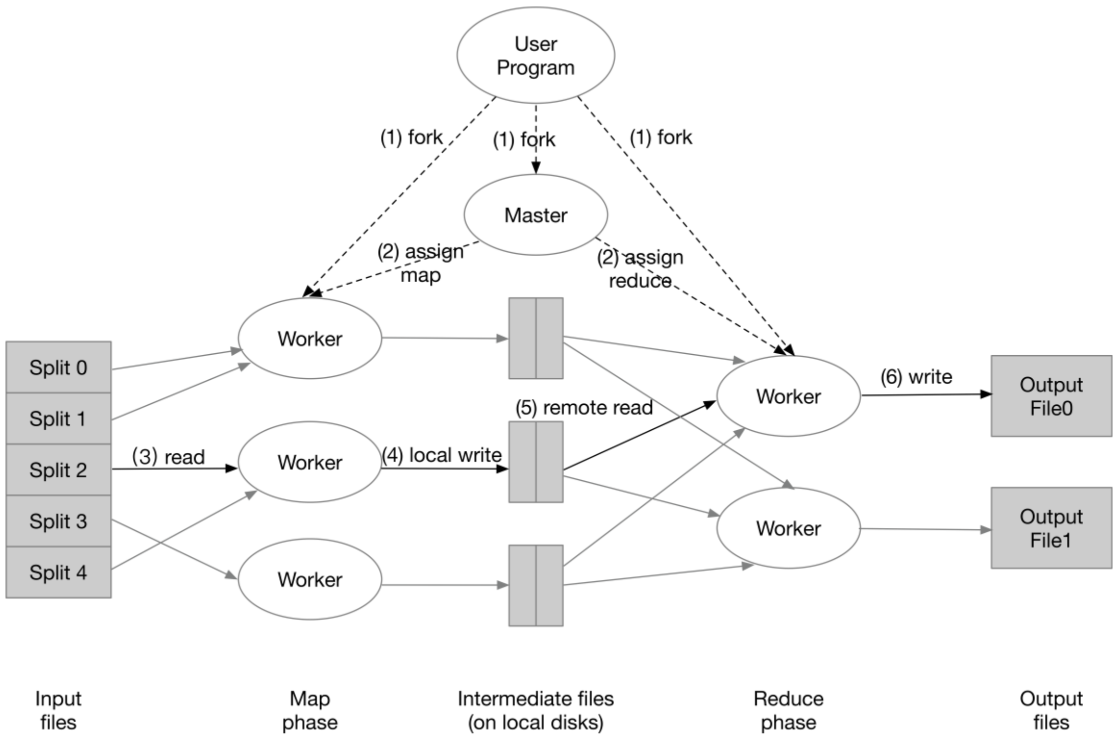

Figure 1 The operating mechanism diagram of Hadoop

Figure Click here to download Figure Figure 2.docx

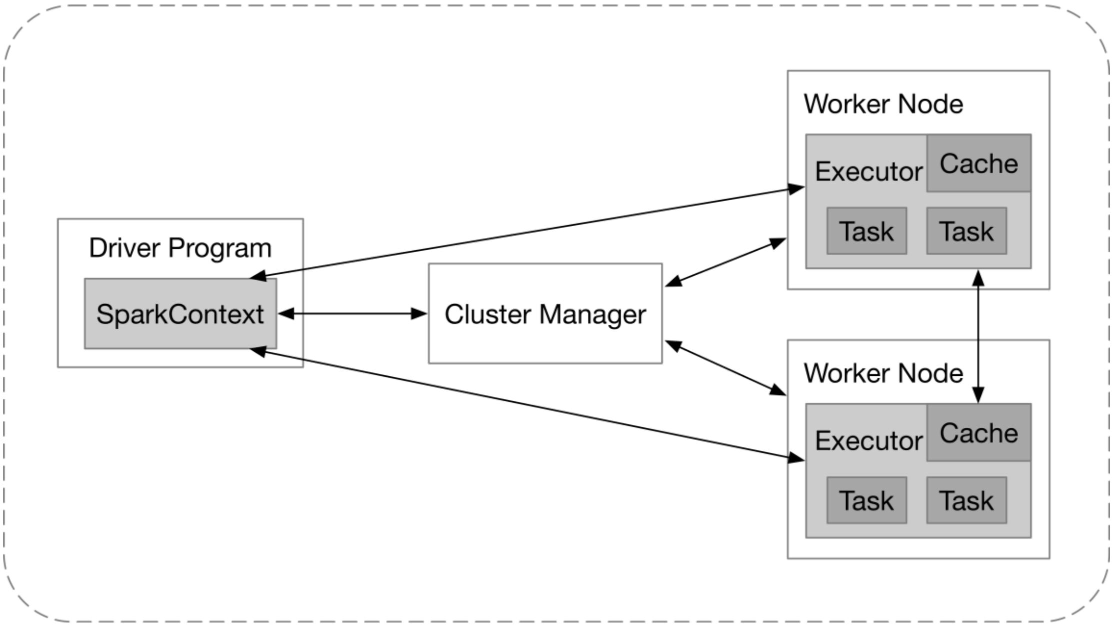

Figure 2 The architecture of Spark

Click here to download Figure Figure 3.docx

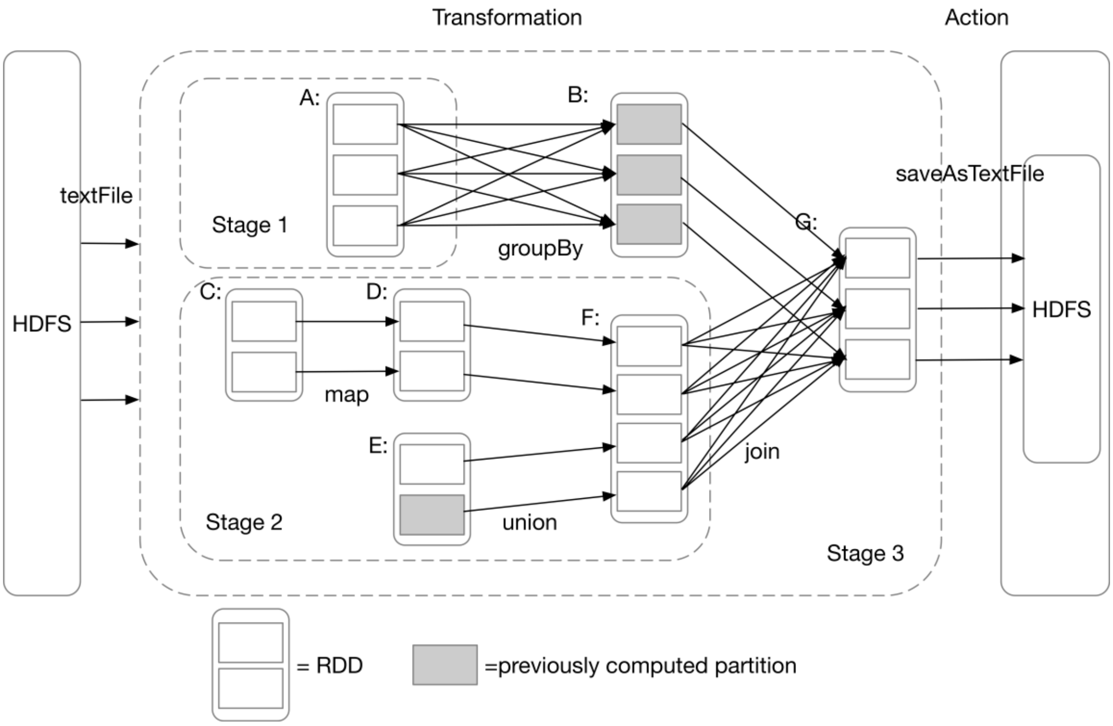

Figure 3 The task processing flow chart of Spark

 Click here to download Figure Figure 4.docx

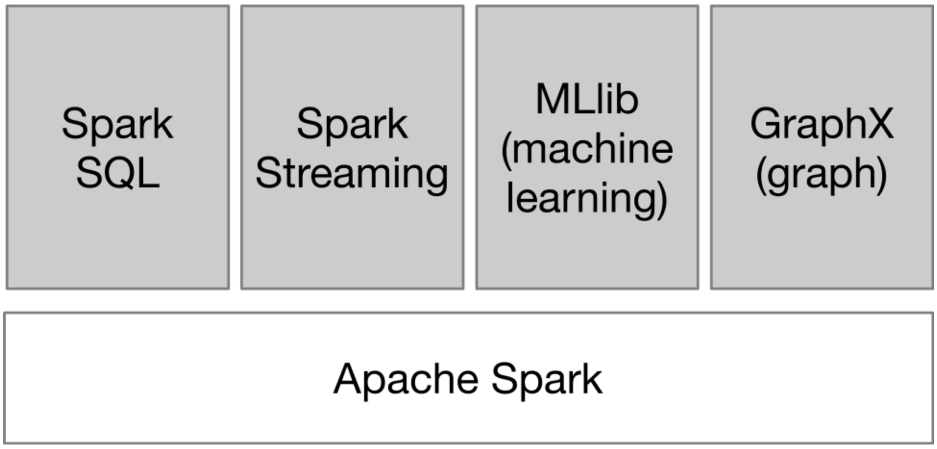

Figure 4 The Spark ecosystem

cover letter

Click here to access/download
Supplementary Material
Cover Letter.docx

GitHub wiki link

Click here to access/download
Supplementary Material
Home.md
