# Supplementary material for: Bioinformatics applications on Apache Spark
Source: Gigascience. 2018 Aug 7;7(8):giy098. doi: 10.1093/gigascience/giy098 (PMC6113509; doi:10.1093/gigascience/giy098)
Supplement: GIGA-D-18-00131_Revision_1.pdf [file giy098_giga-d-18-00131_revision_1.pdf]

GigaScience

Bioinformatics Application on Apache Spark

--Manuscript Draft--

**Manuscript Number:**	GIGA-D-18-00131R1
**Full Title:**	Bioinformatics Application on Apache Spark
**Article Type:**	Review
**Funding Information:**	National Key R&D Program of China (2017YFB0202600, 2016YFC1302500, 2016YFB0200400 and 2017YFB0202104) — Professor shaoliang peng<br>National Natural Science Foundation of China (61772543, U1435222, 61625202, 61272056 and 61771331) — Professor shaoliang peng<br>Guangdong Provincial Department of Science and Technology (2016B090918122) — Professor shaoliang peng
**Abstract:**	With the rapid development of next-generation sequencing (NGS) technology, the ever-increasing genomic data pose a tremendous challenge to data processing. Therefore, there is an urgent need for highly scalable and powerful computational systems. Among the state-of–the-art parallel computing platforms, Apache Spark is a fast, general-purpose, in-memory, iterative computing framework for large-scale data processing, which ensures high fault tolerance and high scalability by introducing the resilient distributed dataset (RDD) abstraction. In terms of performance, Spark can be up to 100x faster in memory access and 10x faster in disk access than Hadoop. Moreover, it provides advanced APIs in Java, Scala, Python, and R. It also supports some advanced components, including Spark SQL for structured data processing, MLlib for machine learning, GraphX for graph computing, and Spark Streaming for stream computing. In this paper, we surveyed Spark-based applications in the NGS and other biological domains, such as epigenetics, phylogeny, and drug discovery. We believe that this survey provides a comprehensive guideline for bioinformatics researchers to apply Spark in their own fields.<br>Keywords: next-generation sequencing; bioinformatics; Apache Spark; resilient distributed dataset; memory computing
**Corresponding Author:**	runxin guo<br><br>CHINA
**Corresponding Author Secondary Information:**	
**Corresponding Author's Institution:**	
**Corresponding Author's Secondary Institution:**	
**First Author:**	runxin guo
**First Author Secondary Information:**	
**Order of Authors:**	runxin guo<br>yi zhao<br>xiangke liao<br>kenli li<br>quan zou<br>xiaodong fang<br>shaoliang peng
**Order of Authors Secondary Information:**	

Powered by Editorial Manager® and ProduXion Manager® from Aries Systems Corporation

Response to Reviewers:	Dear Editor and Reviewers:<br>Thank you for your letter and for the reviewers' comments concerning our manuscript entitled "Bioinformatics Application on Apache Spark" (GIGA-D-18-00131). Those comments are all valuable and very helpful for revising and improving our paper, as well as the important guiding significance to our research. We have studied the comments carefully and have made correction which we hope meet with approval. Revised portion are marked in red in the paper. The main corrections in the paper and the responds to the editor and reviewers' comments are as following:<br>To Editor:<br>Comment 1: While one of the reviewers doesn't like the figures, another thinks you need more, and generally we side with the "more figures in a review the better". In particular, we would encourage improvements and more infographic like detail if you can. Improvements to the language and writing is also required, although if it passes review we will send it to a copy editor.<br>Response: Appreciate for your comment, taking into account the reviewers' comments and your opinions, we have improved the figures, removed unnecessary figures, and added a few necessary figures to help readers better understand the Spark framework and operating mechanism. In addition, we have improved my language and writing.<br>To Reviewer 1:<br>Comment 1: The paper would benefit from a *slightly* deeper description of the Spark architecture, in particular explaining the nature of DAGs and the way in which they permit optimizations. Also, some mention of the two deploy modes (where the driver program can either be run on the client machine, or on a worker node).<br>Response: Appreciate for your comment, in "THE SPARK FRAMEWORK" section, we have made a more detailed description of the Spark architecture, explained the nature of DAGs and the way in which they permit optimizations, introduced the two deploy modes: cluster mode and client mode.<br>Comment 2: The paper would also benefit from a section that examines the potential downsides of using Spark, for example the potential complexity in creating and maintaining a Spark cluster, and the learning curve involved in learning a new API and perhaps even language (especially given the Functional Programming nature of the API).<br>Response: Appreciate for your comment, in "DISCUSSION" section, we have discussed the disadvantages of Spark, including the applications that Spark is not suitable for, the complexity of creating and maintaining a Spark cluster, the time cost of large-scale input data from local to remote servers in slow networks, the complex learning curve.<br>Comment 3: With regards to style, there are a number of places in the paper where the definite article is used where it shouldn't, and vice versa. In the interest of readability and not distracting the reader, these should be addressed. A similar point can be made with regard to the over-use of certain prepositions (e.g. "besides"), which are called out in detail in the next section.<br>Response: Appreciate for your comment, we have addressed the use of the definite article in paper, and replaced "besides" with "in addition", "moreover", and "furthermore".<br>Comment 4: p.1 line 28: "data" is treated as a plural in the rest of the paper, therefore "pose" rather than "poses".<br>Response: Appreciate for your comment, we have changed "poses" to "pose".<br>Comment 5: p.1 line 34: "by introducing resilient distributed dataset" should be "by introducing the resilient distributed dataset" (i.e. use of definite article)<br>Response: Appreciate for your comment, we have changed "by introducing resilient distributed dataset" to "by introducing the resilient distributed dataset".<br>Comment 6: p.1 line 40: In the end, we discussed the challenges...and the future work...". I haven't found this discussion in the paper.<br>Response: Appreciate for your comment, we have deleted this sentence from "ABSTACT" section, but added a "DISCUSSION" section to discuss the advantages and disadvantages of Spark, some issues to be considered about cloud computing in the future and other bioinformatics fields that have not yet been involved.<br>Comment 7: p.2 line 4: "MapReduce preforms" should be "MapReduce performs".<br>Response: Appreciate for your comment, we have changed "preforms" to "performs".<br>Comment 8: p.2 line 21: "introducing resilient distributed dataset" should "introducing the resilient distributed dataset".<br>Response: Appreciate for your comment, we have changed "introducing resilient distributed dataset" to "introducing the RDD abstraction".

	Comment 9: p.2 line 38: The documentation of Spark describes the driver program as "The process running the main () function of the application and creating the SparkContext". It does not "deploy the Spark operating environment". Perhaps the authors meant "deploy TO the Spark operating environment" but even here this would be incorrect, as the spark-submit script does this.<br>Response: Appreciate for your comment, we have rewritten this part to provide the correct description in “THE SPARK FRAMEWORK” section.<br>Comment 10: p.2, line 43: As well as Scala, Spark provides APIs in Java, Python and more recently R. This flexibility is important to researchers when deciding whether to use Spark or not.<br>Response: Appreciate for your comment, we have mentioned that Spark provides advanced APIs in Java, Scala, Python and R in “THE SPARK FRAMEWORK” section.<br>Comment 11: p.2, line 58: It is questionable that "the most important feature of RDD" is the fault tolerance. Certainly, it is "an important feature".<br>Response: Appreciate for your comment, we have changed this part to “RDD achieves fault tolerance through a notion of lineage…” in “THE SPARK FRAMEWORK” section.<br>Comment 12: p.3, line 13: The referenced image appears to be an _example_ of a spark task flow chart, rather than _the general_ Spark task processing flow. For the reader's sake, the paper should either describe what this particular task is doing (including the fact that it is reading and writing to HDFS in this case). Otherwise the reader may form incorrect opinions or simply be confused. Alternatively, drop the figure entirely.<br>Response: Appreciate for your comment, we have updated this figure to show an example of how Spark computes job stages in “THE SPARK FRAMEWORK” section.<br>Comment 13: p.3, line 17: "Besides" as preposition. This is a little colloquial and has an additional "in any case" meaning. To avoid distracting the reader, consider replacing "besides" as a preposition with alternatives like "In addition", "Moreover", "Furthermore". This can be applied to the rest of the paper, and I won't call any more out by line number.<br>Response: Appreciate for your comment, we have replaced “besides” with “in addition”, “moreover”, and “furthermore” in paper.<br>Comment 14: p.4, line 4: "Burrow-Wheeler aligner" - either "The Burrow-Wheeler aligner" or "Burrow-Wheeler alignment" read better<br>Response: Appreciate for your comment, we have changed "Burrow-Wheeler aligner" to “The Burrow-Wheeler aligner”.<br>Comment 15: p.4, line 19: "Results showed" - "The results showed"<br>Response: Appreciate for your comment, we have changed “Result showed” to “The results showed”.<br>Comment 16: p.4, line 32: "achieved the average speedup of" - "achieved an average speedup of"<br>Response: Appreciate for your comment, we have changed “achieved the average speedup of” to “achieved an average speedup of”.<br>Comment 17: p.6, line 58: Drop "And" from the start of the sentence.<br>Response: We have dropped “And” from the start of the sentence.<br>Comment 18: p.7, line 1: "Experiments results" - "Experimental results”<br>Response: Appreciate for your comment, we have changed “Experiments results” to “Experimental results”.<br>Comment 19: p.7, line 4: Perhaps it's worth pointing out that this is an example of the platform itself suggesting a new algorithm, rather than simply re-implementing an existing algorithm on the new platform. Similarly, for line 19 of this page.<br>Response: Appreciate for your comment, we have pointed out that these two are examples of the Spark platform itself suggesting new algorithms in “SPARK IN ASSEMBLY” section.<br>Comment 20: p.7, line 23: Is SA-BR-MR running on Hadoop? (I ask because MR is a valid algorithm on Spark as well).<br>Response: Appreciate for your comment, SA-BR-MR is running on Hadoop according to the reference paper.<br>Comment 21: p.8, line 17: "Results..." - "The results..."<br>Response: Appreciate for your comment, we have changed “Results” to “The results”.<br>Comment 22: p.8, line 41: "noises" - "noise".<br>Response: Appreciate for your comment, we have changed “noises” to “noise”.<br>Comment 23: p.9, lines 23-39: The epigenetics example just calls out the advantage of parallelization compared to sequential processing. Was there a parallelized attempt, perhaps using Hadoop, that the Yu N et al paper could demonstrate a superiority to?

	Response: Appreciate for your comment, we have reviewed lots of related papers, but did not find some parallelized attempts.<br>Comment 24: p.10, line 8: "Saprk" - "Spark"<br>Response: Appreciate for your comment, we have changed “Saprk” to “Spark”.<br>Comment 25: p.10, line 15: The term "checkpointing" is not explained even in the body of the referenced paper (Harnie D et al) and is probably best dropped.<br>Response: Appreciate for your comment, we have dropped the term “checkpointing” from the sentence.<br>Comment 26: p.11, line 43: Key Points section: I would respectfully disagree with the following statement: "We introduce the Apache Spark framework in detail, helping researchers to understand its architecture, programming model and processing mechanism." I think the authors do a good job of firstly, giving an *overview* of Spark (notwithstanding earlier points about getting into more detail), but I don't think this paper is a *detailed* description of Spark, its architecture or its programming model. Indeed, I don't think it *needs* to be - the survey of *how Spark has successfully been used* is probably of primary interest to most readers. But it's best to be clear about the scope of the paper in the Key Points so as to set readers' expectations correctly.<br>Response: Appreciate for your comment, we have updated this key point to point out that we outline the Apache Spark framework to researchers to understand its architecture, programming model and processing mechanism, and we have made a more detailed description of the Spark architecture in “THE SPARK FRAMEWORK” section.<br>Comment 27: p.11, line 48: Key Points section: Similarly, to above, I would edit the third Key Point to set readers' expectations correctly. The paper in its current form does not include a "discussion on the future of parallel computing in bioinformatics" (and in my opinion it does not need to).<br>Response: Appreciate for your comment, considering your opinions and that of other reviewers, we have added a “DISCUSSION” section to discuss Spark’s strengths, weaknesses, and challenges faced in this field.<br>To Reviewer 2:<br>Comment 1: While I agree, Spark has a lot of advantages over other parallel and distributed computing frameworks such as MapReduce, I feel the current tone and content are too one-sided. In my own experience, Spark is mostly only useful for processing very large amount of data. For smaller data sets, the scalability gained by Spark may not be enough to justify the up-front time required for setting up and configuring a Spark-enabled system. Also, there is no discussion on computing hardware requirement (local computer cluster or commercial cloud computing platforms), and issues related to transfer of large data sets over the Internet. All these issues need to be discussed.<br>Response: Appreciate for your comment, we have discussed the strengths and weaknesses of Spark and issues related to transfer of large data sets over the Internet on local computer cluster or commercial cloud computing in “DISCUSSION” section, and pointed out the official proposal for hardware requirements in “THE SPARK FRAMEWORK” section.<br>Comment 2: The sections on 'Spark in motif analysis' and 'Spark in genomic inference' are poorly written. The terms 'motif' and 'genomic inference' are not properly defined. Do they mean transcription factor binding motifs, or simply frequently occurring DNA sequence some defined regions in the genome (e.g., promoters, enhancers, etc.)? Also, the term 'genomic data inference' is not well defined. Presumably the authors are referring to inference in a population genomics context.<br>Response: Appreciate for your comment, here, motif refers to transcription factor binding sites (TFBS), genomic inference refers to the inference in population genomics text. We have updated these two sections in the paper to provide correct descriptions about the terms ‘motif’ and ‘genomic inference’.<br>Comment 3: The caption of all four figures are way too simple. In most cases, especially for complicated flow diagrams like Fig 1 and Fig 3, there is no explanation or description of the content of the figure. I believe the authors intends to illustrate the inner working of Spark using these figures. Nonetheless, the content of these figures is not explained in the caption nor the main text.<br>Response: Appreciate for your comment, considering your opinions and that of other reviewers, we have updated the figures, removed unnecessary figures, and added a few necessary figures to help readers better understand the Spark framework and operating mechanism. Moreover, we have added some explanations and descriptions of the content of the figures in the captions.

Powered by Editorial Manager® and ProduXion Manager® from Aries Systems Corporation

	Comment 4: Despite the authors claiming they 'discuss the future of parallel computing in bioinformatics' (Key Points #3), the manuscript barely talks about the future, other than saying 'Spark will provide promising performance for biological researchers in the future' (Conclusions). I think this is a lost opportunity. What do the authors see as the major limitations in the field at the moment? New hardware? Better integration with cloud computing platforms? New application areas, such as proteomics, metabolomics, biomedical text, electronic medical health record, etc.?<br>Response: Appreciate for your comment, we have updated the Key Points #3 and discussed some issues to be considered about cloud computing in the future and pointed out other bioinformatics fields that have not yet been involved.<br>Comment 5: In multiple occasions, the authors use 'And' to begin a sentence. I do not think it is grammatically correct.<br>Response: Appreciate for your comment, we have dropped "And" from all related sentences in paper.<br>Comment 6: Throughout the manuscript, author names are often cited as ([last name] [first initials]), but sometimes they are cited as ([last name] [all initials]) or ([last name] [first name]). Please make sure names are formatted consistently.<br>Response: Appreciate for your comment, we have updated author names as ([last name] [first initial]) in paper to make sure names are formatted consistently.<br>To Reviewer 3:<br>Comment 1: The manuscript needs to be rewritten to provide more practical information to bioinformatics research users how Apache Spark based bioinformatics tools are actively used in specific bioinformatics research domains and what are the advantages of using the Apache Spark.<br>Response: Appreciate for your comment, we have rewritten the paper to provide more practical information to bioinformatics research users how Apache Spark based bioinformatics tools are actively used in specific bioinformatics research domains and what are the advantages of using the Apache Spark. In "THE SPARK FRAMEWORK" section, we have made a more detailed description of the Spark architecture and the main abstraction RDD, explained the nature of DAGs and the way in which they permit optimizations, provided the official proposal for hardware requirements to help researchers better understand and use Spark. Moreover, we have added the "DISCUSSION" section to discuss the strengths and weaknesses of Spark, the applications that Spark is suitable for, and some issues must be considered. Researchers can comprehensively consider how to use Spark through these contents combined with biological issues in their field.<br>Comment 2: Table 1 shows basic APIs of Apache Spark. The reviewer cannot get a point why the authors included this table. Delete the table or keep the table with presenting how the APIs could be used in the bioinformatics tools.<br>Response: Appreciate for your comment, we have removed the Table 1 from the paper.<br>Comment 3: In the first paragraph of the Introduction section, "However, the existing bioinformatics tools cannot effectively handle such a large amount of data. In order to solve the issues, MapReduce, a programming model for parallel computation of large datasets, has been proposed [1]." sentences should be updated. MapReduce has not been proposed for bioinformatics tool. MapReduce framework was proposed for general purpose big data analysis in the distributed manner.<br>Response: Appreciate for your comment, we have updated these sentences to point out that MapReduce was proposed for processing large-scale datasets in a distributed manner in information technology rather than for bioinformatics tools.<br>Comment 4: The manuscript did not mention about "DataFrames" API that is an extension of RDD. Most of new Spark features use this new DataFrames, and it should be addressed in the manuscript.<br>Response: Appreciate for your comment, we have mentioned the two extensions of RDD: DataFrame and Dataset in "The SPARK FRAMEWORK" section. Users can seamlessly switch between the three through simple API calls.<br>Comment 5: Table 2 only shows the application domain, program name, URL, references. The table should be updated to include more meaningful informatics of tools such as pros/cons or specific features or the tools.<br>Response: Appreciate for your comment, we have updated the Table 2 to provide more meaningful informatics, including name, function, features, pros/cons and reference of applications.<br>Comment 6: Figures 1 to 4 are not necessary in the manuscript. Instead of these figures, the authors should consider how to express the relationship of bioinformatics

	tools and Apache Spark effectively using figures.<br>Response: Appreciate for your comment, considering the opinions of editor and other reviewers, we have improved the figures, removed unnecessary figures, and added a few necessary figures to help readers better understand the Spark framework and operating mechanism. Figure 1 is mainly used to introduce Spark's cluster architecture, explain its main components and functions, and how the Spark application runs on the cluster. Figure 2 is mainly used to show some examples of narrow and wide dependencies to help readers understand RDD's dependencies. Because these dependencies are important in the splitting job stages of Spark, so we add this figure. Figure 3 is mainly used to show an example of how Spark computes job stages to help readers understand the RDD operating mechanism and DAG scheduling. So, we add this figure. Moreover, we carefully considered your comments about how to express the relationship of bioinformatics tools and Apache Spark effectively using figures. We feel that the relationship between bioinformatics tools and Spark cannot be well expressed in figures. Because the variated items in bioinformatics can cover almost all sorts of computational optimization problems. The Spark framework represents a possible solution for tasks in parallel computing with some certain characteristics. So, we tried our best to provide readers with some practical advices on using Spark framework based on computational characteristics of problems in "Discussion" section. In "DISCUSSION" section, we have discussed the strengths and weaknesses of Spark, the applications that Spark is suitable for, and some issues must be considered to help researchers to consider how to use Spark combined with biological issues in their field. We tried our best to improve the manuscript and made some changes in the manuscript. These changes will not influence the content and framework of the paper. And here we did not list the changes but marked in revise paper. We appreciate for Editor/Reviewers' warm work earnestly, and hope that the correction will meet with approval. Once again, thank you very much for your comments and suggestions.<br>Yours<br>Sincerely<br>Runxin GUO, Yi ZHAO, Xiangke LIAO, Kenli LI, Quan ZOU, Xiaodong FANG, Shaoliang PENG
**Additional Information:**	
**Question**	**Response**
Are you submitting this manuscript to a special series or article collection?	No
**Experimental design and statistics**<br><br>Full details of the experimental design and statistical methods used should be given in the Methods section, as detailed in our Minimum Standards Reporting Checklist. Information essential to interpreting the data presented should be made available in the figure legends.<br><br>Have you included all the information requested in your manuscript?	Yes
**Resources**<br><br>A description of all resources used, including antibodies, cell lines, animals and software tools, with enough information to allow them to be uniquely identified, should be included in the Methods section. Authors are strongly encouraged to cite Research Resource	Yes

Identifiers (RRIDs) for antibodies, model organisms and tools, where possible.<br><br>Have you included the information requested as detailed in our Minimum Standards Reporting Checklist?	
**Availability of data and materials**<br><br>All datasets and code on which the conclusions of the paper rely must be either included in your submission or deposited in publicly available repositories (where available and ethically appropriate), referencing such data using a unique identifier in the references and in the "Availability of Data and Materials" section of your manuscript.<br><br>Have you have met the above requirement as detailed in our Minimum Standards Reporting Checklist?	Yes

Manuscript
Click here to download Manuscript Bioinformatics Application on Apache Spark.docx

Bioinformatics Application on Apache Spark

Runxin GUO[1†], Yi ZHAO[3†], Xiangke LIAO[1], Kenli LI[2], Quan ZOU[4*], Xiaodong FANG[5*], Shaoliang PENG[1,2†*]

[1]College of Computer, National University of Defense Technology, Changsha 410073, China

[2]College of Computer Science and Electronic Engineering & National Supercomputer Centre in Changsha, Hunan University, Changsha 410082, China

[3]Institute of Computing Technology, Chinese Academy of Sciences, Beijing 100190, China

[4]School of Computer Science and Technology, Tianjin University, Tianjin 300350, China

[5]BGI Genomics, BGI-Shenzhen, Shenzhen 518083, China

pengshaoliang@nudt.edu.cn ; zouquan@nclab.net ; fangxd@bgitechsolutions.com

*: corresponding author; †: equal contributors

ABSTRACT

With the rapid development of next-generation sequencing (NGS) technology, the ever-increasing genomic data pose a tremendous challenge to data processing. Therefore, there is an urgent need for highly scalable and powerful computational systems. Among the state-of–the-art parallel computing platforms, Apache Spark is a fast, general-purpose, in-memory, iterative computing framework for large-scale data processing, which ensures high fault tolerance and high scalability by introducing the resilient distributed dataset (RDD) abstraction. In terms of performance, Spark can be up to 100x faster in memory access and 10x faster in disk access than Hadoop. Moreover, it provides advanced APIs in Java, Scala, Python, and R. It also supports some advanced components, including Spark SQL for structured data processing, MLlib for machine learning, GraphX for graph computing, and Spark Streaming for stream computing. In this paper, we surveyed Spark-based applications in the NGS and other biological domains, such as epigenetics, phylogeny, and drug discovery. We believe that this survey provides a comprehensive guideline for bioinformatics researchers to apply Spark in their own fields.

Keywords: next-generation sequencing; bioinformatics; Apache Spark; resilient distributed dataset; memory computing

INTRODUCTION

NGS technology has generated huge amounts of biological sequence data. In order to use these data efficiently, we need to store and analyze the data accurately and efficiently. However, the existing bioinformatics tools cannot effectively handle such a large amount of data. Therefore, there is an urgent need for scalable and powerful distributed computing tools to solve this problem. In the field of information technology, MapReduce [1] is a distributed parallel programming model and

methodology for processing large-scale datasets. It splits large-scale datasets into many key-value pairs through both the map and reduce phases, significantly improving performance and showing good scalability. By combining the Hadoop Distributed File System (HDFS) and MapReduce, Apache Hadoop can enable distributed processing of large amounts data in a reliable, efficient, and scalable way, where HDFS is mainly used for distributed storage of massive datasets and MapReduce performs distributed computing on these datasets. As a result, Hadoop has been adopted by the bioinformatics community in several areas [2], such as alignment [3], mapping [4] and sequence analysis [5].

However, due to its disk-based I/O access pattern, intermediate calculation results are not cached. Therefore, Hadoop is only suitable for batch data processing, and shows poor performance for iterative data processing. To resolve this problem, Apache Spark [6] has been proposed, which is a faster general-purpose computing framework designed specifically to handle huge amounts of data. Unlike Hadoop's disk-based computing, Spark performs memory computing by introducing the RDD abstraction. Since it is possible to store intermediate results in memory, it is more efficient for iterative operations. In terms of performance, Spark can be up to 100x faster in memory access than Hadoop [6]. Even if we compare between them based on the performance of the disk, the gap is more than 10 times [7]. In terms of flexibility, Spark provides high-level APIs in Java, Scala, Python, and R, and interactive shell. In terms of generality, Spark provides structured data processing, machine learning, graph computing, and stream computing capabilities by supporting some advanced components.

THE SPARK FRAMEWORK

Spark is an open source cluster computing environment designed for large-scale data processing, developed by UC Berkeley AMP lab. It provides advanced APIs in Java, Scala, Python and R, and an optimized engine that supports general execution graphs. It also supports some advanced components, including Spark SQL for structured data processing, MLlib for machine learning, GraphX for graph computing, and Spark Streaming for stream computing.

As in Figure 1, Spark application runs as independent processes on the cluster and are coordinated by the SparkContext in the driver program. There are two types of deploy modes depending on where the driver program is running: cluster mode and client mode. In the former, driver program runs on a worker node. In the latter, driver program runs on the client machine. First, SparkContext requests the executors on the worker nodes in the cluster from the cluster manager (either Spark's own Standalone cluster manager, Apache Mesos, or Hadoop YARN). These executors are processes that can run tasks and store data in memory or on disk for application. Next, the SparkContext will

send tasks to the executors to perform. Finally, the executors return the results to the SparkContext after the tasks are executed. In Spark, an application generates multiple jobs. A job is split into several stages. Each stage is a task set containing several tasks, which performs some calculations and produces some intermediate results. Task is the smallest unit of work in Spark, completing a specific thing on an executor.

As the main abstraction in Spark, RDD is a read-only collection of objects partitioned on different nodes in the cluster so that the data in RDD can be processed in parallel. The data in RDD are stored in memory by default, but Spark automatically writes RDD data to disk if memory resources are low. RDD achieves fault tolerance through a notion of lineage [6], that is, if an RDD partition on a node is lost because of a node failure, the RDD automatically recalculates the partition from its own data source. Moreover, Spark provides two types of operations on RDD: transformation and action. The former defines a new RDD, and the latter returns a result or writes RDD data to the storage system. Transformation employs lazy operation [8], which means that the operation of generating another RDD from one RDD transformation is not executed immediately, and the calculation process is not actually started until an action is performed. Furthermore, each transformation operation generates a new RDD, the newly generated RDD depends on the original RDD. According to the different types of transformation operations, RDD's dependencies can be divided into narrow dependency and wide dependency. The former refers to that each partition in the generated RDD only depends on the parent RDD fixed partition, and the latter refers to the fact that each partition of the generated RDD depends on all partitions of the parent RDD. Figure 2 shows examples of narrow and wide dependencies. In addition, Spark also provides two extensions of RDD: DataFrame and Dataset. Spark users can seamlessly switch between the three through simple API calls.

Furthermore, Spark adopts a directed acyclic graph (DAG) [9] to optimize execution process by splitting the submitted jobs into several stages according to the wide dependency. For narrow dependency, it divides related transformation operations into the same stage because they can perform pipelining operations and thus reduces the processing time of submitted jobs. Figure 3 shows an example of how Spark computes job stages. In addition, if the partitions on a node are lost because of a node failure, Spark can utilize the DAG to recalculate the lost partitions.

As for the hardware requirements, the official proposal is to have 4 to 8 disks per node, configure at least 8GB memory and 8 to 16 CPU cores per machine, and use a 10 Gigabit or higher network.

SPARK IN ALIGNMENT AND MAPPING

The rapid development of NGS technology has generated a large amount of sequence data (reads), which has a tremendous impact on sequence alignment and mapping process. Currently, the sequence alignment and mapping process still consume a lot of time.

The Smith-Waterman (SW) algorithm [10], which produces the optimal local alignment between two strings of nucleic acid sequences or protein sequences, is widely used in bioinformatics. However, SW algorithm requires a high computational cost due to high computational complexity. To speed up the algorithm, in 2015, Zhao G *et al* implemented the SW algorithm on Spark for the first time, called as SparkSW [11]. It consisted of three phases: data preprocessing, SW as map tasks and top K records as reduce tasks. Experimental results [11] showed that SparkSW was load-balancing and scalable with computing resources increased.

However, SparkSW merely supports SW algorithm without the mapping location and traceback of optimal alignment, as a result, SparkSW executes slowly. Therefore, in 2017, Xu B *et al* proposed DSA [12], which employed Single Instruction Multiple Data (SIMD) instruction to parallel the sequence alignment algorithm at each worker node. Experimental results [12] showed that DSA achieved up to 201x speedup over SparkSW and almost linear speedup with the increase of cluster nodes.

Subsequently, Xu B *et al* proposed CloudSW [13], an efficient distributed SW algorithm which leveraged Spark and SIMD instructions to accelerate the algorithm and provided APIs service in the cloud. Experimental results [13] showed that CloudSW achieved up to 3.29x speedup over DSA and 621x speedup over SparkSW. CloudSW also showed excellent scalability and achieved up to 529 giga cell updates per second (GCUPS) in protein database search with 50 nodes in Aliyun.

The Burrows-Wheeler aligner (BWA) is composed of BWA-backtrack [14], BWA-SW [15] and BWA-MEM [16] for performing sequence alignment and mapping in bioinformatics. Before the advent of Spark-based BWA tool, there were several other BWA tools based on big data technology, including BigBWA [17], Halvade [18] and SEAL [19]. However, they were based on Hadoop showing limited scalability and complex implementation.

As a result, in 2015, Al-Ars Z *et al* [20] implemented three different versions of BWA-MEM and compared their performance: a native cluster-based version, a Hadoop version and a Spark version. Three implementations were evaluated on the same IBM Power7 and Intel Xeon servers with the WordCount example. The results [20] showed that simultaneous multithreading improved the performance of three versions of BWA-MEM, and the Spark version with 80 threads increased performance by up to 87% than the native cluster version using 16 threads. Furthermore, the Hadoop

version with 4 threads increased performance by 17% and the Spark version with more threads increased performance by 27%.

After then, in 2016, Abuín J *et al* proposed SparkBWA [21] which is composed of three main phases: the RDDs creation phase, the map phase, and the reduce phase. Experimental results [21] showed that for the BWA-backtrack algorithm, SparkBWA achieved an average speedup of 1.9x and 1.4x compared with SEAL and pBWA respectively. For the BWA-MEM algorithm, SparkBWA was 1.4x faster than BigBWA and Halvade tools on average.

However, SparkBWA required the data availability in the HDFS. In general, the input files were given in gzip format, which required first uncompressing the file before uploading it to the HDFS. Subsequently, this also slowed down the execution of BWA itself, since data on the HDFS had to be reformatted as appropriate input to the BWA program tasks running on the cluster. Finally, the output files produced by those BWA tasks required significant time to combine separately at the end.

Therefore, in 2017, Mushtaq H *et al* employed Spark to propose StreamBWA [22], where the input data was being streamed directly from a compressed file. This file could either be located on the master node or on a URL, which eliminated the cost of execution time of downloading the file and then uncompressing it. Moreover, since the master node could stream data to the data nodes, the overhead of uploading data to the HDFS could also be hidden. The master node could also start combining the output files of BWA tasks running on the data nodes, in parallel, once they were available, further reducing the overall time. Experimental results [22] showed that this streaming distributed approach was approximately 2x faster than the non-streaming approach. Furthermore, StreamBWA was 5x faster than SparkBWA.

Multiple sequence alignment (MSA) refers to the sequence alignment of three or more biological sequences, such as protein or nucleic acid sequences. One of representative tools for performing MSA is PASTA [23]. PASTA is a derivative of SATé [24], which produces highly accurate alignments in shared memory computers. However, PASTA is limited to processing small and medium datasets, because the computing power of shared memory systems cannot meet the memory and time requirements of large-scale datasets.

Therefore, in 2017, Abuín J *et al* proposed PASTASpark [25], which allowed executions on a distributed memory cluster taking advantage of Spark. It employed an in-memory RDD of key-value pairs to parallel the calculating MSAs phase. Experiments were conducted on two different clusters (CESGA and AWS). The results [25] showed that PASTASpark achieved up to 10x speedups compared with single-threaded PASTA and was able to process 200,000 sequences in 24

hours using only AWS nodes. Therefore, PASTASpark ensured scalability and fault tolerance which greatly reduced the time to obtain MSA.

The probabilistic pairwise model [26] is widely used in all consistency-based MSA tools, such as MAFFT [27], ProbCons [28] and T-Coffee(TC) [29]. However, the global distributed memory cannot meet the ever-increasing sequence datasets, which causes the need of specialized distributed databases, such as HBase or Cassandra. As a result, in 2017, Lladós J *et al* employed Spark to propose a new tool, PPCAS [30], which could parallel the probabilistic pairwise model for large-scale protein sequences and store it in a distributed platform. Experimental results [30] showed that it was better with single node and provided almost linear speedup with the increase in the number of nodes. In addition, it could compute more sequences using the same memory.

NCBI BLAST [31, 32] is widely used to implement algorithms for sequence comparison. Before the Spark-based BLAST was created, several other BLAST tools had been proposed including mpiBLAST [33], GPU-BLAST [34] and CloudBLAST [35]. However, with the increasing number of genomic data, these tools showed limited scalability and efficiency.

As a result, in 2017, Castro M *et al* proposed SparkBLAST [36], which utilized cloud computing and Spark framework to parallel BLAST. In SparkBLAST, Spark's *pipe* operator and RDDs were utilized to call BLAST as an external library and perform scalable sequence alignment. It was compared with CloudBLAST on both Google and Microsoft Azure Clouds. Experimental results [36] showed that SparkBLAST outperformed CloudBLAST in terms of speedup, scalability and efficiency.

Metagenomics is crucial for studying genetic material directly from environmental samples. Fragment recruitment is the process of aligning reads to reference genomes in metagenomics data analysis. In 2017, Zhou W *et al* proposed MetaSpark [37], which employed Spark to recruit metagenomics reads to reference genomes.

MetaSpark utilized the RDD of Spark to cache datasets in memory and scaled well along dataset size increments. It consisted of five steps including constructing k-mer RefindexRDD, constructing k-mer ReadlistRDD, seeding, filtering, and banded alignment. It was evaluated on a ten-node cluster working under the Spark standalone module where each node contained an 8-core CPU and 16 GB RAM. It employed about one million 75bp Illumina reads dataset and two references (the 194 human gut genomes and the bacterial genomes) that were respectively 0.616GB and 1.3GB in size. Experimental results [37] showed that MetaSpark recruited more reads than FR-HIT [38] with the same parameters and 1 million reads. MetaSpark recruited 501,856 reads when there were 0.616 GB human gut genome references, while FR-HIT recruited 489,638 reads. MetaSpark increased

recruited reads by 2.5%. When references changed to a 1.3 GB bacterial genome, MetaSpark recruited 463,862 reads, while FR-HIT recruited 444,671 reads. MetaSpark increased recruited reads by 4%. Moreover, the results also showed that MetaSpark offered good scalability. Under a 0.616 GB reference, run time for 0.1 million reads was 51 min under 4 nodes, and decreased slightly to 23.5 min under 10 nodes. For the 1 million read datasets, MetaSpark would crash under 4 nodes due to limited memory. Under 6 nodes, it finished running after 312 min and would sharply decrease to 201 min under 10 nodes.

SPARK IN ASSEMBLY

Due to short lengths of the NGS reads (<500 bp), they need to be assembled prior to further analysis, which is another important phase in sequence analysis workflow. In general, there are two types of assembly: the reference assembly and *de novo* assembly. The assembly algorithm includes two categories: overlap-layout-consensus (OLC) algorithm and the de Bruijn graph algorithm. The former is generally employed to assemble longer reads, while the latter shows a good performance in assembling short reads.

Before Spark-based distributed memory de novo assemblers were created, although there were some MPI-based assemblers (such as Ray [39], AbySS [40] and SWAP-Assembler [41]), they showed limited scalability, accuracy, and computational efficiency. Therefore, in 2015, Abu-Doleh A *et al* proposed Spaler [42] taking advantage of Spark and GraphX API. It consisted of two main parts: (a) de Bruijn graph construction, and (b) Contigs generating. It was evaluated with other MPI-based tools in terms of quality, execution time, and scalability. Experimental results [42] showed that Spaler had better scalability and it could achieve comparable or better assemble quality.

To resolve the large memory requirement problem of most OLC *de novo* assemblers, in 2017, Paul A *et al* [43] employed string graph reduction algorithms taking advantage of Spark. The proposed Spark algorithms were evaluated with a very large sample dataset. The results showed that this dataset was assembled by the proposed Spark algorithms using 15 virtual machines in 0.5 hours compared to the 7.5 hours of OLC based Omega [44] assembler.

In addition, some new assembly algorithms have also been proposed based on the Spark platform itself.

In 2016, Pan X *et al* [45] put forward a new assembling algorithm based on Spark which employed the method of matching K-2 bit to simplify the de Bruijn graph. This algorithm was evaluated using 6 groups of DNA in the NCBI. Experimental results [45] showed that this strategy not only solved the problem of low efficiency based on the MapReduce algorithm, but also optimized the algorithm itself. The combination of these two aspects were very suitable for the large-scale DNA sequence

assembling. Moreover, the results also showed that the new sequence assembling algorithm based on Spark could ensure accuracy of assembling results.

To address the problem of poor assembling precision and low efficiency, in 2017, Dong G *et al* [46] proposed SA-BR-Spark, a new sequence assembly algorithm based on Spark. The authors first designed a precise assembling algorithm under the strategy of finding the source of reads based on the MapReduce and Eulerian path algorithm (SA-BR-MR). SA-BR-MR calculated 54 sequences which were randomly selected from animals, plants and microorganisms with base lengths from hundreds to tens of thousands from NCBI. All matching rates of 54 sequences were 100%. For each species, the algorithm also summarized the range of K which made the matching rates to be 100%. In order to verify the range of K value of hepatitis C virus (HCV) and related variants, the randomly selected eight HCV variants were calculated. The results confirmed the correctness of K range of hepatitis C and related variants from NCBI. After that, SA-BR-Spark was put forward. Experimental results [46] showed that SA-BR-Spark provided a superior computational speed compared with SA-BR-MR.

SPARK IN SEQUENCE ANALYSIS

Spark in variant analysis

The GATK (Genome Analysis Toolkit) DNA analysis pipeline is widely used in genomic data analysis. Before Spark-based GATK tools were created, while several other tools had been developed to address the issue of scalability in the pipeline (such as Halvade [18] and Churchill [47]), they showed limited scalability, accuracy and computational efficiency.

Therefore, in 2015, Mushtaq H *et al* [48] utilized Spark to propose a cluster-based GATK pipeline. To reduce the execution time, this approach kept data active in the memory between the map and reduce phases. By using runtime statistics of the active workload, it achieved a dynamic load balancing algorithm that could better utilize system performance. Experimental results [48] showed that this method achieved a 4.5x speedup compared to the multi-threaded GATK pipeline on a single node. In addition, when executed on a 4-node cluster, this approach was 63% faster than Halvade.

After that, in 2016, Deng L *et al* proposed HiGene [49], which employed Spark to enable multi-core and multi-node parallelization of the GATK pipeline. HiGene put forward a dynamic computing resource scheduler and an efficient data skew mitigation method to improve performance. Experiments were conducted with the NA12878 whole human genome dataset. The results [49] showed that HiGene reduced the total running time from days to nearly an hour. Furthermore, compared with Halvade, HiGene was also 2x faster. Meanwhile, Li X *et al* employed Spark to propose GATK-Spark [50] to parallel the GATK pipeline by taking full account of compute,

workload and I/O characteristics. It was built on top of ADAM format [51]. Experimental results [50] showed that GATK-Spark shortened the total running time from 20 hours to 30 minutes on 256 CPU cores which achieved more than 37 times speedup.

The advent of Spark provides the possibility of interactive processing for NGS data. In 2014, Wiewiórka M *et al* proposed SparkSeq [52] to build and run genomic analysis pipelines in an interactive way by using Spark. Experimental results showed that SparkSeq achieved 8.4–9.15 times speedup than SeqPig. Moreover, it could accelerate data querying up to 110x and reduce memory consumption by 13x.

Spark in motif analysis

Due to the nature of NGS technology, the generated data are usually accompanied by some noise or other types of errors which are known as uncertain data [53]. Among these uncertain data, there are some frequently recurring motifs, also known as transcription factor binding sites (TFBS) [54]. Mining motifs from these uncertain data is an important problem but a computationally intensive task. Before Spark-based mining algorithm was created, while several mining algorithms had been developed (such as HPSPM [55], DGSP [56] and SPAMC [57]), they showed limited scalability. Therefore, Jiang F *et al* [58] utilized Spark to propose a scalable algorithm for mining sequence motifs. This algorithm took advantage of Spark's RDDs and DAG, and allowed users to specify the minimum and maximum length of motif. Experiments were conducted with human genome datasets and bacteria DNA sequence datasets. The results [58] showed this approach could take a short period of time to extract accurate motifs.

SPARK IN OTHER BIOLOGICAL APPLICATIONS

Spark in population genomic inference

Efficient score statistic methods [59] are widely applied in high-throughput population genomic data inference. A typical method of estimating the sampling distribution of the statistics is to employ asymptotic approximation, but it is inappropriate for small or uncommon variants. Although resampling methods [60] are appropriate for the inference in population genomics context, they greatly increase the computational burden of analysis. In order to tackle the computational challenge for resampling based inference, Bahmani A *et al* proposed SparkScore [61], a distributed population genomic inference approach taking advantage of Spark. SparkScore leveraged the nature of asymptotic and resampling inference based on efficient score statistics. Experiments on synthetic datasets using Amazon Elastic MapReduce (EMR) [61] demonstrated the efficiency and scalability of SparkScore.

Spark in epigenetics

CpG islands (CGI) are important epigenetic markers, which play an essential role in epigenetics [62]. However, it is very challenging to investigate the CpG islands and their structures. Before Spark-based applications were developed, while several methods had been proposed to determine the CPG island (such as bisulfite modification-based methods), they were time-consuming and too costly. Thus, Yu N *et al* [63] utilized Spark to propose a novel CpG box model and a Markov model to redefine and investigate the CpG island which could greatly accelerate the analytic process. Experiments were conducted with Human and mouse chromosome sequences, 24 chromosomes and 21 chromosomes. The results [63] showed this cloud-assisted method displayed considerable accuracy and faster processing power (6-7 times faster with 10 cores) compared with sequential processing.

Spark in phylogeny

Phylogeny reconstruction plays an important role in molecular evolutionary studies but faces significant computational challenges. Before Spark-based tools were created, while several tools had been put forward for phylogeny reconstruction, they could not scale well with a significant increase in data sets. Therefore, in 2016, Xu X *et al* proposed CloudPhylo [64], a fast and scalable Phylogeny reconstruction tool making use of Spark. It evenly distributed the entire computational workload among the working nodes. Experiment was conducted with the 5220 bacteria whole genome DNA sequences. The results [64] showed that CloudPhylo took 24508 seconds with one worker node and it could scale well as worker nodes increased. Moreover, CloudPhylo performed better than several existing tools when using more worker nodes. In addition, CloudPhylo achieved higher speedup on a larger dataset of about 100GB generated by simulation.

Spark in drug discovery

It is crucial to identify candidate molecules that affect disease-related proteins in drug discovery. Although the Chemogenomics project tries to identify candidate molecules using machine learning predictor programs [65-67], these programs spend a significant time and cannot be easily extended to multiple nodes. To migrate existing programs to multi-node clusters without changing the original programs, Harnie D *et al* proposed S-CHEMO [68] using Spark. In S-CHEMO, the intermediate data would be consumed again immediately on nodes that generated the data, reducing time and network bandwidth consumption. Experiments [68] compared S-CHEMO with the original pipeline, which showed almost linear speedup up to 8 nodes. Moreover, this implementation also allowed easier monitoring.

Spark in Single-cell RNA sequencing

Single-cell RNA sequencing (scRNA-seq) is crucial for understanding biological processes. Compared with standard bulk RNA-seq experiments, scRNA-seq experiments typically generate a greater number of cell profiles. Although there are already several RNA-seq processing pipelines (such as Halvade, SparkSeq and SparkBWA), they cannot process such a large number of profiles. Therefore, Falco [69] was created to process large-scale transcriptomic data in parallel by using Hadoop and Spark. Experiments were conducted with two public scRNA-seq datasets. The results [69] showed compared with a highly optimized single-node analysis, Falco was at least 2.6 times faster. Moreover, as the number of computing nodes increased, running time decreased. Furthermore, it allowed users to employ the low-cost spot instances of AWS which reduced the cost of analysis by 65%.

Spark in variant association and population genetics studies

Effectively analyzing thousands of individuals and millions of variants is a computationally intensive problem. Traditional parallel strategies such as MPI/OpenMP show poor scalability. While Hadoop provides an efficient and scalable computing framework, it is heavily dependent on disk operations. Therefore, in 2015, O'Brien A *et al* proposed VariantSpark [70] to parallel population-scale tasks based on Spark and associated machine learning library, MLlib. Experiments were conducted on 3000 individuals with 80 million variants, which showed that VariantSpark was 80% faster than ADAM, Hadoop/Mahout implementation and ADMIXTURE [71]. Moreover, compared with R and Python implementations it was more than 90 % faster. And in 2017, Di Z *et al* proposed SEQSpark [72] to perform rare variant association analysis by using Spark. It was evaluated with whole-genome and simulated exome sequence data. The former was completed in 1.5 hours and the latter in 1.75 hours. Moreover, it was always faster than Variant Association Tools and PLINK/SEQ, and in some cases running time was reduced to one percent.

Spark in other works

Biological simulations and experiments produce a large number of numerical datasets, and in 2017 Klein M *et al* proposed Biospark [73] to process these data. Biospark was based on Hadoop and Spark, consisting of a set of Java, C++ and Python libraries. In addition, it provided the abstractions for parallel analysis of standard data types, including multidimensional arrays and images. To help parallel analysis of some common datasets, it also provided APIs and file conversion tools, including Monte Carlo, molecular dynamics simulations and time-lapse microscopy.

DISCUSSION

Spark is an in-memory iterative computing framework designed for large-scale data processing. It is suitable for applications that require iterative operations on specific datasets. The greater the

amount of data, the higher the computational intensity and the greater the benefit. When the data volume is small but the computational intensity is large, the benefit is relatively small. In addition, Spark is also suitable for applications where the amount of data is not particularly large but real-time statistical analyses are required.

However, due to the nature of RDD, Spark is not suitable for applications that require asynchronous fine-grained update in execution, such as web service storage or incremental web crawlers and indexes. In addition, we need to consider the potential complexity of creating and maintaining a Spark cluster. Moreover, when Spark runs on a commercial cloud computing platform such as AWS, there is a certain delay in the transmission of large-scale datasets over the Internet. This issue does not exist when Spark runs on a local computer cluster. Furthermore, we need to learn a new API and perhaps even language (especially given the functional programing nature of the API).

Although Spark has been applied in some areas of bioinformatics and has achieved good results, other areas have not yet been involved, such as proteomics, biomedical text, and metabolomics. Moreover, as cloud computing and some web servers become more and more available, some issues must be considered, such as the time cost of large amounts of input data from local to remote servers in slow networks, cloud computing fees, data security and privacy.

Table 1 summarizes the bioinformatics tools and algorithms based on Apache Spark.

CONCLUSION

With the rapid development of NGS technology, a large number of genomic data have been generated, which poses a great challenge to traditional bioinformatics tools. For this reason, we have summarized the relevant works about Spark in bioinformatics and made a guideline on this topic. First, we make a comparison between Spark and Hadoop, and then outline the Spark cluster architecture, programming model, and processing mechanism. After that, we survey Spark-based applications in the NGS and other biological domains. A researcher who wants to get involved in this field can have a general understanding of Spark in bioinformatics through our survey.

In summary, Spark is a fast and general-purpose computing framework designed for large-scale data processing. It ensures high fault tolerance and high scalability by introducing the RDD abstraction and DAG scheduling. We believe that bioinformatics applications based on Spark will provide promising performance for biological researchers in the future.

Key Points

- Apache Spark not only gives researchers a possibility of achieving efficient, scalable and fault-tolerant computing performance, but also supports various system workloads such as batch processing, iterative, interactive and stream computing.
- We outline the Apache Spark framework to help researchers to understand its architecture, programming model and processing mechanism.
- We present Spark-based applications that can be employed in bioinformatics and discuss the strengths and weaknesses of Spark and the challenges faced in this field.

COMPETING INTERESTS

The authors declare that they have no competing interests.

FUNDING

This work was supported by National Key R&D Program of China [grant numbers 2017YFB0202600, 2016YFC1302500, 2016YFB0200400 and 2017YFB0202104]; National Natural Science Foundation of China [grant numbers 61772543, U1435222, 61625202, 61272056 and 61771331]; and Guangdong Provincial Department of Science and Technology [grant number 2016B090918122].

REFERENCES

1. Dean J, Ghemawat S. **MapReduce: simplified data processing on large clusters**. *Communications of the ACM* 2008, **51**(1):107-113.
2. Zou Q, Li X-B, Jiang W-R *et al*. **Survey of MapReduce frame operation in bioinformatics**. *Briefings in bioinformatics* 2013, **15**(4):637-647.
3. Zou Q, Hu Q, Guo M *et al*. **HAlign: Fast multiple similar DNA/RNA sequence alignment based on the centre star strategy**. *Bioinformatics* 2015, **31**(15):2475-2481.
4. Nguyen T, Shi W, Ruden D. **CloudAligner: A fast and full-featured MapReduce based tool for sequence mapping**. *BMC research notes* 2011, **4**(1):171.
5. Nordberg H, Bhatia K, Wang K *et al*. **BioPig: a Hadoop-based analytic toolkit for large-scale sequence data**. *Bioinformatics* 2013, **29**(23):3014-3019.
6. Zaharia M, Chowdhury M, Franklin MJ *et al*. **Spark: Cluster computing with working sets**. *HotCloud* 2010, **10**(10-10):95.
7. Han Z, Zhang Y. **Spark: A Big Data Processing Platform Based on Memory Computing**. In: *Seventh International Symposium on Parallel Architectures, Algorithms and Programming: 2016*. 172-176.

8. Zaharia M, Chowdhury M, Das T *et al*. **Resilient distributed datasets: A fault-tolerant abstraction for in-memory cluster computing**. In: *Proceedings of the 9th USENIX conference on Networked Systems Design and Implementation: 2012*. USENIX Association: 2-2.

9. Convolbo MW, Chou J. **Cost-aware DAG scheduling algorithms for minimizing execution cost on cloud resources**. *Journal of Supercomputing* 2016, **72**(3):985-1012.

10. Smith TF, Waterman MS. **Identification of common molecular subsequences**. *Journal of Molecular Biology* 1981, **147**(1):195-197.

11. Zhao G, Ling C, Sun D. **SparkSW: Scalable Distributed Computing System for Large-Scale Biological Sequence Alignment**. In: *Ieee/acm International Symposium on Cluster, Cloud and Grid Computing: 2015*. 845-852.

12. Xu B, Li C, Zhuang H *et al*. **DSA: Scalable Distributed Sequence Alignment System Using SIMD Instructions**. In: *Ieee/acm International Symposium on Cluster, Cloud and Grid Computing: 2017*. 758-761.

13. Xu B, Li C, Zhuang H *et al*. **Efficient Distributed Smith-Waterman Algorithm Based on Apache Spark**. In: *IEEE International Conference on Cloud Computing: 2017*. 608-615.

14. Li H, Durbin R. **Fast and accurate short read alignment with Burrows–Wheeler transform**: Oxford University Press; 2009.

15. Li H, Durbin R. **Fast and accurate long-read alignment with Burrows–Wheeler transform**. *Bioinformatics* 2010, **26**(5):589-595.

16. Li H. **Aligning sequence reads, clone sequences and assembly contigs with BWA-MEM**. 2013, **1303**.

17. Abuín JM, Pichel JC, Pena TF *et al*. **BigBWA: approaching the Burrows–Wheeler aligner to Big Data technologies**. *Bioinformatics* 2015, **31**(24):4003.

18. Decap D, Reumers J, Herzeel C *et al*. **Halvade: scalable sequence analysis with MapReduce**. *Bioinformatics* 2015, **31**(15):2482-2488.

19. Pireddu L, Leo S, Zanetti G. **SEAL: a distributed short read mapping and duplicate removal tool**. *Bioinformatics* 2011, **27**(15):2159.

20. Al-Ars Z, Mushtaq H. **Scalability Potential of BWA DNA Mapping Algorithm on Apache Spark**. In: *SIMBig: 2015*. 85-88.

21. Abuín JM, Pichel JC, Pena TF *et al*. **SparkBWA: Speeding Up the Alignment of High-Throughput DNA Sequencing Data**. *Plos One* 2016, **11**(5):e0155461.

22. Alars HMA. **Streaming Distributed DNA Sequence Alignment Using Apache Spark**. 2017.

23. Mirarab S, Nguyen N, Warnow T. **PASTA: ultra-large multiple sequence alignment**. In: *International Conference on Research in Computational Molecular Biology: 2014*. Springer: 177-191.

24. Liu K, Warnow TJ, Holder MT *et al*. **SATe-II: very fast and accurate simultaneous estimation of multiple sequence alignments and phylogenetic trees**. *Systematic biology* 2011, **61**(1):90-106.

25. Abuín JM, Pena TF, Pichel JC. **PASTASpark: multiple sequence alignment meets Big Data**. *Bioinformatics* 2017, **33**(18):2948-2950.

26. Miyazawa S. **A reliable sequence alignment method based on probabilities of residue correspondences**. *Protein Engineering* 1995, **8**(10):999.

27. Katoh K, Standley DM. **MAFFT Multiple Sequence Alignment Software Version 7: Improvements in Performance and Usability**. *Molecular Biology & Evolution* 2013, **30**(4):772-780.

28. Do CB, Mahabhashyam MS, Brudno M *et al*. **ProbCons: Probabilistic consistency-based multiple sequence alignment**. *Genome Research* 2005, **15**(2):330.

29. Tommaso PD, Moretti S, Xenarios I *et al*. **T-Coffee: a web server for the multiple sequence alignment of protein and RNA sequences using structural information and homology extension**. *Nucleic Acids Research* 2011, **39**(Web Server issue):13-17.

30. Lladós J, Guirado F, Cores F *et al*. **PPCAS: Implementation of a Probabilistic Pairwise Model for Consistency-Based Multiple Alignment in Apache Spark**; 2017.

31. Altschul S, Gish W, Miller W *et al*. **Basic local alignment search tool. J. Mol. Biol**. 1990.

32. C C, G C, V A *et al*: **BLAST+: architecture and applications**. *Bmc Bioinformatics* 2009, **10**(1):421.

33. Darling AE, Carey L, Feng WC. **The design, implementation, and evaluation of mpiBLAST**. In.: Los Alamos National Laboratory; 2003.

34. Vouzis PD, Sahinidis NV. **GPU-BLAST: using graphics processors to accelerate protein sequence alignment**. *Bioinformatics* 2010, **27**(2):182-188.

35. Matsunaga A, Tsugawa M, Fortes J. **Cloudblast: Combining mapreduce and virtualization on distributed resources for bioinformatics applications**. In: *eScience, 2008 eScience'08 IEEE Fourth International Conference on: 2008*. IEEE: 222-229.

36. Castro MRD, Tostes CDS, Dávila AMR *et al*. **SparkBLAST: scalable BLAST processing using in-memory operations**. *Bmc Bioinformatics* 2017, **18**(1):318.

37. Zhou W, Li R, Yuan S *et al*. **MetaSpark: a spark-based distributed processing tool to recruit metagenomic reads to reference genomes**. *Bioinformatics* 2017, **33**(7):1090-1092.

38. Niu B, Zhu Z, Fu L *et al*. **FR-HIT, a very fast program to recruit metagenomic reads to homologous reference genomes**. *Bioinformatics* 2011, **27**(12):1704-1705.

39. Boisvert S, Laviolette F, Corbeil J. **Ray: simultaneous assembly of reads from a mix of high-throughput sequencing technologies**. *Journal of Computational Biology A Journal of Computational Molecular Cell Biology* 2010, **17**(11):1519.

40. Simpson JT, Wong K, Jackman SD *et al*. **ABySS: a parallel assembler for short read sequence data**. *Genome Research* 2009, **19**(6):1117.

41. Meng J, Wang B, Wei Y *et al*. **SWAP-Assembler: scalable and efficient genome assembly towards thousands of cores**. *Bmc Bioinformatics* 2014, **15**(S9):S2.

42. Abu-Doleh A, Çatalyürek ÜV. **Spaler: Spark and GraphX based de novo genome assembler**. In: *IEEE International Conference on Big Data: 2015*. 1013-1018.

43. Paul AJ, Lawrence D, Ahn TH. **Overlap Graph Reduction for Genome Assembly using Apache Spark**. In: *The ACM International Conference: 2017*. 613-613.

44. Haider B, Ahn TH, Bushnell B *et al*. **Omega: an Overlap-graph de novo Assembler for Metagenomics**. *Bioinformatics* 2014, **30**(19):2717-2722.

45. Pan X, Fu X-L, Dong G-F *et al*. **DNA sequence splicing algorithm based on Spark**. In: *Industrial Informatics-Computing Technology, Intelligent Technology, Industrial Information Integration (ICIICII), 2016 International Conference on: 2016*. IEEE: 52-56.

46. Dong G, Fu X, Li H *et al*. **An Accurate Sequence Assembly Algorithm for Livestock, Plants and Microorganism Based on Spark**. *International Journal of Pattern Recognition & Artificial Intelligence* 2017, **31**(8).

47. Kelly BJ, Fitch JR, Hu Y *et al*. **Churchill: an ultra-fast, deterministic, highly scalable and balanced parallelization strategy for the discovery of human genetic variation in clinical and population-scale genomics**. *Genome biology* 2015, **16**(1):6.

48. Mushtaq H, Al-Ars Z. **Cluster-based Apache Spark implementation of the GATK DNA analysis pipeline**. In: *Bioinformatics and Biomedicine (BIBM), 2015 IEEE International Conference on: 2015*. IEEE: 1471-1477.

49. Deng L, Huang G, Zhuang Y *et al*. **HiGene: A high-performance platform for genomic data analysis**. In: *IEEE International Conference on Bioinformatics and Biomedicine: 2016*. 576-583.

50. Li X, Tan G, Zhang C *et al*. **Accelerating large-scale genomic analysis with Spark**. In: *Bioinformatics and Biomedicine (BIBM), 2016 IEEE International Conference on: 2016*. IEEE: 747-751.

51. Massie M, Nothaft F, Hartl C *et al*. **Adam: Genomics formats and processing patterns for cloud scale computing**. *EECS Department, University of California, Berkeley, Tech Rep UCB/EECS-2013-207* 2013.

52. Wiewiórka MS, Messina A, Pacholewska A *et al*. **SparkSeq: fast, scalable and cloud-ready tool for the interactive genomic data analysis with nucleotide precision**. *Bioinformatics* 2014, **30**(18):2652-2653.

53. Leung CK-S. **Uncertain frequent pattern mining**. In: *Frequent pattern mining.* Springer; 2014: 339-367.

54. Das MK, Dai H-K. **A survey of DNA motif finding algorithms**. *BMC bioinformatics* 2007, **8**(7):S21.

55. Shintani T, Kitsuregawa M. **Mining algorithms for sequential patterns in parallel: Hash based approach**. In: *Pacific-Asia Conference on Knowledge Discovery and Data Mining: 1998*. Springer: 283-294.

56. Qiao S, Tang C, Dai S *et al*. **Partspan: Parallel sequence mining of trajectory patterns**. In: *Fuzzy Systems and Knowledge Discovery, 2008 FSKD'08 Fifth International Conference on: 2008*. IEEE: 363-367.

57. Chen C-C, Tseng C-Y, Chen M-S. **Highly scalable sequential pattern mining based on mapreduce model on the cloud**. In: *Big Data (BigData Congress), 2013 IEEE International Congress on: 2013*. IEEE: 310-317.

58. Jiang F, Leung CK, Sarumi OA *et al*. **Mining sequential patterns from uncertain big DNA in the spark framework**. In: *Bioinformatics and Biomedicine (BIBM), 2016 IEEE International Conference on: 2016*. IEEE: 874-881.

59. Rao CR. **Large sample tests of statistical hypotheses concerning several parameters with applications to problems of estimation**. In: *Mathematical Proceedings of the Cambridge Philosophical Society: 1948*. Cambridge University Press: 50-57.

60. Westfall PH, Young SS. **Resampling-based multiple testing: Examples and methods for p-value adjustment**, vol. 279: John Wiley & Sons; 1993.

61. Bahmani A, Sibley AB, Parsian M *et al*. **SparkScore: leveraging apache spark for distributed genomic inference**. In: *Parallel and Distributed Processing Symposium Workshops, 2016 IEEE International: 2016*. IEEE: 435-442.

62. Erkek S, Hisano M, Liang C-Y *et al*. **Molecular determinants of nucleosome retention at CpG-rich sequences in mouse spermatozoa**. *Nature structural & molecular biology* 2013, **20**(7):868-875.

63. Yu N, Li B, Pan Y. **A cloud-assisted application over apache spark for investigating epigenetic markers on DNA genome sequences**. In: *Big Data and Cloud Computing (BDCloud), Social Computing and Networking (SocialCom), Sustainable Computing and Communications (SustainCom)(BDCloud-SocialCom-SustainCom), 2016 IEEE International Conferences on: 2016*. IEEE: 67-74.

64. Xu X, Ji Z, Zhang Z. **CloudPhylo: a fast and scalable tool for phylogeny reconstruction**. *Bioinformatics* 2016, **33**(3):438-440.

65. Wale N. **Machine learning in drug discovery and development**. *Drug Development Research* 2011, **72**(1):112-119.

66. Costello JC, Heiser LM, Georgii E *et al*. **A community effort to assess and improve drug sensitivity prediction algorithms**. *Nature biotechnology* 2014, **32**(12):1202-1212.

67. Sastry GM, Inakollu VS, Sherman W. **Boosting virtual screening enrichments with data fusion: coalescing hits from two-dimensional fingerprints, shape, and docking**. *Journal of chemical information and modeling* 2013, **53**(7):1531-1542.

68. Harnie D, Saey M, Vapirev AE *et al*. **Scaling machine learning for target prediction in drug discovery using apache spark**. *Future Generation Computer Systems* 2017, **67**:409-417.

69. Yang A, Troup M, Lin P *et al*. **Falco: a quick and flexible single-cell RNA-seq processing framework on the cloud**. *Bioinformatics* 2016, **33**(5):767-769.

70. O'Brien AR, Saunders NFW, Guo Y *et al*. **VariantSpark: population scale clustering of genotype information**. *Bmc Genomics* 2015, **16**(1):1-9.

71. Alexander DH, Novembre J, Lange K. **Fast model-based estimation of ancestry in unrelated individuals**. *Genome Research* 2009, **19**(9):1655.

72. Di Z, Zhao L, Li B *et al*. **SEQSpark: A Complete Analysis Tool for Large-Scale Rare Variant Association Studies Using Whole-Genome and Exome Sequence Data**. *American Journal of Human Genetics* 2017, **101**(1):115.

73. Klein M, Sharma R, Bohrer CH *et al*. **Biospark: scalable analysis of large numerical datasets from biological simulations and experiments using Hadoop and Spark**. *Bioinformatics* 2017, **33**(2):303-305.

Table 1 Bioinformatics tools and algorithms based on Apache Spark

Name	Function	Features	Pros/Cons	Reference
SparkSW	Alignment and mapping	Consists of three phases: data preprocessing, SW as map tasks and top K records as reduce tasks	Load-balancing, scalable, but without the mapping location and traceback of optimal alignment	[11]
DSA	Alignment and mapping	Leverages data parallel strategy based on SIMD instruction	Up to 201x speedup over SparkSW and almost linear speedup with the increase of cluster nodes	[12]
CloudSW	Alignment and mapping	Leverages SIMD instruction and provides APIs service in cloud	Up to 3.29x speedup over DSA and 621x speedup over SparkSW, high scalability and efficiency	[13]
SparkBWA	Alignment and mapping	Consists of three main stages: RDDs creation, map, and reduce phases, employs two independent software layers	For shorter reads, average 1.9x and 1.4x faster than SEAL and pBWA. For longer reads, average 1.4x faster than BigBWA and Halvade, but requires the data availability in HDFS	[21]
StreamBWA	Alignment and mapping	The input data are being streamed into the cluster directly from a compressed file	~2x faster than non-streaming approach, and 5x faster than SparkBWA	[22]
PASTASpark	Alignment and mapping	Employs an in-memory RDD of key-value pairs to parallel the calculating MSAs phase	Up to 10x speedup than single-threaded PASTA, ensures scalability and fault tolerance	[25]
PPCAS	Alignment and mapping	Based on the MapReduce processing paradigm in Spark	Better with single node and shows almost linear speedup with the increase of nodes	[30]
SparkBLAST	Alignment and mapping	Utilizes pipe operator and RDDs of Spark to call BLAST as an external library	Outperforms CloudBLAST in terms of speedup, scalability and efficiency	[36]
MetaSpark	Alignment and mapping	Consists of five steps: constructing k-mer RefindexRDD, constructing k-mer ReadlistRDD, seeding, filtering, and banded alignment	Recruits significantly more reads than SOAP2, BWA and LAST, and more reads by ~4 than FR-HIT, and shows good scalability and overall high performance	[37]
Spaler	Assembly	Employs GraphX API of Spark, consists of two main parts: de Bruijn graph construction and contigs generating	Shows better scalability and achieves comparable or better assemble quality than ABySS, Ray, and SWAP-Assembler	[42]
SA-BR-Spark	Assembly	Under the strategy of finding the source of reads based Spark platform	Shows a superior computational speed than SA-BR-MR	[46]
HiGene	Variant analysis	Puts forward a dynamic computing resource scheduler and an efficient data skew mitigation way	Reduces total running time from days to nearly an hour, and 2x faster than Halvade	[49]

GATK-Spark	Variant analysis	Takes full account of compute, workload, and I/O characteristics	Achieves more than 37 times speedup	[50]
SparkSeq	Variant analysis	Builds and runs genomic analysis pipelines in an interactive way by using Spark	Achieves 8.4-9.15 times speedup than SeqPig, and accelerate data querying up to 110x and reduce memory consumption by 13x	[52]
SparkScore	Population genomic inference	Employs asymptotic and resampling inference on the basis of efficient score statistics	Shows good efficiency and scalability with EMR on synthetic datasets with EMR	[61]
CloudPhylo	Phylogeny	Evenly distributes the entire workloads among the worker nodes	Shows good scalability and high efficiency, and Spark version is better than Hadoop version	[64]
S-CHEMO	Drug discovery	Intermediate data is immediately consumed again on the nodes that produce, saving time and bandwidth	Shows almost linear speedup up to 8 nodes compared with the original pipeline	[68]
Falco	Single-cell RNA sequencing	Consist of a splitting step, an optional pre-processing step and the main analysis step	At least 2.6x faster than a highly optimized single-node analysis, and with the increase of nodes, running time decreases	[69]
VariantSpark	Variant association and population genetics studies	Parallels population-scale tasks based on Spark and associated MLlib	80% faster than ADAM, Hadoop/Mahout version and ADMIXTURE, and more than 90% faster than R and Python implementations	[70]
SEQSpark	Variant association and population genetics studies	Splits large-scale datasets into many small blocks to perform rare variant association analysis	Always faster than Variant Association Tools and PLINK/SEQ, and in some cases, running time is reduced to one percent	[72]
BioSpark	data-parallel analysis on large numerical datasets	Consists of a set of Java, C++ and Python libraries, abstractions for parallel analysis of standard data types, some APIs and file conversion tools	Convenient, scalable, and useful, brings domain-specific features for biology	[73]

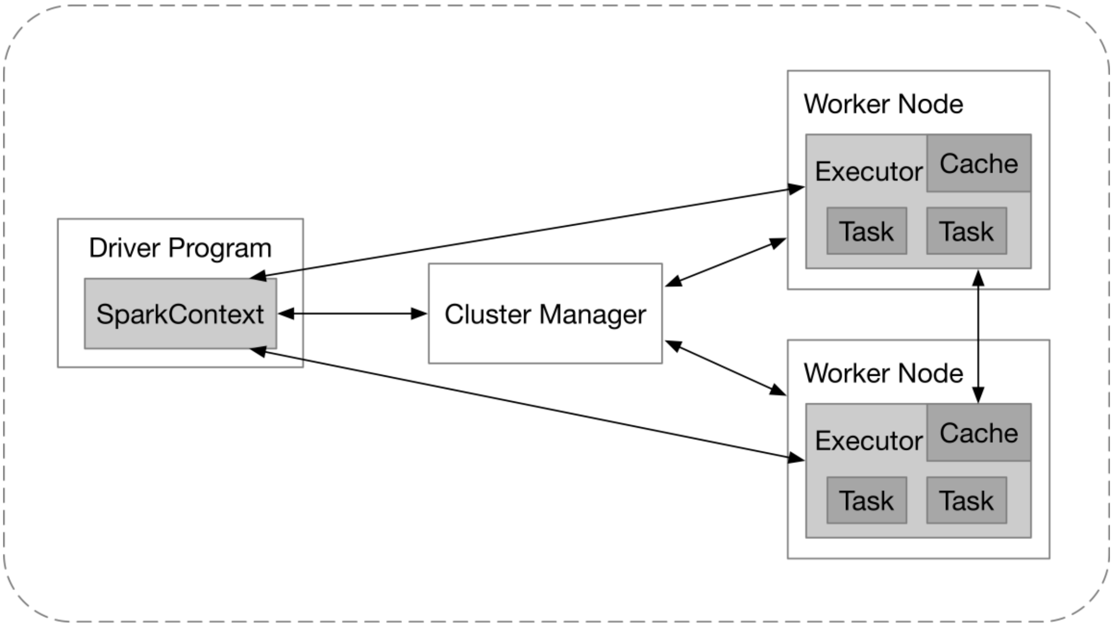

Figure 1: The cluster architecture of Spark

 Click here to download Figure Figure 2.docx

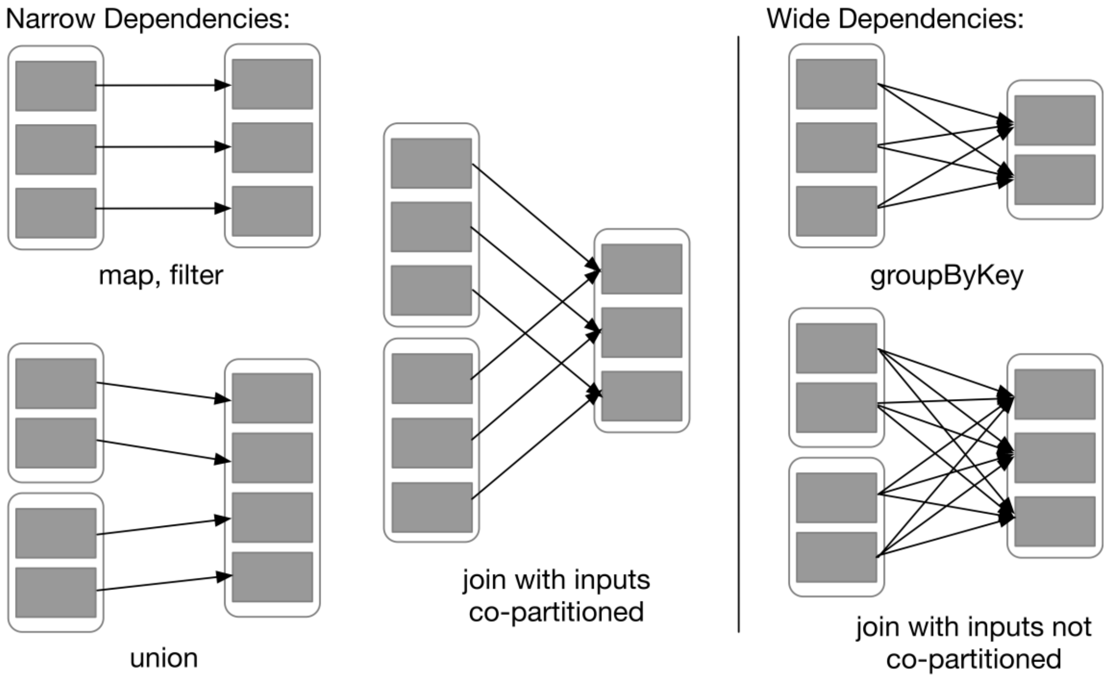

Figure 2: Examples of narrow and wide dependencies. Each box is an RDD, with partitions shown as shaded rectangles.

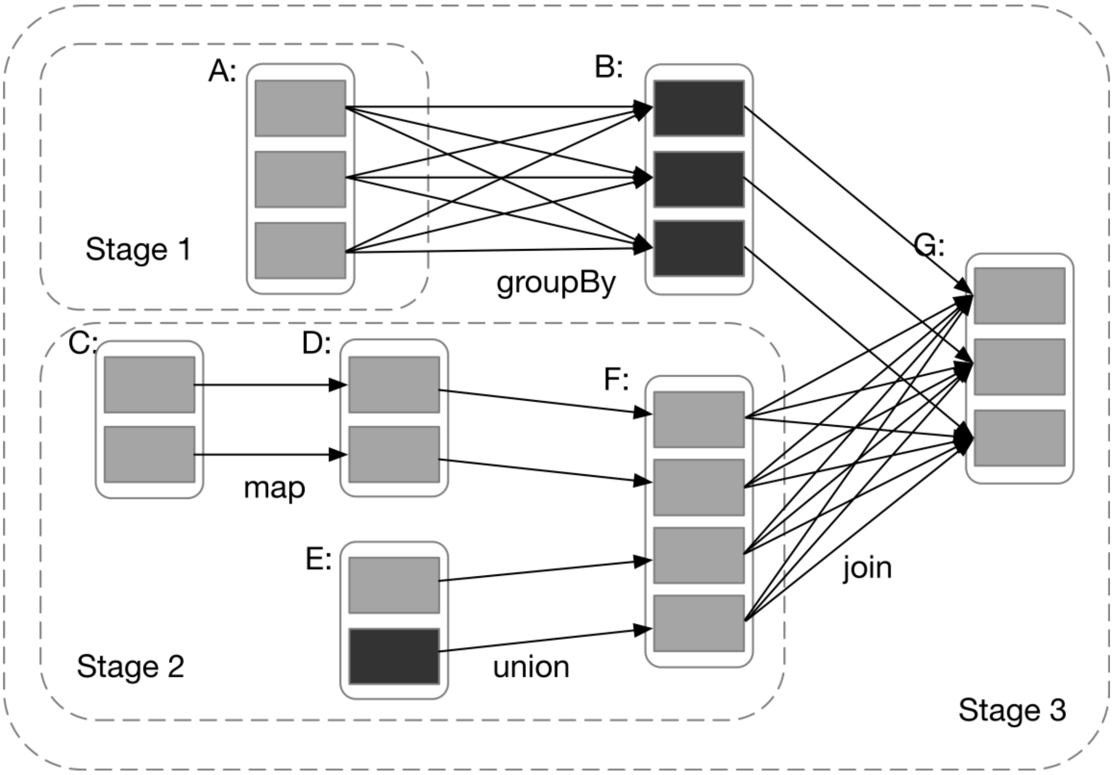

Figure 3: Example of how Spark computes job stages. Boxes with solid outlines are RDDs. Partitions are shaded rectangles, in black if they are already in memory. To run an action on RDD G, we build stages at wide dependencies and pipeline narrow transformation inside each stage. In this case, stage 1's output RDD is already in RAM, so we run stage 2 and then stage 3.

Click here to access/download
Supplementary Material
Cover Letter.docx

GitHub wiki link

Click here to access/download
Supplementary Material
Home.md
