# Supplementary material for: Bioinformatics applications on Apache Spark
Source: Gigascience. 2018 Aug 7;7(8):giy098. doi: 10.1093/gigascience/giy098 (PMC6113509; doi:10.1093/gigascience/giy098)
Supplement: GIGA-D-18-00131_Revision_3.pdf [file giy098_giga-d-18-00131_revision_3.pdf]

GigaScience

Bioinformatics applications on Apache Spark

--Manuscript Draft--

**Manuscript Number:**	GIGA-D-18-00131R3
**Full Title:**	Bioinformatics applications on Apache Spark
**Article Type:**	Review
**Funding Information:**	National Key R&D Program of China (2017YFB0202600, 2016YFC1302500, 2016YFB0200400 and 2017YFB0202104) — Professor shaoliang peng<br>National Natural Science Foundation of China (61772543, U1435222, 61625202, 61272056 and 61771331) — Professor shaoliang peng<br>Guangdong Provincial Department of Science and Technology (2016B090918122) — Professor shaoliang peng
**Abstract:**	With the rapid development of next-generation sequencing technology, ever-increasing quantities of genomic data pose a tremendous challenge to data processing. Therefore, there is an urgent need for highly scalable and powerful computational systems. Among the state-of–the-art parallel computing platforms, Apache Spark is a fast, general-purpose, in-memory, iterative computing framework for large-scale data processing that ensures high fault tolerance and high scalability by introducing the resilient distributed dataset abstraction. In terms of performance, Spark can be up to 100 times faster in terms of memory access, and 10 times faster in terms of disk access than Hadoop. Moreover, it provides advanced application programming interfaces in Java, Scala, Python, and R. It also supports some advanced components, including Spark SQL for structured data processing, MLlib for machine learning, GraphX for computing graphs, and Spark Streaming for stream computing. We surveyed Spark-based applications used in next-generation sequencing and other biological domains, such as epigenetics, phylogeny, and drug discovery. The results of this survey are used to provide a comprehensive guideline allowing bioinformatics researchers to apply Spark in their own fields.<br>Keywords: next-generation sequencing; bioinformatics; Apache Spark; resilient distributed dataset; memory computing
**Corresponding Author:**	runxin guo<br><br>CHINA
**Corresponding Author Secondary Information:**	
**Corresponding Author's Institution:**	
**Corresponding Author's Secondary Institution:**	
**First Author:**	runxin guo
**First Author Secondary Information:**	
**Order of Authors:**	runxin guo<br>yi zhao<br>quan zou<br>xiaodong fang<br>shaoliang peng
**Order of Authors Secondary Information:**	
**Response to Reviewers:**	Dear Editor and Reviewers:<br>Thank you for your letter and for the reviewers’ comments concerning our manuscript

Powered by Editorial Manager® and ProduXion Manager® from Aries Systems Corporation

	entitled "Bioinformatics Application on Apache Spark" (GIGAD1800131R2). Those comments are all valuable and very helpful for revising and improving our paper, as well as the important guiding significance to our research. We have studied the comments carefully and have made correction which we hope meet with approval. Moreover, according to the copy edited manuscript, we have updated the paper for all requested edits.<br>Yours<br>Sincerely<br>Runxin Guo, Yi Zhao, Quan Zou, Xiaodong Fang, Shaoliang Peng
**Additional Information:**	
**Question**	**Response**
Are you submitting this manuscript to a special series or article collection?	No
**Experimental design and statistics**<br><br>Full details of the experimental design and statistical methods used should be given in the Methods section, as detailed in our Minimum Standards Reporting Checklist. Information essential to interpreting the data presented should be made available in the figure legends.<br><br>Have you included all the information requested in your manuscript?	Yes
**Resources**<br><br>A description of all resources used, including antibodies, cell lines, animals and software tools, with enough information to allow them to be uniquely identified, should be included in the Methods section. Authors are strongly encouraged to cite Research Resource Identifiers (RRIDs) for antibodies, model organisms and tools, where possible.<br><br>Have you included the information requested as detailed in our Minimum Standards Reporting Checklist?	Yes
**Availability of data and materials**<br><br>All datasets and code on which the conclusions of the paper rely must be	Yes

either included in your submission or deposited in publicly available repositories (where available and ethically appropriate), referencing such data using a unique identifier in the references and in the "Availability of Data and Materials" section of your manuscript.<br><br>Have you have met the above requirement as detailed in our Minimum Standards Reporting Checklist?	

Manuscript

Click here to access/download;Manuscript;Bioinformatics applications on Apache Spark.docx

Click here to view linked References

Bioinformatics applications on Apache Spark

Runxin Guo[1†], Yi Zhao[2†], Quan Zou[3†], Xiaodong Fang[4*], Shaoliang Peng[1,5*]

[1]College of Computer, National University of Defense Technology, Changsha 410073, China;

[2]Institute of Computing Technology, Chinese Academy of Sciences, Beijing 100190, China;

[3]School of Computer Science and Technology, Tianjin University, Tianjin 300350, China;

[4]BGI Genomics, BGI-Shenzhen, Shenzhen 518083, China;

[5]College of Computer Science and Electronic Engineering & National Supercomputer Centre in Changsha, Hunan University, Changsha 410082, China

*Correspondence address:

Xiaodong Fang, BGI Genomics, BGI-Shenzhen, Shenzhen 518083, China; E-mail: fangxd@bgitechsolutions.com.

Shaoliang Peng, College of Computer, National University of Defense Technology, Changsha 410073, China; E-mail: pengshaoliang@nudt.edu.cn.

ORCID IDs: Runxin Guo, 0000-0002-1203-5038; Quan Zou, 0000-0001-6406-1142; Xiaodong Fang, 0000-0001-7061-3337; and Shaoliang Peng, 0000-0002-4647-2615.

[†]: Equal contributors

ABSTRACT

With the rapid development of next-generation sequencing technology, ever-increasing quantities of genomic data pose a tremendous challenge to data processing. Therefore, there is an urgent need for highly scalable and powerful computational systems. Among the state-of–the-art parallel computing platforms, Apache Spark is a fast, general-purpose, in-memory, iterative computing framework for large-scale data processing that ensures high fault tolerance and high scalability by introducing the resilient distributed dataset abstraction. In terms of performance, Spark can be up to 100 times faster in terms of memory access, and 10 times faster in terms of disk access than Hadoop. Moreover, it provides advanced application programming interfaces in Java, Scala, Python, and R. It also supports some advanced components, including Spark SQL for structured data processing, MLlib for machine learning, GraphX for computing graphs, and Spark Streaming for stream computing. We surveyed Spark-based applications used in next-generation sequencing and other biological domains, such as epigenetics, phylogeny, and drug discovery. The results of this survey

are used to provide a comprehensive guideline allowing bioinformatics researchers to apply Spark in their own fields.

Keywords: next-generation sequencing; bioinformatics; Apache Spark; resilient distributed dataset; memory computing

INTRODUCTION

Next-generation sequencing (NGS) technology has generated huge amounts of biological sequence data. To use these data efficiently, we need accurate and efficient methods of storing and analyzing such data. However, the existing bioinformatics tools cannot effectively handle such a large amount of data. Therefore, there is an urgent need for scalable and powerful distributed computing tools to solve this problem. In the field of information technology, MapReduce [1] is a distributed parallel programming model and methodology for processing large-scale datasets. It splits large-scale datasets into many key-value pairs through both the map and reduce phases, significantly improving performance and showing good scalability. By combining the Hadoop Distributed File System (HDFS) and MapReduce, Apache Hadoop can enable distributed processing of large amounts data in a reliable, efficient, and scalable way. This is in contrast to HDFS, which is mainly used for distributed storage of massive datasets, and MapReduce, which performs distributed computing on these datasets. As a result, Hadoop has been adopted by the bioinformatics community in several areas [2], including alignment [3–6], mapping [7–9], and sequence analysis [10–13].

Because of Hadoop's disk-based I/O access pattern, however, intermediate calculation results are not cached. Therefore, Hadoop is only suitable for batch data processing, and shows poor performance for iterative data processing. To resolve this problem, Apache Spark [14] has been proposed; a faster, general-purpose computing framework specifically designed to handle huge amounts of data. Unlike Hadoop's disk-based computing, Spark performs memory computing by introducing resilient distributed dataset (RDD) abstraction. Since it is possible to store intermediate results in memory, it is more efficient for iterative operations. In terms of performance, Spark can be up to 100 times faster in terms of memory access than Hadoop [14]. The gap between Spark and Hadoop is more than 10-fold greater, even if we compare between them based on disk performance [15]. In terms of flexibility, Spark provides high-level application programming interfaces (APIs) in Java, Scala, Python, and R, and interactive shell. In terms of generality, Spark provides structured data processing, machine learning, graph computing, and stream computing capabilities by supporting some advanced components.

Table 1 summarizes the bioinformatics tools and algorithms based on Apache Spark.

THE SPARK FRAMEWORK

Designed by the Algorithms, Machines and People Lab at the University of California, Berkeley, Spark is an open-source cluster computing environment designed for large-scale data processing, developed by UC Berkeley AMP lab. It provides advanced APIs in Java, Scala, Python and R, and an optimized engine that supports general execution graphs. It also supports some advanced components, including Spark SQL for structured data processing, MLlib for machine learning, GraphX for computing graphs, and Spark Streaming for stream computing.

As shown in Fig.1, each Spark application runs as an independent process on the cluster, coordinated by SparkContext in the driver program. There are two deploy modes, depending on where the driver program is running: cluster mode, and client mode. In the former mode, the driver program runs on a worker node. In the latter, the driver program runs on the client machine. First, SparkContext requests the executors on the worker nodes in the cluster from the cluster manager (either Spark's own standalone cluster manager, Apache Mesos, or Hadoop YARN). These executors are processes that can run tasks and store data in memory, or on disk for application. Next, SparkContext will send tasks to the executors to perform. Finally, the executors return the results to SparkContext after the tasks are executed. In Spark, an application generates multiple jobs. A job is split into several stages. Each stage is a task set containing several tasks, which performs calculations and produces intermediate results. A task is the smallest unit of work in Spark, completing a specific job on an executor. As for deployment of the Spark cluster, the official proposal for hardware requirements is to have 4–8 disks per node, which configure at least 8 GB of memory and 8–16 central processing unit (CPU) cores per machine, and use a 10 gigabit or higher network.

As the main abstraction in Spark, RDD is a read-only collection of objects partitioned on different nodes in the cluster so that the data in RDD can be processed in parallel. The data in RDD are stored in memory by default, but Spark automatically writes RDD data to disk if memory resources are low. RDD achieves fault tolerance through a notion of lineage [14]; that is, if an RDD partition on a node is lost because of a node failure, the RDD automatically recalculates the partition from its own data source. Moreover, Spark provides two types of operations on RDD: transformation and action. The former defines a new RDD, and the latter returns a result or writes RDD data to the storage system. Transformation employs lazy operation [16], which means that the operation of generating another RDD from one RDD transformation is not executed immediately, and the

calculation process is not actually started until an action is performed. Furthermore, each transformation operation generates a new RDD; the newly generated RDD depends on the original RDD. According to the different types of transformation operation, RDD dependencies can be divided into narrow dependency and wide dependency. The former refers to the fact that each partition in the generated RDD depends only on the parent RDD fixed partition, and the latter refers to the fact that each partition of the generated RDD depends on all partitions of the parent RDD. Fig.2 shows examples of narrow and wide dependencies. In addition, Spark also provides two extensions of RDD: DataFrame and Dataset. Spark users can seamlessly switch between these through simple API calls.

Spark also adopts a directed acyclic graph (DAG) [17] to optimize execution processes by splitting submitted jobs into several stages according to wide dependency. For narrow dependency, it divides related transformation operations into the same stage; this is because they can perform pipelining operations, and thus reduce the processing time of submitted jobs. Fig.3 shows an example of how Spark computes job stages. In addition, if the partitions on a node are lost because of node failure, Spark can utilize the DAG to recalculate the lost partitions.

SPARK IN ALIGNMENT AND MAPPING

The rapid development of NGS technology has generated a large amount of sequence data (reads), which has a tremendous impact on sequence alignment and mapping processes. Currently, sequence alignment and mapping remains time-consuming.

The Smith–Waterman (SW) algorithm [18], which produces optimal local alignment between two strings of nucleic acid sequences or protein sequences, is widely used in bioinformatics. However, this algorithm has a high computational cost because of high computational complexity. To speed up the algorithm, Zhao et al. (2015) implemented the SW algorithm on Spark for the first time, naming this SparkSW [19]. It consisted of three phases: data preprocessing, SW as map tasks, and top K records as reduce tasks. Experimental results [19] showed that SparkSW was load-balancing and scalable with increased computing resources. However, SparkSW merely supports the SW algorithm without the mapping location and traceback of optimal alignment. As a result, SparkSW executes slowly. Therefore, Xu et al. (2017) proposed DSA [20], which employed Single Instruction Multiple Data (SIMD) to parallel the sequence alignment algorithm at each worker node. Experimental results [20] showed that DSA achieved up to 201 times faster speeds over SparkSW, and almost linearly increased speed with increased cluster nodes. Subsequently, Xu et al. proposed

CloudSW [21], an efficient distributed SW algorithm that leveraged Spark and SIMD instructions to accelerate the algorithm, and provided API services in the cloud. Experimental results [21] showed that CloudSW achieved up to 3.29 times increased speed over DSA, and 621 times increased speed over SparkSW. CloudSW also showed excellent scalability and achieved speeds of up to 529 giga cell updates per second in a protein database search with 50 nodes using Aliyun cloud.

The Burrows–Wheeler aligner (BWA) is composed of BWA-backtrack [22], BWA-SW [23] and BWA-MEM [24] for performing sequence alignment and mapping in bioinformatics. Before the advent of Spark-based BWA tools, there were several other BWA tools based on big data technology, including BigBWA [25], Halvade [26] and SEAL [7]. However, these were based on Hadoop, which showed limited scalability and complex implementation. As a result, Al-Ars et al. (2015) [27] implemented three different versions of BWA-MEM and compared their performance: a native cluster-based version, a Hadoop version and a Spark version. Three implementations were evaluated on the same IBM Power7 and Intel Xeon servers, with WordCount as an example. The results [27] showed that simultaneous multithreading improved the performance of three versions of BWA-MEM, and the Spark version with 80 threads increased performance by up to 87% over the native cluster version using 16 threads. Furthermore, the four-thread Hadoop version increased performance by 17%, and the Spark version with even more threads increased performance by 27%. Then, in 2016, Abuín et al. proposed SparkBWA [28], which is composed of three main phases: the RDDs creation phase, the map phase, and the reduce phase. Experimental results [28] showed that for the BWA-backtrack algorithm, SparkBWA achieved average increased speeds of 1.9 times and 1.4 times, compared with SEAL and pBWA, respectively. For the BWA-MEM algorithm, SparkBWA was, on average, 1.4 times faster than BigBWA and Halvade tools. However, SparkBWA required significant time to preprocess the input files and finally combine the output files. Therefore, in 2017, Mushtaq et al. proposed StreamBWA [29], in which the input files were streamed into the Spark cluster. This greatly reduced the time required to preprocess data and combine the final results. Experimental results [29] showed that this streaming distributed strategy gave roughly double the speed of the non-streaming strategy. Furthermore, StreamBWA achieved a five-fold increased speed over SparkBWA.

Multiple sequence alignment (MSA) refers to the sequence alignment of three or more biological sequences, such as protein or nucleic acid sequences. One representative tool for performing MSA is PASTA [30]. PASTA is a derivative of SATé [31], which produces highly accurate alignments

in shared memory computers. However, PASTA is limited to processing small and medium datasets because the computing power of shared memory systems cannot meet the memory and time requirements of large-scale datasets. Therefore, in 2017, Abuín et al. proposed PASTASpark [32], which allowed executions on a distributed memory cluster, taking advantage of Spark. It employed an in-memory RDD of key-value pairs to parallel the calculating MSA phase. Experiments were conducted on two different clusters, Centro de Supercomputación de la Galicia (CESGA) and Amazon Web Services (AWS). The results [32] showed that PASTASpark achieved up to 10 times faster speeds than single-threaded PASTA, and was able to process 200,000 sequences in 24 hours using only AWS nodes. Therefore, PASTASpark ensured scalability and fault tolerance, which greatly reduced the time to obtain MSA.

The probabilistic pairwise model [33] is widely used in all consistency-based MSA tools, such as MAFFT [34], ProbCons [35] and T-Coffee [36]. However, global distributed memory cannot meet the demands of ever-increasing sequence datasets, which leads to the need for specialized distributed databases, such as HBase or Cassandra. As a result, Lladós et al. (2017) employed Spark to propose a new tool, PPCAS [37], which could parallel the probabilistic pairwise model for large-scale protein sequences and store it in a distributed platform. Experimental results [37] showed that it was better with a single node, and provided almost linearly increased speeds with the increased numbers of nodes. In addition, it could compute more sequences using the same amount of memory.

The National Center for Biotechnology Information's (NCBI) BLAST tool [38, 39] is widely used to implement algorithms for sequence comparison. Before the Spark-based BLAST was created, several other BLAST tools had been proposed, including mpiBLAST [40], GPU-BLAST [41] and CloudBLAST [42]. However, with the increasing amount of genomic data, these tools showed limited scalability and efficiency. As a result, Castro et al. (2017) proposed SparkBLAST [43], which utilized cloud computing and the Spark framework to parallel BLAST. In SparkBLAST, Spark's *pipe* operator and RDDs were utilized to call BLAST as an external library and perform scalable sequence alignment. It was compared with CloudBLAST on both Google and Microsoft Azure clouds. Experimental results [43] showed that SparkBLAST outperformed CloudBLAST in terms of speed, scalability, and efficiency.

Metagenomics is crucial for directly studying genetic material from environmental samples. Fragment recruitment is the process of aligning reads to reference genomes in metagenomics data analysis. In 2017, Zhou et al. proposed MetaSpark [44], which employed Spark to recruit metagenomics reads to reference genomes. MetaSpark utilized the RDD of Spark to cache datasets

in memory, and scaled well along dataset size increments. It consisted of five steps including constructing k-mer RefindexRDD, constructing k-mer ReadlistRDD, seeding, filtering, and banded alignment. It was evaluated on a 10-node cluster, working under the Spark standalone module, in which each node contained an eight-core CPU and 16 GB RAM. It employed about 1 million 75 bp Illumina read datasets, and two references: 194 human gut genomes and bacterial genomes that were 0.616 Gb and 1.3 Gb in size, respectively. Experimental results [44] showed that MetaSpark recruited more reads than FR-HIT [45] with the same parameters and 1 million reads. MetaSpark recruited 501,856 reads to 0.616 Gb human gut genome references, while FR-HIT recruited 489,638 reads. MetaSpark increased recruited reads by 2.5%. When references changed to a 1.3 Gb bacterial genome, MetaSpark recruited 463,862 reads, while FR-HIT recruited 444,671 reads. MetaSpark increased recruited reads by 4%. Moreover, the results also showed that MetaSpark offered good scalability. Under a 0.616 Gb reference, run time for 100,000 reads was 51 minutes under four nodes, and decreased slightly to 23.5 minutes under 10 nodes. For the 1 million read datasets, MetaSpark would crash under four nodes because of limited memory. Under six nodes, it finished running after 312 minutes and would sharply decrease to 201 minutes under 10 nodes.

SPARK IN ASSEMBLY

Because NGS read lengths are short (<500 bp), they must be assembled before further analysis, which is another important phase in the sequence analysis workflow. In general, there are two types of assembly: the reference assembly, and de novo assembly. The assembly algorithm includes two categories: the overlap–layout–consensus (OLC) algorithm, and the de Bruijn graph algorithm. The former is generally employed to assemble longer reads, while the latter performs well in assembling short reads.

Before Spark-based distributed memory de novo assemblers were created, although there were some MPI-based assemblers (such as Ray [46], AbySS [47] and SWAP-Assembler [48]), they showed limited scalability, accuracy, and computational efficiency. Therefore, in 2015, Abu-Doleh et al. proposed Spaler [49], taking advantage of Spark and GraphX APIs. It consisted of two main parts: de Bruijn graph construction, and contig generation. It was evaluated against other MPI-based tools in terms of quality, execution time, and scalability. Experimental results [49] showed that Spaler had better scalability, and could achieve comparable or better assembly quality.

To resolve the large memory requirement problem of most OLC de novo assemblers, Paul et al. (2017) [50] employed string graph reduction algorithms, taking advantage of Spark. The proposed

Spark algorithms were evaluated against a very large sample dataset. The results showed that this dataset was assembled by the proposed Spark algorithms using 15 virtual machines in 0.5 hours, compared with the 7.5 hours achieved by the OLC-based Omega [51] assembler.

In addition, some new assembly algorithms have also been proposed, based on the Spark platform itself. In 2016, Pan et al. [52] put forward a new assembling algorithm, based on Spark, which employed the method of matching K-2 bit to simplify the de Bruijn graph. This algorithm was evaluated using six groups of DNA data in the NCBI database. Experimental results [52] showed that this strategy not only solved the problem of low efficiency based on the MapReduce algorithm, but also optimized the algorithm itself. The combination of these two aspects was very suitable for the large-scale assembly of DNA sequences. Moreover, the results also showed that the new Spark-based sequence-assembling algorithm ensured the accuracy of assembling results.

To address the problem of poor assembling precision and low efficiency, Dong et al. (2017) [53] proposed SA-BR-Spark, a new sequence assembly algorithm based on Spark. The authors first designed a precise assembling algorithm using the strategy of finding the source of reads based on the MapReduce and Eulerian path algorithm (SA-BR-MR). SA-BR-MR calculated 54 sequences, randomly selected from animal, plant and microorganism sequences in the NCBI database, with base lengths ranging from hundreds to tens of thousands. The matching rates of all 54 sequences were 100%. For each species, the algorithm also summarized the range of K that made the matching rates 100%. To verify the range of K values of hepatitis C virus and related variants, the K values of eight randomly selected hepatitis C virus variants were calculated. The results confirmed that the range of K of hepatitis C and related variants in NCBI were correct. After that, SA-BR-Spark was put forward. Experimental results [53] showed that SA-BR-Spark provided superior computational speed compared with SA-BR-MR.

SPARK IN SEQUENCE ANALYSIS

The GATK (Genome Analysis Toolkit) DNA analysis pipeline is widely used in genomic data analysis. Before Spark-based GATK tools were created, while several other tools had been developed to address the issue of scalability in the pipeline (such as Halvade [26] and Churchill [54]), they showed limited scalability, accuracy, and computational efficiency.

Therefore, in 2015, Mushtaq et al. [55] utilized Spark to propose a cluster-based GATK pipeline. To reduce the execution time, this approach kept data active in the memory between the map and reduce phases. By using active workload runtime statistics, it achieved a dynamic load-balancing

algorithm that could better utilize system performance. Experimental results [55] showed that this method achieved 4.5 times increased speed compared with the multi-threaded GATK pipeline on a single node. In addition, when executed on a four-node cluster, this approach was 63% faster than Halvade.

Then, in 2016, Deng et al. proposed HiGene [56], which employed Spark to enable multicore and multinode parallelization of the GATK pipeline. HiGene put forward a dynamic computing resource scheduler and an efficient data-skew mitigation method to improve performance. Experiments were conducted with the NA12878 whole human genome dataset. The results [56] showed that HiGene reduced the total running time from days to just under an hour. Furthermore, compared with Halvade, HiGene was also two times faster. Meanwhile, Li et al. employed Spark to propose GATK-Spark [57]. This paralleled the GATK pipeline by taking full account of compute, workload and I/O characteristics. It was built on top of the ADAM format [58]. Experimental results [57] showed that GATK-Spark decreased the total running time from 20 hours to 30 minutes on 256 CPU cores, which achieved more than 37-fold increased speeds.

Spark provides the opportunity for interactive NGS data processing. In 2014, Wiewiórka et al. proposed SparkSeq [59] to build and run genomic analysis pipelines in an interactive way by using Spark. Experimental results showed that SparkSeq achieved 8.4–9.15 times faster speeds than SeqPig. Moreover, it could accelerate data querying by up to 110 times, and reduce memory consumption by 13 times.

SPARK IN OTHER BIOLOGICAL APPLICATIONS

Spark in epigenetics

CpG islands are important markers that are essential in epigenetics [60]. However, investigation of CpG islands and their structures remains challenging. Before Spark-based applications were developed, while several methods had been proposed to determine the CPG islands (such as bisulfite modification-based methods), they were time-consuming and prohibitively expensive. Thus, Yu et al. [61] utilized Spark to propose a novel CpG box model and a Markov model to redefine and investigate the CpG island, which could greatly accelerate the analytic process. Experiments were conducted with human and mouse chromosome sequences; 24 chromosomes and 21 chromosomes. The results [61] showed that this cloud-assisted method had considerable accuracy and faster processing power (6–7 times faster with 10 cores) compared with sequential processing.

Spark in phylogeny

Phylogeny reconstruction is important in molecular evolutionary studies, but faces significant computational challenges. Before Spark-based tools were created, while several tools had been put forward for phylogeny reconstruction, they did not scale well, and there was a significant increase in the number of datasets. Therefore, in 2016, Xu et al. proposed CloudPhylo [62], a fast and scalable phylogeny reconstruction tool, making use of Spark. It evenly distributed the entire computational workload between working nodes. An experiment was conducted using 5220 bacteria whole genome DNA sequences. The results [62] showed that CloudPhylo took 24,508 seconds with one worker node, and it was able to scale well with increasing numbers of worker nodes. Moreover, CloudPhylo performed better than several existing tools when using more worker nodes. In addition, CloudPhylo achieved faster speeds on a larger dataset of about 100 Gb generated by simulation.

Spark in drug discovery

The identification of candidate molecules that affect disease-related proteins is crucial in drug discovery. Although the Chemogenomics project tries to identify candidate molecules using machine-learning predictor programs [63–65], these programs are slow, and cannot be easily extended to multiple nodes. To migrate existing programs to multinode clusters without changing the original programs, Harnie et al. proposed S-CHEMO [66], using Spark. In S-CHEMO, the intermediate data is immediately consumed again on the nodes that generated the data, reducing time and network bandwidth consumption. Experiments [66] compared S-CHEMO with the original pipeline, and showed almost linearly increased speeds on up to eight nodes. Moreover, this implementation also allowed easier monitoring.

Spark in single-cell RNA sequencing

Single-cell RNA sequencing (scRNA-seq) is crucial for understanding biological processes. Compared with standard bulk RNA-seq experiments, scRNA-seq experiments typically generate a greater number of cell profiles. Although several RNA-seq processing pipelines are already available (such as Halvade, SparkSeq and SparkBWA), they cannot process large numbers of profiles. Therefore, Falco [67] was created to process large-scale transcriptomic data in parallel by using Hadoop and Spark. Experiments were conducted with two public scRNA-seq datasets. The results [67] showed that, compared with a highly optimized single-node analysis, Falco was at least 2.6 times faster. Moreover, as the number of computing nodes increased, running time decreased.

Furthermore, it allowed users to employ the low-cost spot instances of AWS, which reduced the cost of analysis by 65%.

Spark in variant association and population genetics studies

Effectively analyzing thousands of individuals and millions of variants is a computationally intensive problem. Traditional parallel strategies, such as MPI/OpenMP show poor scalability. While Hadoop provides an efficient and scalable computing framework, it is heavily dependent on disk operations. Therefore, in 2015, O'Brien et al. proposed VariantSpark [68] to parallel population-scale tasks based on Spark and an associated machine-learning library, MLlib. Experiments were conducted on 3000 individuals with 80 million variants, and showed that VariantSpark was 80% faster than ADAM, the Hadoop/Mahout implementation, and ADMIXTURE [69]. Moreover, compared with R and Python implementations, it was more than 90 % faster. In 2017, Di et al. proposed SEQSpark [70] to perform rare variant association analysis using Spark. It was evaluated with whole-genome and simulated exome sequence data. The former was completed in 1.5 hours, and the latter in 1.75 hours. Moreover, it was always faster than Variant Association Tools and PLINK/SEQ, and in some cases running time was reduced to 1%.

Spark in other works

Biological simulations and experiments produce a large number of numerical datasets, and in 2017, Klein et al. proposed Biospark [71] to process these data. Biospark was based on Hadoop and Spark, comprising a set of Java, C++ and Python libraries. In addition, it provided the abstractions for parallel analysis of standard data types, including multidimensional arrays and images. To facilitate parallel analysis of some common datasets, it also provided APIs and file conversion tools, including Monte Carlo, molecular dynamics simulations, and time-lapse microscopy.

DISCUSSION

Spark is an in-memory iterative computing framework designed for large-scale data processing. It is suitable for applications that require iterative operations on specific datasets: the greater the amount of data, the higher the computational intensity, and the greater the benefits. When the data volume is small but the computational intensity is large, the benefit is relatively small. In addition, Spark is also suitable for applications where the amount of data is not particularly large, but real-time statistical analyses are required.

However, the nature of RDD means that Spark is not suitable for applications requiring asynchronous, fine-grained updates in execution, such as web service storage or incremental web crawlers and indexes. In addition, we must consider the potential complexity of creating and maintaining a Spark cluster. Moreover, when Spark runs on a commercial cloud-computing platform such as AWS, there is a certain delay in the transmission of large-scale datasets over the Internet. This issue does not exist when Spark runs on a local computer cluster. Furthermore, we need to learn a new API, and perhaps even a new language (especially given the functional programing nature of the API).

Although Spark has been applied in some areas of bioinformatics, and has achieved good results, its use in other areas—such as proteomics, biomedical texts, and metabolomics—has not yet been explored. Moreover, as cloud computing, and some web servers, become more and more available, some issues must be considered, such as the time cost of large amounts of input data from local to remote servers in slow networks, cloud computing fees, data security and privacy.

CONCLUSION

With the rapid development of NGS technology, a large number of genomic datasets have been generated, which poses a great challenge to traditional bioinformatics tools. For this reason, we have summarized relevant works about the use of Spark in bioinformatics, and have created a guideline on this topic. First, we make a comparison between Spark and Hadoop, and then outline the Spark cluster architecture, programming model, and processing mechanism. Then, we survey the use of Spark-based applications in NGS and other biological domains. Our survey means that researchers who wish to become involved in this field can now obtain a general understanding of the use of Spark in bioinformatics.

In summary, Spark is a fast and general-purpose computing framework designed for large-scale data processing. It ensures high fault tolerance and high scalability by introducing RDD abstraction and DAG scheduling. We believe that bioinformatics applications based on Spark will show promise in terms of performance for biological researchers in the future.

ABBREVIATIONS

API: application programming interface; AWS: Amazon Web Services; BWA: Burrows–Wheeler aligner; CPU: central processing unit; DAG: directed acyclic graph; GATK: Genome Analysis Toolkit; Gb: gigabase; GB: gigabyte; HDFS: Hadoop Distributed File System; MSA: multiple

sequence alignment; NCBI: National Center for Biotechnology Information; NGS: next-generation sequencing; OLC: overlap–layout–consensus; RDD: resilient distributed dataset; scRNA-seq: single-cell RNA sequencing; SIMD: Single Instruction Multiple Data; SW: Smith-Waterman algorithm.

ACKNOWLEDGEMENTS

The authors would like to thank the executive editor and the reviewers whose comments and constructive criticism helped in improving the quality of the manuscript. In addition, the authors thank Xiangke Liao and Kenli Li for their useful discussions and suggestions.

FUNDING

This work was supported by the National Key R&D Program of China (grant numbers 2017YFB0202600, 2016YFC1302500, 2016YFB0200400 and 2017YFB0202104); the National Natural Science Foundation of China (grant numbers 61772543, U1435222, 61625202, 61272056 and 61771331); and the Guangdong Provincial Department of Science and Technology (grant number 2016B090918122).

COMPETING INTERESTS

The authors declare that they have no competing interests.

AUTHOR CONTRIBUTIONS

RG and SP conceived the project and organized the work. All authors wrote parts of the manuscript and all authors have read and approved the final manuscript.

REFERENCES

1. Dean J, Ghemawat S: MapReduce: simplified data processing on large clusters. Communications of the ACM 2008, 51(1):107-113.
2. Zou Q, Li X-B, Jiang W-R et al: Survey of MapReduce frame operation in bioinformatics. Briefings in bioinformatics 2013, 15(4):637-647.
3. Zou Q, Hu Q, Guo M et al: HAlign: Fast multiple similar DNA/RNA sequence alignment based on the centre star strategy. Bioinformatics 2015, 31(15):2475-2481.

4. Gaggero M, Leo S, Manca S et al: Parallelizing bioinformatics applications with MapReduce. Cloud Computing and Its Applications 2008:22-23.

5. Leo S, Santoni F, Zanetti G: Biodoop: bioinformatics on hadoop. In: Parallel Processing Workshops, 2009 ICPPW'09 International Conference on: 2009. IEEE: 415-422.

6. Yang X-l, Liu Y-l, Yuan C-f et al: Parallelization of BLAST with MapReduce for long sequence alignment. In: Parallel Architectures, Algorithms and Programming (PAAP), 2011 Fourth International Symposium on: 2011. IEEE: 241-246.

7. Pireddu L, Leo S, Zanetti G: SEAL: a distributed short read mapping and duplicate removal tool. Bioinformatics 2011, 27(15):2159.

8. Schatz MC: CloudBurst: highly sensitive read mapping with MapReduce. Bioinformatics 2009, 25(11):1363-1369.

9. Nguyen T, Shi W, Ruden D: CloudAligner: A fast and full-featured MapReduce based tool for sequence mapping. BMC research notes 2011, 4(1):171.

10. Nordberg H, Bhatia K, Wang K et al: BioPig: a Hadoop-based analytic toolkit for large-scale sequence data. Bioinformatics 2013, 29(23):3014-3019.

11. Langmead B, Schatz MC, Lin J et al: Searching for SNPs with cloud computing. Genome biology 2009, 10(11):R134.

12. Kim D-k, Yoon J-h, Kong J-h et al: Cloud-scale SNP detection from RNA-Seq data. In: Data Mining and Intelligent Information Technology Applications (ICMiA), 2011 3rd International Conference on: 2011. IEEE: 321-323.

13. Hung C-L, Lin Y-L, Hua G-J et al: CloudTSS: a TagSNP selection approach on cloud computing. In: Grid and Distributed Computing. Springer; 2011: 525-534.

14. Zaharia M, Chowdhury M, Franklin MJ et al: Spark: Cluster computing with working sets. HotCloud 2010, 10(10-10):95.

15. Han Z, Zhang Y: Spark: A Big Data Processing Platform Based on Memory Computing. In: Seventh International Symposium on Parallel Architectures, Algorithms and Programming: 2016. 172-176.

16. Zaharia M, Chowdhury M, Das T et al: Resilient distributed datasets: A fault-tolerant abstraction for in-memory cluster computing. In: Proceedings of the 9th USENIX conference on Networked Systems Design and Implementation: 2012. USENIX Association: 2-2.

17. Convolbo MW, Chou J: Cost-aware DAG scheduling algorithms for minimizing execution cost on cloud resources. Journal of Supercomputing 2016, 72(3):985-1012.

18. Smith TF, Waterman MS: Identification of common molecular subsequences. Journal of Molecular Biology 1981, 147(1):195-197.

19. Zhao G, Ling C, Sun D: SparkSW: Scalable Distributed Computing System for Large-Scale Biological Sequence Alignment. In: Ieee/acm International Symposium on Cluster, Cloud and Grid Computing: 2015. 845-852.

20. Xu B, Li C, Zhuang H et al: DSA: Scalable Distributed Sequence Alignment System Using SIMD Instructions. In: Ieee/acm International Symposium on Cluster, Cloud and Grid Computing: 2017. 758-761.

21. Xu B, Li C, Zhuang H et al: Efficient Distributed Smith-Waterman Algorithm Based on Apache Spark. In: IEEE International Conference on Cloud Computing: 2017. 608-615.

22. Li H, Durbin R: Fast and accurate short read alignment with Burrows–Wheeler transform: Oxford University Press; 2009.

23. Li H, Durbin R: Fast and accurate long-read alignment with Burrows–Wheeler transform. Bioinformatics 2010, 26(5):589-595.

24. Li H: Aligning sequence reads, clone sequences and assembly contigs with BWA-MEM. 2013, 1303.

25. Abuín JM, Pichel JC, Pena TF et al: BigBWA: approaching the Burrows–Wheeler aligner to Big Data technologies. Bioinformatics 2015, 31(24):4003.

26. Decap D, Reumers J, Herzeel C et al: Halvade: scalable sequence analysis with MapReduce. Bioinformatics 2015, 31(15):2482-2488.

27. Al-Ars Z, Mushtaq H: Scalability Potential of BWA DNA Mapping Algorithm on Apache Spark. In: SIMBig: 2015. 85-88.

28. Abuín JM, Pichel JC, Pena TF et al: SparkBWA: Speeding Up the Alignment of High-Throughput DNA Sequencing Data. Plos One 2016, 11(5):e0155461.

29. Alars HMA: Streaming Distributed DNA Sequence Alignment Using Apache Spark. 2017.

30. Mirarab S, Nguyen N, Warnow T: PASTA: ultra-large multiple sequence alignment. In: International Conference on Research in Computational Molecular Biology: 2014. Springer: 177-191.

31. Liu K, Warnow TJ, Holder MT et al: SATe-II: very fast and accurate simultaneous estimation of multiple sequence alignments and phylogenetic trees. Systematic biology 2011, 61(1):90-106.

32. Abuín JM, Pena TF, Pichel JC: PASTASpark: multiple sequence alignment meets Big Data. Bioinformatics 2017, 33(18):2948-2950.

33. Miyazawa S: A reliable sequence alignment method based on probabilities of residue correspondences. Protein Engineering 1995, 8(10):999.

34. Katoh K, Standley DM: MAFFT Multiple Sequence Alignment Software Version 7: Improvements in Performance and Usability. Molecular Biology & Evolution 2013, 30(4):772-780.

35. Do CB, Mahabhashyam MS, Brudno M et al: ProbCons: Probabilistic consistency-based multiple sequence alignment. Genome Research 2005, 15(2):330.

36. Tommaso PD, Moretti S, Xenarios I et al: T-Coffee: a web server for the multiple sequence alignment of protein and RNA sequences using structural information and homology extension. Nucleic Acids Research 2011, 39(Web Server issue):13-17.

37. Lladós J, Guirado F, Cores F et al: PPCAS: Implementation of a Probabilistic Pairwise Model for Consistency-Based Multiple Alignment in Apache Spark; 2017.

38. Altschul S, Gish W, Miller W et al: Basic local alignment search tool. J. Mol. Biol. 1990.

39. C C, G C, V A et al: BLAST+: architecture and applications. Bmc Bioinformatics 2009, 10(1):421.

40. Darling AE, Carey L, Feng WC: The design, implementation, and evaluation of mpiBLAST. In.: Los Alamos National Laboratory; 2003.

41. Vouzis PD, Sahinidis NV: GPU-BLAST: using graphics processors to accelerate protein sequence alignment. Bioinformatics 2010, 27(2):182-188.

42. Matsunaga A, Tsugawa M, Fortes J: Cloudblast: Combining mapreduce and virtualization on distributed resources for bioinformatics applications. In: eScience, 2008 eScience'08 IEEE Fourth International Conference on: 2008. IEEE: 222-229.

43. Castro MRD, Tostes CDS, Dávila AMR et al: SparkBLAST: scalable BLAST processing using in-memory operations. Bmc Bioinformatics 2017, 18(1):318.

44. Zhou W, Li R, Yuan S et al: MetaSpark: a spark-based distributed processing tool to recruit metagenomic reads to reference genomes. Bioinformatics 2017, 33(7):1090-1092.

45. Niu B, Zhu Z, Fu L et al: FR-HIT, a very fast program to recruit metagenomic reads to homologous reference genomes. Bioinformatics 2011, 27(12):1704-1705.

46. Boisvert S, Laviolette F, Corbeil J: Ray: simultaneous assembly of reads from a mix of high-throughput sequencing technologies. Journal of Computational Biology A Journal of Computational Molecular Cell Biology 2010, 17(11):1519.

47. Simpson JT, Wong K, Jackman SD et al: ABySS: a parallel assembler for short read sequence data. Genome Research 2009, 19(6):1117.

48. Meng J, Wang B, Wei Y et al: SWAP-Assembler: scalable and efficient genome assembly towards thousands of cores. Bmc Bioinformatics 2014, 15(S9):S2.

49. Abu-Doleh A, Çatalyürek ÜV: Spaler: Spark and GraphX based de novo genome assembler. In: IEEE International Conference on Big Data: 2015. 1013-1018.

50. Paul AJ, Lawrence D, Ahn TH: Overlap Graph Reduction for Genome Assembly using Apache Spark. In: The ACM International Conference: 2017. 613-613.

51. Haider B, Ahn TH, Bushnell B et al: Omega: an Overlap-graph de novo Assembler for Metagenomics. Bioinformatics 2014, 30(19):2717-2722.

52. Pan X, Fu X-L, Dong G-F et al: DNA sequence splicing algorithm based on Spark. In: Industrial Informatics-Computing Technology, Intelligent Technology, Industrial Information Integration (ICIICII), 2016 International Conference on: 2016. IEEE: 52-56.

53. Dong G, Fu X, Li H et al: An Accurate Sequence Assembly Algorithm for Livestock, Plants and Microorganism Based on Spark. International Journal of Pattern Recognition & Artificial Intelligence 2017, 31(8).

54. Kelly BJ, Fitch JR, Hu Y et al: Churchill: an ultra-fast, deterministic, highly scalable and balanced parallelization strategy for the discovery of human genetic variation in clinical and population-scale genomics. Genome biology 2015, 16(1):6.

55. Mushtaq H, Al-Ars Z: Cluster-based Apache Spark implementation of the GATK DNA analysis pipeline. In: Bioinformatics and Biomedicine (BIBM), 2015 IEEE International Conference on: 2015. IEEE: 1471-1477.

56. Deng L, Huang G, Zhuang Y et al: HiGene: A high-performance platform for genomic data analysis. In: IEEE International Conference on Bioinformatics and Biomedicine: 2016. 576-583.

57. Li X, Tan G, Zhang C et al: Accelerating large-scale genomic analysis with Spark. In: Bioinformatics and Biomedicine (BIBM), 2016 IEEE International Conference on: 2016. IEEE: 747-751.

58. Massie M, Nothaft F, Hartl C et al: Adam: Genomics formats and processing patterns for cloud scale computing. EECS Department, University of California, Berkeley, Tech Rep UCB/EECS-2013-207 2013.

59. Wiewiórka MS, Messina A, Pacholewska A et al: SparkSeq: fast, scalable and cloud-ready tool for the interactive genomic data analysis with nucleotide precision. Bioinformatics 2014, 30(18):2652-2653.

60. Erkek S, Hisano M, Liang C-Y et al: Molecular determinants of nucleosome retention at CpG-rich sequences in mouse spermatozoa. Nature structural & molecular biology 2013, 20(7):868-875.

61. Yu N, Li B, Pan Y: A cloud-assisted application over apache spark for investigating epigenetic markers on DNA genome sequences. In: Big Data and Cloud Computing (BDCloud), Social Computing and Networking (SocialCom), Sustainable Computing and Communications (SustainCom)(BDCloud-SocialCom-SustainCom), 2016 IEEE International Conferences on: 2016. IEEE: 67-74.

62. Xu X, Ji Z, Zhang Z: CloudPhylo: a fast and scalable tool for phylogeny reconstruction. Bioinformatics 2016, 33(3):438-440.

63. Wale N: Machine learning in drug discovery and development. Drug Development Research 2011, 72(1):112-119.

64. Costello JC, Heiser LM, Georgii E et al: A community effort to assess and improve drug sensitivity prediction algorithms. Nature biotechnology 2014, 32(12):1202-1212.

65. Sastry GM, Inakollu VS, Sherman W: Boosting virtual screening enrichments with data fusion: coalescing hits from two-dimensional fingerprints, shape, and docking. Journal of chemical information and modeling 2013, 53(7):1531-1542.

66. Harnie D, Saey M, Vapirev AE et al: Scaling machine learning for target prediction in drug discovery using apache spark. Future Generation Computer Systems 2017, 67:409-417.

67. Yang A, Troup M, Lin P et al: Falco: a quick and flexible single-cell RNA-seq processing framework on the cloud. Bioinformatics 2016, 33(5):767-769.

68. O’Brien AR, Saunders NFW, Guo Y et al: VariantSpark: population scale clustering of genotype information. Bmc Genomics 2015, 16(1):1-9.

69. Alexander DH, Novembre J, Lange K: Fast model-based estimation of ancestry in unrelated individuals. Genome Research 2009, 19(9):1655.

70. Di Z, Zhao L, Li B et al: SEQSpark: A Complete Analysis Tool for Large-Scale Rare Variant Association Studies Using Whole-Genome and Exome Sequence Data. American Journal of Human Genetics 2017, 101(1):115.

71. Klein M, Sharma R, Bohrer CH et al: Biospark: scalable analysis of large numerical datasets from biological simulations and experiments using Hadoop and Spark. Bioinformatics 2017, 33(2):303-305.

Table 1 Bioinformatics tools and algorithms based on Apache Spark

**Name**	**Function**	**Features**	**Pros/Cons**	**Reference**
SparkSW	Alignment and mapping	Consists of three phases: data preprocessing, SW as map tasks, and top K records as reduce tasks	Load-balancing, scalable, but without the mapping location and traceback of optimal alignment	[19]
DSA	Alignment and mapping	Leverages data parallel strategy based on SIMD instruction	Up to 201 times increased speed over SparkSW and almost linearly increased speed with increasing numbers of cluster nodes	[20]
CloudSW	Alignment and mapping	Leverages SIMD instruction, and provides API services in the cloud	Up to 3.29 times increased speed over DSA and 621 times increased speed over SparkSW; high scalability and efficiency	[21]
SparkBWA	Alignment and mapping	Consists of three main stages: RDD creation, map, and reduce phases; employs two independent software layers	For shorter reads, averages 1.9x and 1.4x faster than SEAL and pBWA. For longer reads, averages 1.4x faster than BigBWA and Halvade, but requires the data availability in HDFS	[28]
StreamBWA	Alignment and mapping	Input data are streamed into the cluster directly from a compressed file	~2x faster than non-streaming approach, and 5x faster than SparkBWA	[29]
PASTASpark	Alignment and mapping	Employs an in-memory RDD of key-value pairs to parallel the calculating MSA phase	Up to 10x faster than single-threaded PASTA; ensures scalability and fault tolerance	[32]
PPCAS	Alignment and mapping	Based on the MapReduce processing paradigm in Spark	Better with a single node and shows almost linearly increased speeds with increasing numbers of nodes	[37]
SparkBLAST	Alignment and mapping	Utilizes *pipe* operator and Spark RDDs to call BLAST as an external library	Outperforms CloudBLAST in terms of speed, scalability and efficiency	[43]
MetaSpark	Alignment and mapping	Consists of five steps: constructing k-mer RefindexRDD, constructing k-mer ReadlistRDD, seeding, filtering, and banded alignment	Recruits significantly more reads than SOAP2, BWA and LAST, and more reads by ~4 than FR-HIT; shows good scalability and overall high performance	[44]
Spaler	Assembly	Employs Spark’s GraphX API; consists of two main parts: de Bruijn graph construction, and contig generation	Shows better scalability and achieves comparable or better assembly quality than ABySS, Ray, and SWAP-Assembler	[49]
SA-BR-Spark	Assembly	Under the strategy of finding the source of reads; based on the Spark platform	Shows a superior computational speed than SA-BR-MR	[53]
HiGene	Sequence analysis	Puts forward a dynamic computing resource	Reduces total running time from days to just under	[56]

		scheduler, and an efficient way of mitigating data skew	nearly an hour; 2x faster than Halvade	
GATK-Spark	Sequence analysis	Takes full account of compute, workload, and I/O characteristics	Achieves more than 37 times increased speed	[57]
SparkSeq	Sequence analysis	Builds and runs genomic analysis pipelines in an interactive way by using Spark	Achieves 8.4–9.15 times faster speeds than SeqPig; accelerates data querying up to 110 times and reduces memory consumption by 13 times	[59]
CloudPhylo	Phylogeny	Evenly distributes entire workloads between worker nodes	Shows good scalability and high efficiency; the Spark version is better than the Hadoop version	[62]
S-CHEMO	Drug discovery	Intermediate data are immediately consumed again on the producing nodes, saving time and bandwidth	Shows almost linearly increased speeds on up to 8 nodes compared with the original pipeline	[66]
Falco	Single-cell RNA sequencing	Consist of a splitting step, an optional preprocessing step, and the main analysis step	At least 2.6x faster than a highly optimized single-node analysis; running time decreases with increasing numbers of nodes	[67]
VariantSpark	Variant association and population genetics studies	Parallels population-scale tasks based on Spark and the associated MLlib	80% faster than ADAM (Hadoop/Mahout version), and ADMIXTURE; more than 90% faster than R and Python implementations	[68]
SEQSpark	Variant association and population genetics studies	Splits large-scale datasets into many small blocks to perform rare variant association analyses	Always faster than Variant Association Tools and PLINK/SEQ, and in some cases, running time is reduced to 1%	[70]
BioSpark	Data-parallel analysis on large, numerical datasets	Consists of a set of Java, C++ and Python libraries, abstractions for parallel analysis of standard data types, some APIs and file conversion tools	Convenient, scalable, and useful; has domain-specific features for biological applications	[71]

Figure 1 Click here to access/download;Figure;Figure 1.docx

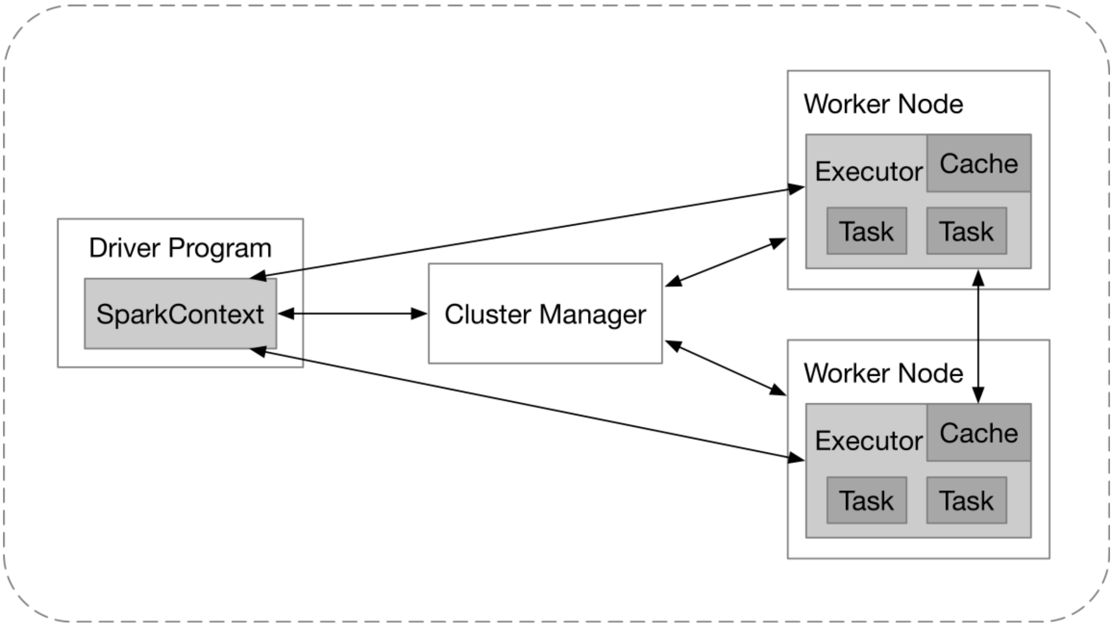

Figure 1: The cluster architecture of Spark.

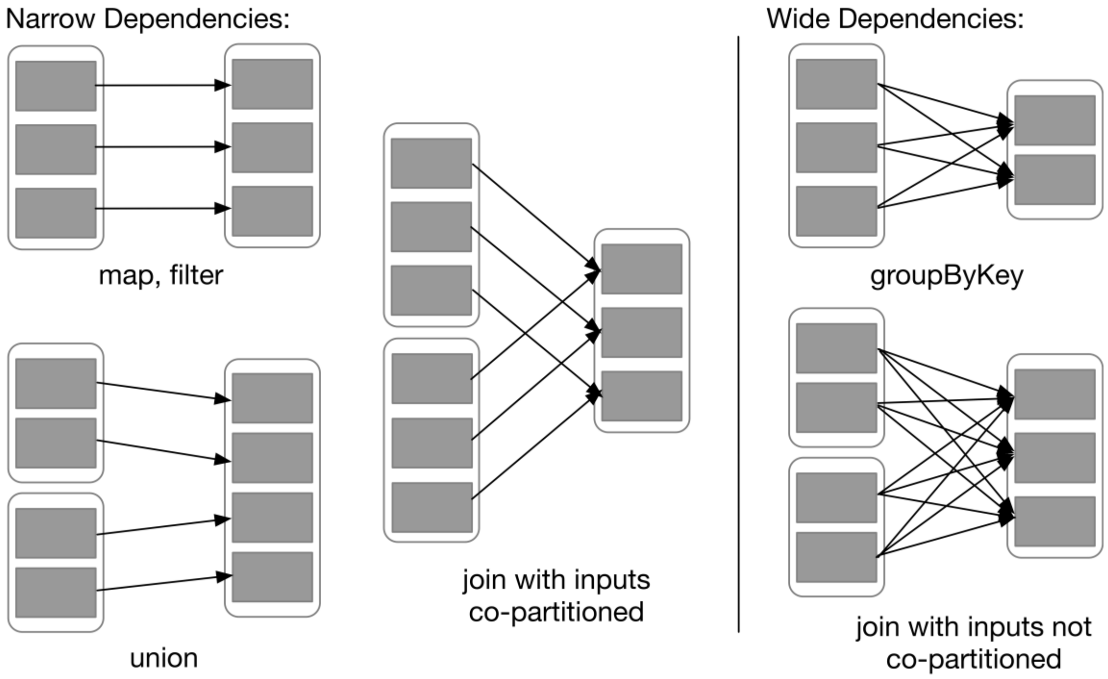

Figure 2: Some examples of narrow and wide dependencies. Each box is an RDD, where the partition is shown as a shaded rectangle.

 Click here to access/download;Figure;Figure 3.docx

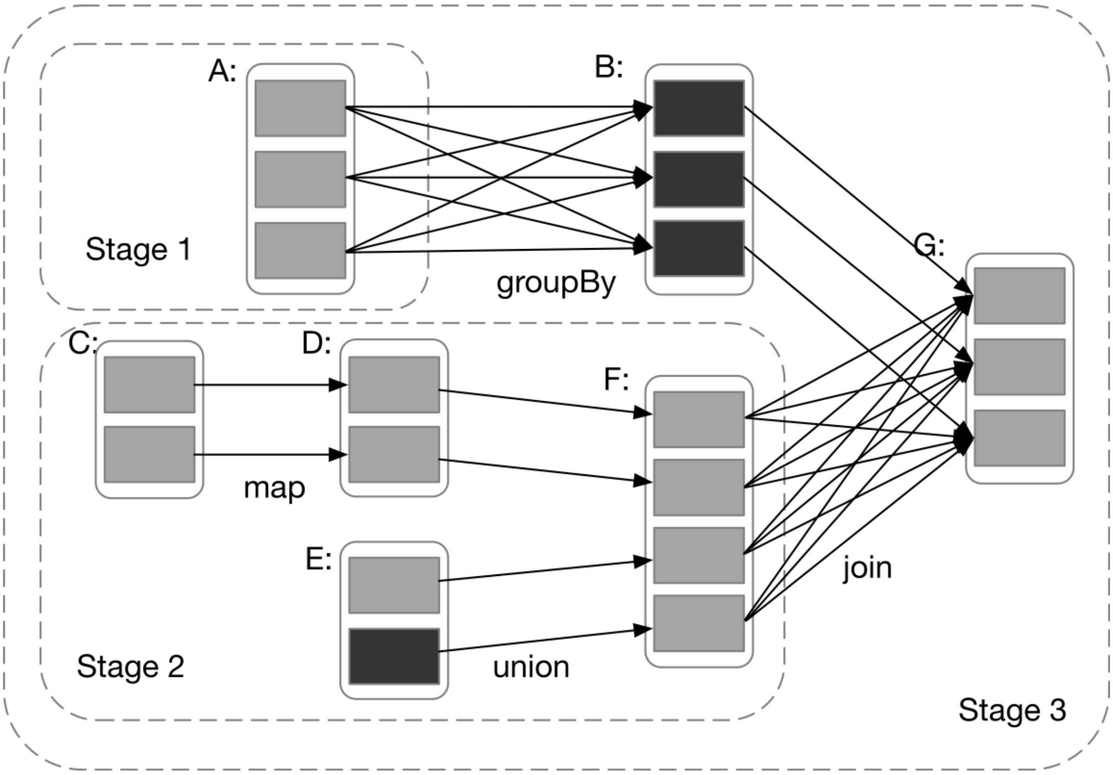

Figure 3: An example of how Spark computes job stages. Boxes with solid outlines are RDDs. Partitions are shaded rectangles and are black if they are already in memory. To run an action on RDD G, we build stages at wide dependencies and pipeline narrow transformation inside each stage. In this case, the output RDD of stage 1 is already in memory, so we run stage 2, and then stage 3.

Click here to access/download
Supplementary Material
Cover Letter.docx
