# Supplementary material for: Bioinformatics applications on Apache Spark
Source: Gigascience. 2018 Aug 7;7(8):giy098. doi: 10.1093/gigascience/giy098 (PMC6113509; doi:10.1093/gigascience/giy098)
Supplement: Supplemental Files [file giy098_supplemental_files.docx]

Dear Editors:

We would like to submit the enclosed manuscript entitled “Bioinformatics Application on Apache Spark”, which we wish to be considered for publication in “GigaScience”. No conflict of interest exits in the submission of this manuscript, and manuscript is approved by all authors for publication. I would like to declare on behalf of my co-authors that the work described was original research that has not been published previously, and not under consideration for publication elsewhere, in whole or in part. All the authors listed have approved the manuscript that is enclosed.

First, the Apache Spark gives researchers a possibility of supporting both in-memory and on-disk computations in a fault tolerant manner, by using distributed memory abstractions known as Resilient Distributed Datasets (RDDs).

Second, the Apache Spark supports various system workloads such as batch processing, iterative, interactive and stream computing, which can be 100× faster in memory access and 10x faster on disk access than Apache Hadoop.

Third, our previous job is about Hadoop in bioinformatics. Related paper entitled “Survey of MapReduce frame operation in bioinformatics” has been published in “Briefings in Bioinformatics” and cited 151 times. We believe this manuscript will attract readers as well.

We deeply appreciate your consideration of our manuscript, and we look forward to receiving comments from the reviewers. If you have any queries, please don’t hesitate to contact me at the address below.

Thank you and best regards!

Yours sincerely,

Runxin Guo

[runxinguo@yahoo.com](mailto:runxinguo@yahoo.com)

Corresponding author:

Shaoliang Peng

[pengshaoliang@nudt.edu.cn](mailto:pengshaoliang@nudt.edu.cn)

Xiaodong Fang

[fangxd@bgitechsolutions.com](mailto:fangxd@bgitechsolutions.com)

Quan Zou

[zouquan@nclab.net](mailto:zouquan@nclab.net)
