# Supplementary material for: Bioinformatics applications on Apache Spark
Source: Gigascience. 2018 Aug 7;7(8):giy098. doi: 10.1093/gigascience/giy098 (PMC6113509; doi:10.1093/gigascience/giy098)
Supplement: Response_to_Reviewer_Comments_Original_Submission.pdf [file giy098_response_to_reviewer_comments_original_submission.pdf]

Author's Response To Reviewer Comments

Close

Dear Editor and Reviewers:
Thank you for your letter and for the reviewers' comments concerning our manuscript entitled "Bioinformatics Application on Apache Spark" (GIGAD1800131). Those comments are all valuable and very helpful for revising and improving our paper, as well as the important guiding significance to our research. We have studied the comments carefully and have made correction which we hope meet with approval. Revised portion are marked in red in the paper. The main corrections in the paper and the responds to the editor and reviewers' comments are as following:
To Editor:
Comment 1: While one of the reviewers doesn't like the figures, another thinks you need more, and generally we side with the "more figures in a review the better". In particular, we would encourage improvements and more infographic like detail if you can. Improvements to the language and writing is also required, although if it passes review we will send it to a copy editor.
Response: Appreciate for your comment, taking into account the reviewers' comments and your opinions, we have improved the figures, removed unnecessary figures, and added a few necessary figures to help readers better understand the Spark framework and operating mechanism. In addition, we have improved my language and writing.
To Reviewer 1:
Comment 1: The paper would benefit from a *slightly* deeper description of the Spark architecture, in particular explaining the nature of DAGs and the way in which they permit optimizations. Also, some mention of the two deploy modes (where the driver program can either be run on the client machine, or on a worker node).
Response: Appreciate for your comment, in "THE SPARK FRAMEWORK" section, we have made a more detailed description of the Spark architecture, explained the nature of DAGs and the way in which they permit optimizations, introduced the two deploy modes: cluster mode and client mode.
Comment 2: The paper would also benefit from a section that examines the potential downsides of using Spark, for example the potential complexity in creating and maintaining a Spark cluster, and the learning curve involved in learning a new API and perhaps even language (especially given the Functional Programming nature of the API).
Response: Appreciate for your comment, in "DISCUSSION" section, we have discussed the disadvantages of Spark, including the applications that Spark is not suitable for, the complexity of creating and maintaining a Spark cluster, the time cost of large-scale input data from local to remote servers in slow networks, the complex learning curve.
Comment 3: With regards to style, there are a number of places in the paper where the definite article is used where it shouldn't, and vice versa. In the interest of readability and not distracting the reader, these should be addressed. A similar point can be made with regard to the overuse of certain prepositions (e.g. "besides"), which are called out in detail in the next section.
Response: Appreciate for your comment, we have addressed the use of the definite article in paper, and replaced "besides" with "in addition", "moreover", and "furthermore".
Comment 4: p.1 line 28: "data" is treated as a plural in the rest of the paper, therefore "pose" rather than "poses".
Response: Appreciate for your comment, we have changed "poses" to "pose".
Comment 5: p.1 line 34: "by introducing resilient distributed dataset" should be "by

introducing the resilient distributed dataset" (i.e. use of definite article)
Response: Appreciate for your comment, we have changed “by introducing resilient distributed dataset” to “by introducing the resilient distributed dataset”.
Comment 6: p.1 line 40: In the end, we discussed the challenges...and the future work...". I haven't found this discussion in the paper.
Response: Appreciate for your comment, we have deleted this sentence from “ABSTACT” section, but added a “DISCUSSION” section to discuss the advantages and disadvantages of Spark, some issues to be considered about cloud computing in the future and other bioinformatics fields that have not yet been involved.
Comment 7: p.2 line 4: "MapReduce preforms" should be "MapReduce performs".
Response: Appreciate for your comment, we have changed “preforms” to “performs”.
Comment 8: p.2 line 21: "introducing resilient distributed dataset" should "introducing the resilient distributed dataset".
Response: Appreciate for your comment, we have changed “introducing resilient distributed dataset” to “introducing the RDD abstraction”.
Comment 9: p.2 line 38: The documentation of Spark describes the driver program as "The process running the main () function of the application and creating the SparkContext". It does not "deploy the Spark operating environment". Perhaps the authors meant "deploy TO the Spark operating environment" but even here this would be incorrect, as the sparksubmit script does this.
Response: Appreciate for your comment, we have rewritten this part to provide the correct description in “THE SPARK FRAMEWORK” section.
Comment 10: p.2, line 43: As well as Scala, Spark provides APIs in Java, Python and more recently R. This flexibility is important to researchers when deciding whether to use Spark or not.
Response: Appreciate for your comment, we have mentioned that Spark provides advanced APIs in Java, Scala, Python and R in “THE SPARK FRAMEWORK” section.
Comment 11: p.2, line 58: It is questionable that "the most important feature of RDD" is the fault tolerance. Certainly, it is "an important feature".
Response: Appreciate for your comment, we have changed this part to “RDD achieves fault tolerance through a notion of lineage…” in “THE SPARK FRAMEWORK” section.
Comment 12: p.3, line 13: The referenced image appears to be an _example_ of a spark task flow chart, rather than _the general_ Spark task processing flow. For the reader's sake, the paper should either describe what this particular task is doing (including the fact that it is reading and writing to HDFS in this case). Otherwise the reader may form incorrect opinions or simply be confused. Alternatively, drop the figure entirely.
Response: Appreciate for your comment, we have updated this figure to show an example of how Spark computes job stages in “THE SPARK FRAMEWORK” section.
Comment 13: p.3, line 17: "Besides" as preposition. This is a little colloquial and has an additional "in any case" meaning. To avoid distracting the reader, consider replacing "besides" as a preposition with alternatives like "In addition", "Moreover", "Furthermore". This can be applied to the rest of the paper, and I won't call any more out by line number.
Response: Appreciate for your comment, we have replaced “besides” with “in addition”, “moreover”, and “furthermore” in paper.
Comment 14: p.4, line 4: "BurrowWheeler aligner" either "The BurrowWheeler aligner" or "BurrowWheeler alignment" read better
Response: Appreciate for your comment, we have changed "BurrowWheeler aligner" to “The BurrowWheeler aligner”.
Comment 15: p.4, line 19: "Results showed" "The results showed"
Response: Appreciate for your comment, we have changed “Result showed” to “The results

showed”.
Comment 16: p.4, line 32: "achieved the average speedup of" "achieved an average speedup of"
Response: Appreciate for your comment, we have changed “achieved the average speedup of” to “achieved an average speedup of”.
Comment 17: p.6, line 58: Drop "And" from the start of the sentence.
Response: We have dropped “And” from the start of the sentence.
Comment 18: p.7, line 1: "Experiments results" "Experimental results”
Response: Appreciate for your comment, we have changed “Experiments results” to “Experimental results”.
Comment 19: p.7, line 4: Perhaps it's worth pointing out that this is an example of the platform itself suggesting a new algorithm, rather than simply re-implementing an existing algorithm on the new platform. Similarly, for line 19 of this page.
Response: Appreciate for your comment, we have pointed out that these two are examples of the Spark platform itself suggesting new algorithms in “SPARK IN ASSEMBLY” section.
Comment 20: p.7, line 23: Is SABRMR running on Hadoop? (I ask because MR is a valid algorithm on Spark as well).
Response: Appreciate for your comment, SA-BR-MR is running on Hadoop according to the reference paper.
Comment 21: p.8, line 17: "Results..." "The results..."
Response: Appreciate for your comment, we have changed “Results” to “The results”.
Comment 22: p.8, line 41: "noises" "noise".
Response: Appreciate for your comment, we have changed “noises” to “noise”.
Comment 23: p.9, lines 2339: The epigenetics example just calls out the advantage of parallelization compared to sequential processing. Was there a parallelized attempt, perhaps using Hadoop, that the Yu N et al paper could demonstrate a superiority to?
Response: Appreciate for your comment, we have reviewed lots of related papers, but did not find some parallelized attempts.
Comment 24: p.10, line 8: "Saprk" "Spark"
Response: Appreciate for your comment, we have changed “Saprk” to “Spark”.
Comment 25: p.10, line 15: The term "checkpointing" is not explained even in the body of the referenced paper (Harnie D et al) and is probably best dropped.
Response: Appreciate for your comment, we have dropped the term “checkpointing” from the sentence.
Comment 26: p.11, line 43: Key Points section: I would respectfully disagree with the following statement: "We introduce the Apache Spark framework in detail, helping researchers to understand its architecture, programming model and processing mechanism." I think the authors do a good job of firstly, giving an *overview* of Spark (notwithstanding earlier points about getting into more detail), but I don't think this paper is a *detailed* description of Spark, its architecture or its programming model. Indeed, I don't think it *needs* to be the survey of *how Spark has successfully been used* is probably of primary interest to most readers. But it's best to be clear about the scope of the paper in the Key Points so as to set readers' expectations correctly.
Response: Appreciate for your comment, we have updated this key point to point out that we outline the Apache Spark framework to researchers to understand its architecture, programming model and processing mechanism, and we have made a more detailed description of the Spark architecture in “THE SPARK FRAMEWORK” section.
Comment 27: p.11, line 48: Key Points section: Similarly, to above, I would edit the third Key Point to set readers' expectations correctly. The paper in its current form does not

include a "discussion on the future of parallel computing in bioinformatics" (and in my opinion it does not need to).
Response: Appreciate for your comment, considering your opinions and that of other reviewers, we have added a “DISCUSSION” section to discuss Spark’s strengths, weaknesses, and challenges faced in this field.
To Reviewer 2:
Comment 1: While I agree, Spark has a lot of advantages over other parallel and distributed computing frameworks such as MapReduce, I feel the current tone and content are too one-sided. In my own experience, Spark is mostly only useful for processing very large amount of data. For smaller data sets, the scalability gained by Spark may not be enough to justify the upfront time required for setting up and configuring a Spark-enabled system. Also, there is no discussion on computing hardware requirement (local computer cluster or commercial cloud computing platforms), and issues related to transfer of large data sets over the Internet. All these issues need to be discussed.
Response: Appreciate for your comment, we have discussed the strengths and weaknesses of Spark and issues related to transfer of large data sets over the Internet on local computer cluster or commercial cloud computing in “DISCUSSION” section, and pointed out the official proposal for hardware requirements in “THE SPARK FRAMEWORK” section.
Comment 2: The sections on 'Spark in motif analysis' and 'Spark in genomic inference' are poorly written. The terms 'motif' and 'genomic inference' are not properly defined. Do they mean transcription factor binding motifs, or simply frequently occurring DNA sequence some defined regions in the genome (e.g., promoters, enhancers, etc.)? Also, the term 'genomic data inference' is not well defined. Presumably the authors are referring to inference in a population genomics context.
Response: Appreciate for your comment, here, motif refers to transcription factor binding sites (TFBS), genomic inference refers to the inference in population genomics text. We have updated these two sections in the paper to provide correct descriptions about the terms ‘motif’ and ‘genomic inference’.
Comment 3: The caption of all four figures are way too simple. In most cases, especially for complicated flow diagrams like Fig 1 and Fig 3, there is no explanation or description of the content of the figure. I believe the authors intends to illustrate the inner working of Spark using these figures. Nonetheless, the content of these figures is not explained in the caption nor the main text.
Response: Appreciate for your comment, considering your opinions and that of other reviewers, we have updated the figures, removed unnecessary figures, and added a few necessary figures to help readers better understand the Spark framework and operating mechanism. Moreover, we have added some explanations and descriptions of the content of the figures in the captions.
Comment 4: Despite the authors claiming they 'discuss the future of parallel computing in bioinformatics' (Key Points #3), the manuscript barely talks about the future, other than saying 'Spark will provide promising performance for biological researchers in the future' (Conclusions). I think this is a lost opportunity. What do the authors see as the major limitations in the field at the moment? New hardware? Better integration with cloud computing platforms? New application areas, such as proteomics, metabolomics, biomedical text, electronic medical health record, etc.?
Response: Appreciate for your comment, we have updated the Key Points #3 and discussed some issues to be considered about cloud computing in the future and pointed out other bioinformatics fields that have not yet been involved.
Comment 5: In multiple occasions, the authors use 'And' to begin a sentence. I do not think it is grammatically correct.

Response: Appreciate for your comment, we have dropped “And” from all related sentences in paper.
Comment 6: Throughout the manuscript, author names are often cited as ([last name] [first initials]), but sometimes they are cited as ([last name] [all initials]) or ([last name] [first name]). Please make sure names are formatted consistently.
Response: Appreciate for your comment, we have updated author names as ([last name] [first initial]) in paper to make sure names are formatted consistently.
To Reviewer 3:
Comment 1: The manuscript needs to be rewritten to provide more practical information to bioinformatics research users how Apache Spark based bioinformatics tools are actively used in specific bioinformatics research domains and what are the advantages of using the Apache Spark.
Response: Appreciate for your comment, we have rewritten the paper to provide more practical information to bioinformatics research users how Apache Spark based bioinformatics tools are actively used in specific bioinformatics research domains and what are the advantages of using the Apache Spark. In “THE SPARK FRAMEWORK” section, we have made a more detailed description of the Spark architecture and the main abstraction RDD, explained the nature of DAGs and the way in which they permit optimizations, provided the official proposal for hardware requirements to help researchers better understand and use Spark. Moreover, we have added the “DISCUSSION” section to discuss the strengths and weaknesses of Spark, the applications that Spark is suitable for, and some issues must be considered. Researchers can comprehensively consider how to use Spark through these contents combined with biological issues in their field.
Comment 2: Table 1 shows basic APIs of Apache Spark. The reviewer cannot get a point why the authors included this table. Delete the table or keep the table with presenting how the APIs could be used in the bioinformatics tools.
Response: Appreciate for your comment, we have removed the Table 1 from the paper.
Comment 3: In the first paragraph of the Introduction section, "However, the existing bioinformatics tools cannot effectively handle such a large amount of data. In order to solve the issues, MapReduce, a programming model for parallel computation of large datasets, has been proposed [1]." sentences should be updated. MapReduce has not been proposed for bioinformatics tool. MapReduce framework was proposed for general purpose big data analysis in the distributed manner.
Response: Appreciate for your comment, we have updated these sentences to point out that MapReduce was proposed for processing large-scale datasets in a distributed manner in information technology rather than for bioinformatics tools.
Comment 4: The manuscript did not mention about "DataFrames" API that is an extension of RDD. Most of new Spark features use this new DataFrames, and it should be addressed in the manuscript.
Response: Appreciate for your comment, we have mentioned the two extensions of RDD: DataFrame and Dataset in “The SPARK FRAMEWORK” section. Users can seamlessly switch between the three through simple API calls.
Comment 5: Table 2 only shows the application domain, program name, URL, references. The table should be updated to include more meaningful informatics of tools such as pros/cons or specific features or the tools.
Response: Appreciate for your comment, we have updated the Table 2 to provide more meaningful informatics, including name, function, features, pros/cons and reference of applications.
Comment 6: Figures 1 to 4 are not necessary in the manuscript. Instead of these figures, the authors should consider how to express the relationship of bioinformatics tools and Apache

Spark effectively using figures.
Response: Appreciate for your comment, considering the opinions of editor and other reviewers, we have improved the figures, removed unnecessary figures, and added a few necessary figures to help readers better understand the Spark framework and operating mechanism. Figure 1 is mainly used to introduce Spark's cluster architecture, explain its main components and functions, and how the Spark application runs on the cluster. Figure 2 is mainly used to show some examples of narrow and wide dependencies to help readers understand RDD's dependencies. Because these dependencies are important in the splitting job stages of Spark, so we add this figure. Figure 3 is mainly used to show an example of how Spark computes job stages to help readers understand the RDD operating mechanism and DAG scheduling. So, we add this figure. Moreover, we carefully considered your comments about how to express the relationship of bioinformatics tools and Apache Spark effectively using figures. We feel that the relationship between bioinformatics tools and Spark cannot be well expressed in figures. Because the variated items in bioinformatics can cover almost all sorts of computational optimization problems. The Spark framework represents a possible solution for tasks in parallel computing with some certain characteristics. So, we tried our best to provide readers with some practical advices on using Spark framework based on computational characteristics of problems in "Discussion" section. In "DISCUSSION" section, we have discussed the strengths and weaknesses of Spark, the applications that Spark is suitable for, and some issues must be considered to help researchers to consider how to use Spark combined with biological issues in their field.
We tried our best to improve the manuscript and made some changes in the manuscript. These changes will not influence the content and framework of the paper. And here we did not list the changes but marked in revise paper. We appreciate for Editor/Reviewers' warm work earnestly, and hope that the correction will meet with approval. Once again, thank you very much for your comments and suggestions.
Yours
Sincerely
Runxin GUO, Yi ZHAO, Xiangke LIAO, Kenli LI, Quan ZOU, Xiaodong FANG, Shaoliang PENG

Close
