# Supplementary material for: Bioinformatics applications on Apache Spark
Source: Gigascience. 2018 Aug 7;7(8):giy098. doi: 10.1093/gigascience/giy098 (PMC6113509; doi:10.1093/gigascience/giy098)
Supplement: Response_to_Reviewer_Comments_Revision_1.pdf [file giy098_response_to_reviewer_comments_revision_1.pdf]

Author's Response To Reviewer Comments

Close

Dear Editor and Reviewers:
Thank you for your letter and for the reviewers' comments concerning our manuscript entitled "Bioinformatics Application on Apache Spark" (GIGAD1800131R1). Those comments are all valuable and very helpful for revising and improving our paper, as well as the important guiding significance to our research. We have studied the comments carefully and have made correction which we hope meet with approval. Revised portion are marked in red in the paper. The main corrections in the paper and the responds to the editor and reviewers' comments are as following:
To Editor:
Comment 1: Please also be careful about recycling text from other sources. For example, on p5 there is quite a lot of text recycled from the following paper:
https://ieeexplore.ieee.org/document/8251287/
Response: Appreciate for your comment, considering the recycling text from other sources, we have updated the paper to meet the criteria and checked the paper through our own database.
To Reviewer 2:
Comment 1: Nonetheless, I still feel the sections on 'Spark in motif analysis' and 'Spark in population genomic inference' are still very confusing and potentially misleading. For example, calling 'motif' a type of noise in NGS data, and naively equating motifs with TFBS is incorrect. This indicates the authors may not fully understand these terms. Also, I still do not understand what do they mean by 'genomic inference' estimating ancestry? population admixture? Simply reiterating words in my questions is not sufficient. The authors should either significantly rewrite both sections and have these checked by experts in these areas, or should simply remove them. I am happy with the rest of the manuscript.
Response: Appreciate for your comment, considering the opinions of experts in these areas, we have removed the sections on 'Spark in motif analysis' and 'Spark in population genomic inference'. In the reference paper, the motif is also called transcription factor binding sites (TFBS). We agree with you and think it is incorrect which will mislead the readers, so we have decided to remove the section on 'Spark in motif analysis'. In the reference paper, the genomic inference is not clearly defined. Moreover, we have reviewed lots of related papers and did not find authoritative literature to define genomic inference. In order to avoid misleading and confusing readers, we have decided to remove the section on 'Spark in population genomic inference'.
To Reviewer 3:
Comment 1: To separate different applications and approaches, authors used paragraph break (single line space). However, several paragraphs are excessively short. For example, page 2 line 53 and page 7 line 49. Please update the manuscript to have better structure of paragraphs.
Response: Appreciate for your comment, taking into account the opinions of other reviewers and editor, we have updated the manuscript as much as possible based on the content of the manuscript to have better structure of paragraphs.
Comment 2: Table 1 is mentioned at the last paragraph of Discussion section. Please cite the table in the beginning of manuscript (just after the Introduction section) for users to easily catch the features of many Sparkbased bioinformatics applications.
Response: Appreciate for your comment, we have cited the Table 1 in the beginning of manuscript (just after the Introduction section) for users to easily catch the features of many

Sparkbased bioinformatics applications.
Comment 3: Page 2: Line 49: As in Figure 1 > As shown in Figure 1
Response: Appreciate for your comment, we have changed "As in Figure 1" to "As shown in Figure 1"
Comment 4: Page 2: Line 49: Spark application runs as independent processes on the cluster and are coordinated by the SparkContext in the driver program > Each Spark application runs as independent process on the cluster coordinated by the SparkContext in the driver program
Response: Appreciate for your comment, we have changed "Spark application runs as independent processes on the cluster and are coordinated by the SparkContext in the driver program" to "Each Spark application runs as independent process on the cluster coordinated by the SparkContext in the driver program"
Comment 5: Page 3: Line 51: because of a node failure > because of the node failure
Response: Appreciate for your comment, we have changed "because of a node failure" to "because of the node failure"
We tried our best to improve the manuscript and made some changes in the manuscript. These changes will not influence the content and framework of the paper. And here we did not list the changes but marked in revise paper. We appreciate for Editor/Reviewers' warm work earnestly, and hope that the correction will meet with approval. Once again, thank you very much for your comments and suggestions.
Yours
Sincerely
Runxin GUO, Yi ZHAO, Xiangke LIAO, Kenli LI, Quan ZOU, Xiaodong FANG, Shaoliang PENG

Close
