# Supplementary material for: Bioinformatics applications on Apache Spark
Source: Gigascience. 2018 Aug 7;7(8):giy098. doi: 10.1093/gigascience/giy098 (PMC6113509; doi:10.1093/gigascience/giy098)
Supplement: Response_to_Reviewer_Comments_Revision_2.pdf [file giy098_response_to_reviewer_comments_revision_2.pdf]

Author's Response To Reviewer Comments

Close

Dear Editor and Reviewers:
Thank you for your letter and for the reviewers' comments concerning our manuscript entitled "Bioinformatics Application on Apache Spark" (GIGAD1800131R2). Those comments are all valuable and very helpful for revising and improving our paper, as well as the important guiding significance to our research. We have studied the comments carefully and have made correction which we hope meet with approval. Moreover, according to the copy edited manuscript, we have updated the paper for all requested edits.
Yours
Sincerely
Runxin Guo, Yi Zhao, Quan Zou, Xiaodong Fang, Shaoliang Peng

Close
