# Supplementary material for: Bioinformatics applications on Apache Spark
Source: Gigascience. 2018 Aug 7;7(8):giy098. doi: 10.1093/gigascience/giy098 (PMC6113509; doi:10.1093/gigascience/giy098)
Supplement: Reviewer_1_Report_(Original_Submission) -- Brendan Lawlor — 4/29/2018 Reviewed [file giy098_reviewer_1_report_(original_submission).pdf]

Reviewer Report

Title: Bioinformatics applications on Apache Spark

Version: Original Submission **Date:** 4/29/2018

Reviewer name: Brendan Lawlor

Reviewer Comments to Author:

This paper presents Apache Spark as a fast, general-purpose, parallel processing platform suitable for the ever-increasing genomic data generated by NGS. The authors give an overview of Spark's design, compare it to Hadoop, and survey its use in various biological domains. The intention is to serve as a comprehensive guideline for researchers contemplating the use of Spark. The style is clear and quite readable, though some improvements could be made (outlined below). The survey is thorough and a valuable reference and starting point for any researcher wishing to understand the potential impact and application of Spark to their own particular problem domain. In each domain or application (e.g. Sequencing, Assembly, Analysis, Phylogeny), issues with existing implementations are presented (typically problems of speed or scalability) and Spark based alternatives are cited and described. Overall I think the paper is suitable for publication, but with a number of reservations which are detailed below. In particular, I find the paper makes some claims in its Abstract and Key Points which will be hard to defend.The first section refers to general concerns that apply to the paper as a whole. The second section calls out specific issues using page and line numbers. There may be some overlap between the two sections. General concerns:1) The paper would benefit from a *slightly* deeper description of the Spark architecture, in particular explaining the nature of DAGs and the way in which they permits optimizations. Also some mention of the two deploy modes (where the driver program can either be run on the client machine, or on a worker node). 2) The paper would also benefit from a section that examines the potential downsides of using Spark, for example the potential complexity in creating and maintaining a Spark cluster, and the learning curve involved in learning a new API and perhaps even language (especially given the Functional Programming nature of the API).3) With regards to style, there are a number of places in the paper where the definite article is used where it shouldn't, and vice versa. In the interest of readability and not distracting the reader, these should be addressed. A similar point can be made with regard to the over-use of certain prepositions (e.g. "besides"), which are called out in detail in the next section. Specific concerns* p.1 line 28: "data" is treated as a plural in the rest of the paper, therefore "pose" rather than "poses".* p.1 line 34: "by introducing resilient distributed dataset" should be "by introducing the resilient distributed dataset" (i.e. use of definite article)* p.1 line 40: "In the end, we discussed the challenges...and the future work...". I haven't found this discussion in the paper.* p.2 line 4: "MapReduce preforms" should be "MapReduce performs".* p.2 line 21: "introducing resilient distributed dataset" should "introducing the resilient distributed dataset".* p.2 line 38: The documentation of Spark describes the driver program as "The process running the main() function of the application and creating the SparkContext". It does not "deploy the Spark operating environment". Perhaps the authors meant "deploy TO the Spark operating environment" but even here this would be incorrect, as the spark-submit script does this. * p.2, line 43: As well as Scala, Spark provides APIs in Java, Python and more recently R. This flexibility is important to researchers when deciding whether to use Spark or not.* p.2, line 58: It is questionable that "the most important feature of RDD" is the fault tolerance. Certainly it is "an important feature". * p.3, line 13: The referenced image appears to be an _example_ of a spark task flow chart, rather than _the general_ Spark task processing flow. For the reader's sake, the paper should either describe what this particular task is doing (including the fact that it is reading and writing to HDFS in this case). Otherwise the reader may form incorrect opinions or simply be confused. Alternatively, drop the figure entirely.* p.3, line 17: "Besides" as preposition. This is a little colloquial and has an additional "in any case" meaning. To avoid distracting the reader, consider replacing "besides" as a preposition with alternatives like "In addition", "Moreover", "Furthermore". This can be applied to the rest of the paper, and I won't call any more out by line number.* p.4, line 4: "Burrow-Wheeler aligner" - either "The Burrow-Wheeler aligner" or "Burrow-Wheeler alignment" read better.* p.4, line 19: "Results [19] showed" - "The

results [19] showed"* p.4, line 32: "achieved the average speedup of" - "achieved an average speedup of"* p.6, line 58: Drop "And" from the start of the sentence.* p.7, line 1: "Experiments results" - "Experimental results"* p.7, line 4: Perhaps it's worth pointing out that this is an example of the platform itself suggesting a new algorithm, rather than simply reimplementing an existing algorithm on the new platform. Similarly for line 19 of this page.* p.7, line 23: Is SA-BR-MR running on Hadoop? (I ask because MR is a valid algorithm on Spark as well).* p.8, line 17: "Results.." - "The results..."* p.8, line 41: "noises" - "noise".* p.9, lines 23-39: The epigenetics example just calls out the advantage of parallelization compared to sequential processing. Was there a parallelized attempt, perhaps using Hadoop, that the Yu N et al paper could demonstrate a superiority to?* p.10, line 8: "Saprk" - "Spark"* p.10, line 15: The term "checkpointing" is not explained even in the body of the referenced paper (Harnie D et al) and is probably best dropped. * p.11, line 43: Key Points section: I would respectfully disagree with the following statement: "We introduce the Apache Spark framework in detail, helping researchers to understand its architecture, programming model and processing mechanism." I think the authors do a good job of firstly, giving an *overview* of Spark (notwithstanding earlier points about getting into more detail), but I don't think this paper is a *detailed* description of Spark, its architecture or its programming model. Indeed I don't think it *needs* to be - the survey of *how Spark has successfully been used* is probably of primary interest to most readers. But it's best to be clear about the scope of the paper in the Key Points so as to set readers' expectations correctly.* p.11, line 48: Key Points section: Similarly to above, I would edit the the third Key Point to set readers' expectations correctly. The paper in its current form does not include a "discussion on the future of parallel computing in bioinformatics" (and in my opinion it does not need to).

Level of Interest

Please indicate how interesting you found the manuscript: Choose an item.

Quality of Written English

Please indicate the quality of language in the manuscript: Choose an item.

Declaration of Competing Interests

Please complete a declaration of competing interests, considering the following questions:

- Have you in the past five years received reimbursements, fees, funding, or salary from an organisation that may in any way gain or lose financially from the publication of this manuscript, either now or in the future?
- Do you hold any stocks or shares in an organisation that may in any way gain or lose financially from the publication of this manuscript, either now or in the future?
- Do you hold or are you currently applying for any patents relating to the content of the manuscript?
- Have you received reimbursements, fees, funding, or salary from an organization that holds or has applied for patents relating to the content of the manuscript?
- Do you have any other financial competing interests?
- Do you have any non-financial competing interests in relation to this paper?

If you can answer no to all of the above, write 'I declare that I have no competing interests' below. If your reply is yes to any, please give details below.

I declare that I have no competing interests.

I agree to the open peer review policy of the journal. I understand that my name will be included on my report to the authors and, if the manuscript is accepted for publication, my named report including any attachments I upload will be posted on the website along with the authors' responses. I agree for my report to be made available under an Open Access Creative Commons CC-BY license (http://creativecommons.org/licenses/by/4.0/). I understand that any comments which I do not wish to be included in my named report can be included as confidential comments to the editors, which will not be published.

I agree to the open peer review policy of the journal

To further support our reviewers, we have joined with Publons, where you can gain additional credit to further highlight your hard work (see: https://publons.com/journal/530/gigascience). On publication of this paper, your review will be automatically added to Publons, you can then choose whether or not to claim your Publons credit. I understand this statement. Yes
