# Supplementary material for: Bioinformatics applications on Apache Spark
Source: Gigascience. 2018 Aug 7;7(8):giy098. doi: 10.1093/gigascience/giy098 (PMC6113509; doi:10.1093/gigascience/giy098)
Supplement: Reviewer_2_Report_(Original_Submission) -- Joshua W. K. Ho, PhD — 05/04/2018 Reviewed [file giy098_reviewer_2_report_(original_submission).pdf]

Reviewer Report

Title: **Bioinformatics applications on Apache Spark**

Version: **Original Submission** **Date:** 5/4/2018

Reviewer name: **Joshua W. K. Ho, PhD**

Reviewer Comments to Author:

Apache Spark is a big data framework that is increasingly being applied in bioinformatics. A comprehensive survey is needed. This review article fills this important gap. The strength of this manuscript is that it is fairly comprehensive in its coverage of the literature. It has covered papers from both the computer science conference proceedings and biological/bioinformatics journals. Nonetheless, there are a number of major weaknesses:

1. While I agree Spark has a lot of advantages over other parallel and distributed computing frameworks such as MapReduce, I feel the the current tone and content are too one-sided. In my own experience, Spark is mostly only useful for processing very large amount of data. For smaller data sets, the scalability gained by Spark may not be enough to justify the up-front time required for setting up and configuring a Spark-enabled system. Also, there is no discussion on computing hardware requirement (local computer cluster or commercial cloud computing platforms), and issues related to transfer of large data sets over the Internet. All these issues need to be discussed.

2. The sections on 'Spark in motif analysis' and 'Spark in genomic inference'are poorly written. The terms 'motif' and 'genomic inference' are not properly defined. Do they mean transcription factor binding motifs, or simply frequently occurring DNA sequence some defined regions in the genome (e.g., promoters, enhancers, etc)? Also, the term 'genomic data inference' is not well defined. Presumably the authors is referring to inference in a population genomics context.

3. The caption of all four figures are way too simple. In most cases, especially for complicated flow diagrams like Fig 1 and Fig 3, there is no explanation or description of the content of the figure. I believe the authors intends to illustrate the inner working of Spark using these figures. Nonetheless, the content of these figures are not explained in the caption nor the main text.

4. Despite the authors claiming they 'discuss the future of parallel computing in bioinformatics' (Key Points #3), the manuscript barely talks about the future, other than saying 'Spark will provide promising performance for biological researchers in the future' (Conclusions). I think this is a lost opportunity. What do the authors see as the major limitations in the field at the moment? New hardware? Better integration with cloud computing platforms? New application areas, such as proteomics, metabolomics, biomedical text, electronic medical health record, etc...?

Minor concern:
1. In multiple occasions, the authors use 'And' to begin a sentence. I do not think it is grammatically correct.
2. Throughout the manuscript, author names are often cited as ([last name] [first initials]), but sometimes they are cited as ([last name] [all initials]) or ([last name] [first name]). Please make sure names are formatted consistently.

Level of Interest

Please indicate how interesting you found the manuscript: Choose an item.

Quality of Written English

Please indicate the quality of language in the manuscript: Choose an item.

Declaration of Competing Interests

Please complete a declaration of competing interests, considering the following questions:

- Have you in the past five years received reimbursements, fees, funding, or salary from an organisation that may in any way gain or lose financially from the publication of this manuscript, either now or in the future?
- Do you hold any stocks or shares in an organisation that may in any way gain or lose financially from the publication of this manuscript, either now or in the future?
- Do you hold or are you currently applying for any patents relating to the content of the manuscript?
- Have you received reimbursements, fees, funding, or salary from an organization that holds or has applied for patents relating to the content of the manuscript?
- Do you have any other financial competing interests?
- Do you have any non-financial competing interests in relation to this paper?

If you can answer no to all of the above, write 'I declare that I have no competing interests' below. If your reply is yes to any, please give details below.

I declare that I have no competing interests.

I agree to the open peer review policy of the journal. I understand that my name will be included on my report to the authors and, if the manuscript is accepted for publication, my named report including any attachments I upload will be posted on the website along with the authors' responses. I agree for my report to be made available under an Open Access Creative Commons CC-BY license (http://creativecommons.org/licenses/by/4.0/). I understand that any comments which I do not wish to be included in my named report can be included as confidential comments to the editors, which will not be published.

I agree to the open peer review policy of the journal

To further support our reviewers, we have joined with Publons, where you can gain additional credit to further highlight your hard work (see: https://publons.com/journal/530/gigascience). On publication of this paper, your review will be automatically added to Publons, you can then choose whether or not to claim your Publons credit. I understand this statement. Yes
