# Supplementary material for: Bioinformatics applications on Apache Spark
Source: Gigascience. 2018 Aug 7;7(8):giy098. doi: 10.1093/gigascience/giy098 (PMC6113509; doi:10.1093/gigascience/giy098)
Supplement: Reviewer_3_Report_(Original_Submission) -- Tae-Hyuk Ahn — 05/05/2018 Reviewed [file giy098_reviewer_3_report_(original_submission).pdf]

Reviewer Report

Title: Bioinformatics applications on Apache Spark

Version: Original Submission **Date:** 5/5/2018

Reviewer name: Tae-Hyuk Ahn

Reviewer Comments to Author:

Apache Spark is a fast and general-purpose computing framework designed for large-scale data processing. In this work, the authors reviewed Apache Spark based applications in bioinformatics. The authors claims that this survey provides a comprehensive guideline for bioinformatics researchers to apply Spark in their own fields.

Major issues:
1. The manuscript needs to be rewritten to provide more practical information to bioinformatics research users how Apache Spark based bioinformatics tools are actively used in specific bioinformatics research domains and what are the advantages of using the Apache Spark.
2. Table 1 shows basic APIs of Apache Spark. The reviewer cannot get a point why the authors included this table. Delete the table or keep the table with presenting how the APIs could be used in the bioinformatics tools.
3. In the first paragraph of the Introduction section, "However, the existing bioinformatics tools cannot effectively handle such a large amount of data. In order to solve the issues, MapReduce, a programming model for parallel computation of large datasets, has been proposed [1]." sentences should be updated. MapReduce has not been proposed for bioinformatics tool. MapReduce framework was proposed for general purpose big data analysis in the distributed manner.
4. The manuscript did not mention about "DataFrames" API that is an extension of RDD. Most of new Spark features use this new DataFrames, and it should be addressed in the manuscript.
5. Table 2 only shows the application domain, program name, URL, references. The table should be updated to include more meaningful informatics of tools such as pros/cons or specific features or the tools.
6. Figures 1 to 4 are not necessary in the manuscript. Instead of these figures, the authors should consider how to express the relationship of bioinformatics tools and Apache Spark effectively using figures.

Level of Interest

Please indicate how interesting you found the manuscript: Choose an item.

Quality of Written English

Please indicate the quality of language in the manuscript: Choose an item.

Declaration of Competing Interests

Please complete a declaration of competing interests, considering the following questions:

- Have you in the past five years received reimbursements, fees, funding, or salary from an organisation that may in any way gain or lose financially from the publication of this manuscript, either now or in the future?
- Do you hold any stocks or shares in an organisation that may in any way gain or lose financially from the publication of this manuscript, either now or in the future?

- Do you hold or are you currently applying for any patents relating to the content of the manuscript?
- Have you received reimbursements, fees, funding, or salary from an organization that holds or has applied for patents relating to the content of the manuscript?
- Do you have any other financial competing interests?
- Do you have any non-financial competing interests in relation to this paper?

If you can answer no to all of the above, write 'I declare that I have no competing interests' below. If your reply is yes to any, please give details below.

I declare that I have no competing interests.

I agree to the open peer review policy of the journal. I understand that my name will be included on my report to the authors and, if the manuscript is accepted for publication, my named report including any attachments I upload will be posted on the website along with the authors' responses. I agree for my report to be made available under an Open Access Creative Commons CC-BY license (http://creativecommons.org/licenses/by/4.0/). I understand that any comments which I do not wish to be included in my named report can be included as confidential comments to the editors, which will not be published.

I agree to the open peer review policy of the journal

To further support our reviewers, we have joined with Publons, where you can gain additional credit to further highlight your hard work (see: https://publons.com/journal/530/gigascience). On publication of this paper, your review will be automatically added to Publons, you can then choose whether or not to claim your Publons credit. I understand this statement. Yes
